# Analysis of CEPH-accredited DrPH programs in the United States: A mixed-methods study

**DOI:** 10.1371/journal.pone.0245892

**Published:** 2021-02-04

**Authors:** Chulwoo Park, Gene Migliaccio, Mark Edberg, Seble Frehywot, Geralyn Johnson

**Affiliations:** 1 Department of Public Health and Recreation, San Jose State University, San Jose, California, United States of America; 2 Department of Global Health, The George Washington University Milken Institute School of Public Health, Washington, D.C., United States of America; 3 Department of Prevention and Community Health, The George Washington University Milken Institute School of Public Health, Washington, D.C., United States of America; University of Cape Town, SOUTH AFRICA

## Abstract

Interest has been growing in regard to increasing the public health workforce and standardizing training to ensure there are competent professionals to support rebuilding and reinforcing the public health infrastructure of the United States. The need for public health leaders was recognized as early as the hookworm control campaign during 1909–1914 when it became apparent that prevention of disease should be distinct from clinical medicine and should be conducted by professionally trained, dedicated full-time public health practitioners. In recent years, research on the public health workforce and on standardizing health workforce education has significantly expanded. A key element of such a workforce is public health leadership, and DrPH programs are the means to provide effective public health education for these future health professionals. The purpose of this paper is to analyze the general trend of DrPH programs from past to present and analyze the common themes and variations of 28 Council on Education for Public Health (CEPH)-accredited DrPH programs in the United States. This research utilized a mixed-methods approach, investigating DrPH education at each school or program to improve our understanding of the current status of DrPH programs.

## Introduction

### Rationale for establishing the Doctor of Public Health in the United States

The earliest postgraduate training for public health physicians dates back to 1882 in Munich, Germany [1]. Abraham Flexner, an expert and reformer of educational practices and a proponent of the German medical education model of encompassing basic science and clinical training, wrote the Flexner Report of 1910, which adapted the German model and thus stimulated the establishment of a biomedical model for medical training in the United States [2, 3]. A DrPH program was first established in 1909 at the Harvard School of Medicine, which conferred the first DrPH degree in 1911 [4]. Soon after, the Harvard-MIT School for Health Officers was established in 1913 [5].

During the Rockefeller Sanitary Commission (RSC) for the Eradication of Hookworm Disease Campaign (1909–1914) in the southern US states, Wickliffe Rose, Director of the RSC, struggled to find trained and competent public health workers [6]. After realizing that local health officials had downgraded public health to a part-time avocation, he concluded that a new profession, one fully devoted to the control of disease in communities, was needed [6]. Rose insisted that public health work should be separate from medical practice: practicing medicine was defined as curing disease on an individual level, and public health practice was defined as preventing and controlling disease on a population level [6].

From lessons learned during the hookworm control program, development of a template for public health professional education in the United States began in 1915, spearheaded by William Henry Welch’s and Wickliffe Rose’s Welch–Rose Report [7]. This report was submitted to the Rockefeller Foundation, which awarded a grant to Johns Hopkins University in 1916 to make it the first independent graduate school of public health in the United States, headed by Welch as the founding dean [8]. Like Flexner, Welch was inspired by the German approach to medical education—combination of basic science of the preclinical segment and practical aspects of the clinical segment—and implemented this model at Hopkins. According to the Flexner Report of 1910, the Johns Hopkins School of Medicine was identified as a benchmark for all other medical schools in the United States [9]. Likewise, the Johns Hopkins School of Hygiene and Public Health defined the American model of public health education, and other schools in the United States began to adopt this model [10].

### Development of Doctor of Public Health education

Efforts to officially standardize DrPH training date back to 1919. At the 48th American Public Health Association (APHA) annual meeting in New Orleans, a committee of 16 representatives of Eastern universities took the first step toward the standardization of public health professional training by creating “duly recognized and accredited degrees” [11, 12]. By 1920, a total of nine DrPH programs were identified [11]. Although almost all schools required a Doctor of Medicine degree as a prerequisite for the DrPH degree, the required time for graduation was not standardized, ranging from merely 36 hours (a few weeks’ course) to as much as 3 years. The committee, therefore, thought the most desirable way to standardize public health education was to set up a common time requirement for the DrPH degree. At the time that DrPH programs were introduced, physicians received no formal training regarding disease prevention or general health promotion [13]. After World War II, the idea of “preventive and social medicine,” emerged in England and stressed the social determinants of disease for health care and attempted to bridge clinical and social medicine [5, 14, 15]. This concept was applied to US schools of public health in the form of studies in the economics, social, and administrative aspects of medical care [1, 16].

In 1953, a group of seven schools of public health founded the Association of Schools of Public Health (ASPH) to enhance academic public health programs and develop standards and definitions for schools of public health [17, 18]. From 1945 to 1973, the APHA accredited graduate professional education in public health [19]. Then, in 1974, the APHA and ASPH joined forces to establish an independent Council on Education for Public Health (CEPH) to improve the quality of public health education and evaluate schools of public health [19]. In 2013, the ASPH changed its name to the Association of Schools and Programs of Public Health (ASPPH) to embrace public health programs in addition to schools of public health [20].

**Fig 1:**
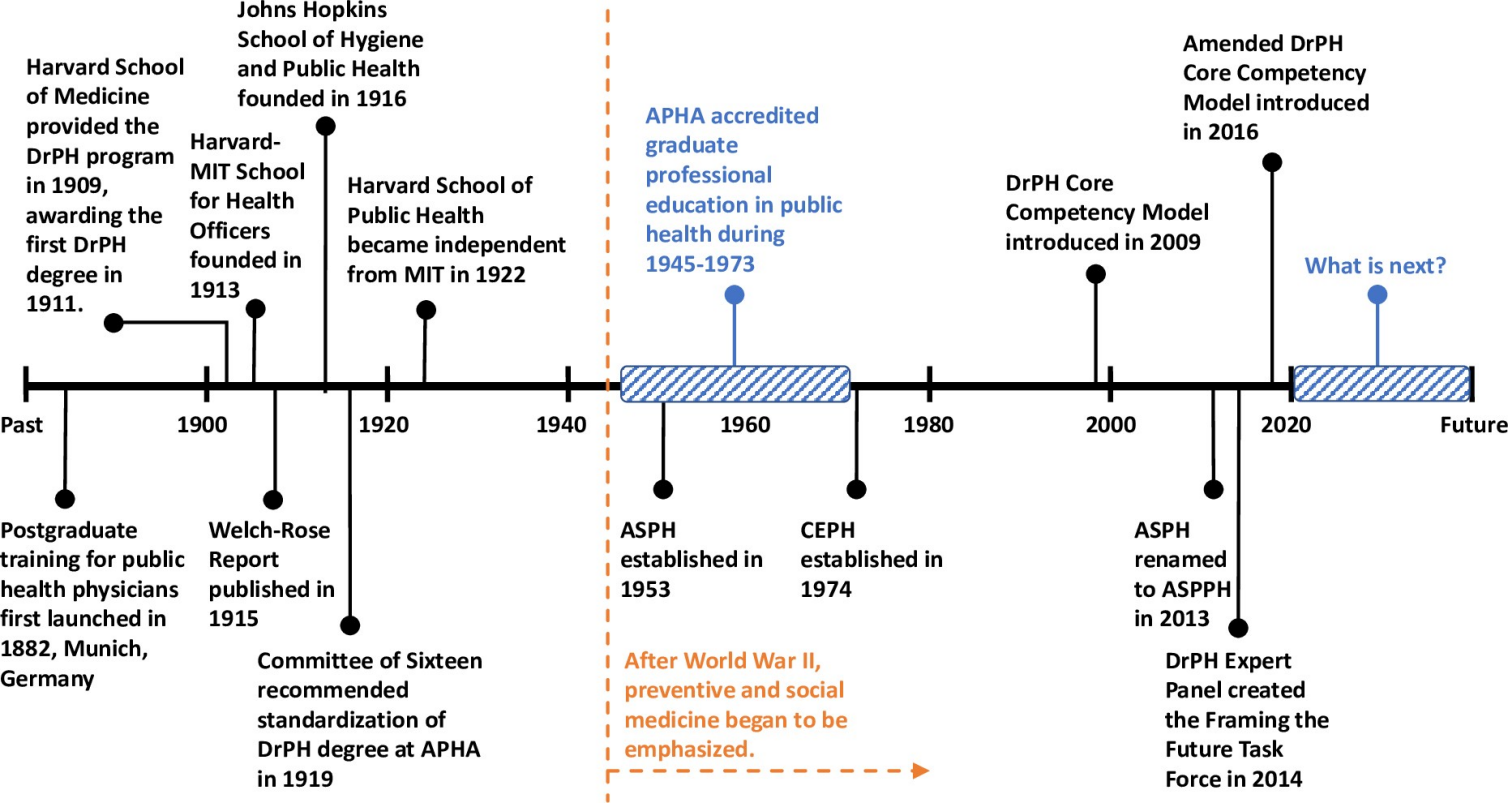
The timeline of the development of the DrPH degree.

In January 2014, the ASPPH assembled a DrPH Expert Panel which created the Framing the Future Task Force to discuss a vision for the DrPH degree in the 21st century [21]. Then, in October 2016, the CEPH produced amended accreditation criteria that superseded the 2009 DrPH core competency model, with 20 competencies in 4 domains: data and analysis, leadership, management and governance, policy and programs, and education and workforce development [22]. Fig 1 summarizes the timeline for the development of the DrPH degree. The ongoing development and refinement of the DrPH degree and educational programs over the years has made it distinct and specialized.

## Materials and methods

This research utilized a mixed-methods study, investigating DrPH education at each sampled school or program to improve our understanding of the structure of DrPH programs. For this study, the results of the quantitative method as the first phase were sequentially used to inform the development of the qualitative method as the second phase [23]. The purpose of using a mixed-methods research design for this study was not only to identify the key issues that needed additional in-depth explanation but also to get participants’ perspectives, given the key characteristics and context of current programs. Collecting and analyzing quantitative data from available secondary data sources as the first phase allowed informed development of a questionnaire for semi-structured, in-depth interviews with DrPH directors as the second phase.

### Target DrPH programs

This study included all DrPH programs in the United States and its territories that were CEPH-accredited and actively recruiting students for the 2020–2021 academic year for the following two reasons: First, a convenience sampling strategy was used to select the programs with information publicly available from school webpages, ASPPH, and CEPH information on CEPH-accredited DrPH programs. CEPH is a member of the Association of Specialized and Professional Accreditors, a unified voice for programmatic accreditation in the United States, which ensures higher education standards and delivery in public health [24–26]. A group of CEPH-accredited DrPH programs has already satisfied the higher-level education standard through a rigorous review process, as instructed by CEPH. Thus, this sample group represents DrPH programs in the United States and its territories approved by the largest and most respected accrediting body in the field of public health.

**Fig 2:**
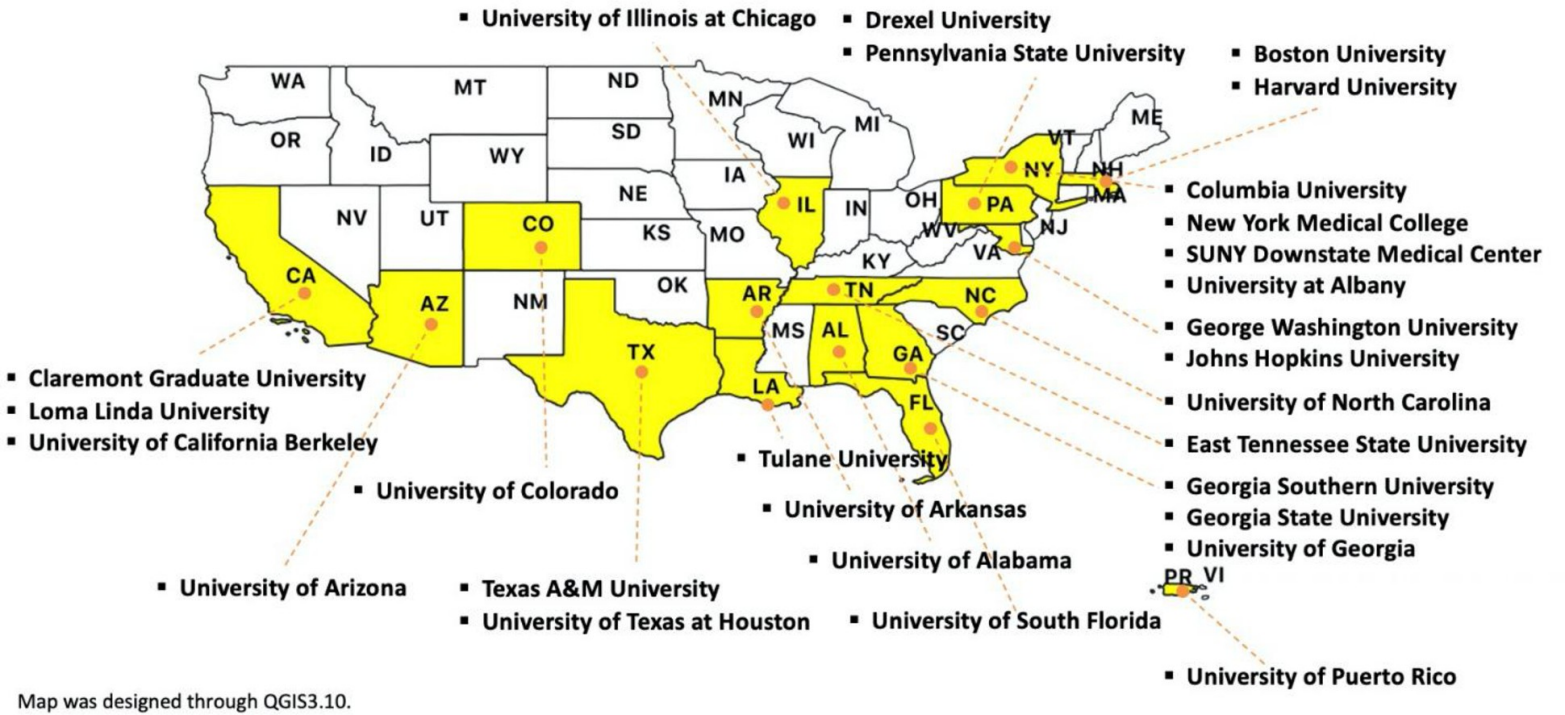
Distribution of DrPH programs in the United States (as of February 2020).

To identify CEPH-accredited DrPH programs, ASPPH’s Academic Program Finder was mainly used [27]. As of February 2020, the ASPPH’s Academic Program Finder listed a total of 30 CEPH-accredited DrPH programs. This list was then cross-checked with the list of accredited schools and programs provided by CEPH [28]. Manual searching was then conducted to determine whether other DrPH programs accredited by CEPH existed. Table 1 presents the final list of 28 CEPH-accredited DrPH programs. These programs are located in 16 states (Alabama, Arizona, Arkansas, California, Colorado, Florida, Georgia, Illinois, Louisiana, Maryland, Massachusetts, New York, North Carolina, Pennsylvania, Tennessee, and Texas), the District of Columbia, and Puerto Rico (Fig 2). The TIGER/Line Shapefile from US Census Bureau was used for the borderline of the United States map [29]. Geographic information was designed through QGIS 3.10, an open-source geographic information system application [30].

**Table 1:** CEPH-accredited DrPH programs (This list is accurate as of February 2020).

University or School Name (in alphabetical order)	City, State
1. Boston University School of Public Health	Boston, Massachusetts
2. Claremont Graduate University School of Community & Global Health	Claremont, California
3. Colorado School of Public Health	Aurora, Colorado
4. Columbia University Mailman School of Public Health	New York, New York
5. Drexel University Dornsife School of Public Health	Philadelphia, Pennsylvania
6. East Tennessee State University College of Public Health	Johnson City, Tennessee
7. George Washington University Milken Institute School of Public Health	Washington, DC
8. Georgia Southern University Jiann-Ping Hsu College of Public Health	Statesboro, Georgia
9. Georgia State University School of Public Health	Atlanta, Georgia
10. Harvard T. H. Chan School of Public Health	Boston, Massachusetts
11. Johns Hopkins Bloomberg School of Public Health	Baltimore, Maryland
12. Loma Linda University School of Public Health	Loma Linda, California
13. New York Medical College School of Health Sciences	Valhalla, New York
14. Pennsylvania State University College of Medicine Public Health Program	Hershey, Pennsylvania
15. SUNY Downstate Medical Center School of Public Health	Brooklyn, New York
16. Texas A&M School of Public Health	College Station, Texas
17. Tulane University School of Public Health and Tropical Medicine	New Orleans, Louisiana
18. University at Albany School of Public Health	Rensselaer, New York
19. University of Alabama at Birmingham School of Public Health	Birmingham, Alabama
20. University of Arizona Mel and Enid Zuckerman College of Public Health	Tucson, Arizona
21. University of Arkansas Fay W. Boozman College of Public Health	Little Rock, Arkansas
22. University of California Berkeley School of Public Health	Berkeley, California
23. University of Georgia College of Public Health	Athens, Georgia
24. University of Illinois at Chicago School of Public Health	Chicago, Illinois
25. University of North Carolina Gillings School of Global Public Health	Chapel Hill, North Carolina
26. University of Puerto Rico Graduate School of Public Health	San Juan, Puerto Rico, United States
27. University of South Florida College of Public Health	Tampa, Florida
28. University of Texas Health Science Center at Houston School of Public Health	Houston, Texas

### Quantitative analysis

For the quantitative analysis, the following data sources were explored: the ASPPH Data Center Portal, school websites and their recent DrPH curricula, guidelines, and graduate student handbooks (2018–2019 and 2019–2020 academic years). Among these data, the following specific components were analyzed: the application trends; acceptance; new enrollment; fall enrollment; completion rates by gender, citizenship, and race/ethnicity from 2010 to 2019; and graduates’ employment and continuing education during the 2016–2018 period relevant to DrPH programs. Note that the list of ASPPH members each year from 2010 to 2019 in the ASPPH Data Center Portal was not limited to 28 identified CEPH-accredited DrPH programs in 2020 because DrPH programs have continually been removed or created each year.

In addition, information was collected from school websites, DrPH curricula, guidelines, and student handbooks for each of the DrPH programs. From that information, the following data were analyzed: DrPH degree type (schoolwide, departmental, departmental-hybrid, or concentration), DrPH program specialties and concentration, DrPH program prerequisites and application requirement, DrPH program structure (cohort size, semester vs. quarter, part-time vs. full-time), allowed transfer credit, credits needed for graduation, program length, DrPH program curriculum and coursework requirements, required leadership courses, comprehensive/qualifying exam, applied practice experience (practicum or internship), dissertation, and DrPH program internal funding.

### Qualitative analysis

The purpose of using a qualitative analysis for the second phase was to supplement the secondary data from the quantitative component and obtain perspectives from DrPH academic leaders in each of the DrPH programs. The qualitative portion of this study was exempted from Institutional Review Board (IRB) review under DHHS regulatory category 2 (IRB# NCR191841). The exemption was granted from The George Washington University Committee on Human Research, IRB, FWA00005945. The verbal informed consent was obtained from all participants.

A questionnaire was developed to guide a 15–30-minute interview. The lead author conducted a total of 21 in-depth interviews as a sole interviewer. The interviewer’s credential at the time of the study was a DrPH candidate and had experience of conducing a number of qualitative studies. In-depth interviews were conducted with 21 DrPH directors, representing 68% of the DrPH programs (19/28). In cases where more than one person at an institution was interviewed, the second participant from the same institution was not asked about specific program details; rather, they were asked primarily about perspectives and professional opinions about the institution’s DrPH program and DrPH education across the nation. Table 2 shows an interview guide that was used for asking common themes and variations of DrPH programs in the United States to interviewees.

**Table 2:** Interview guide for DrPH program directors.

Topics	Questions
Before starting: For the purpose of transcription, may I record our conversation?	
**1. Strength**	What are the strengths of the DrPH program at your school, and how could these strengths be used to successfully educate and empower the future leaders of public health? (Prompt: If interviewee can’t answer this right away, the following prompts are recommended: school location, access to public health work, priorities, core expertise, or any other factor).
**2. Weakness**	What challenges do you face in the DrPH program? (Prompt: If interviewee can’t answer this right away, the following prompts are recommended: financial aid, tuition costs, faculty capacity, attention to mentoring DrPH students, or any other factor).
**3. Program Type, Residency Requirement, and Students**	3-1-1. For schoolwide DrPH directors: What is the relationship between the DrPH program and the other departments? I’m wondering whether the interdisciplinary DrPH program communicates and collaborates with other departments to ensure interdepartmental doctoral-level education for DrPH students.
	3-1-2. For departmental-based DrPH directors: I am wondering about the relationship between the DrPH program in your department and DrPH programs from other departments?
	3–2. How many students are recruited annually? What is the total program size?
	3–3. How do students normally find and complete the practicum? How do you decide whether the applied practice experience (practicum) differs substantially from a student’s current job description? Or do you allow students to complete the practicum from their current job?
	* For schools/programs that did not provide information: What are the minimum hours or credits to complete the practicum?
	3–4. Leadership, management, and governance are some of the DrPH foundational competencies in CEPH criteria. How has your DrPH program focused on its leadership course? How have leadership courses been developed?
	3–5. For schools that accept both part-time and full-time students: part-time and full-time students may have different expectations and perspectives. How has your DrPH curriculum tried to satisfy all students who are in the same class?
	*General: How has your DrPH curriculum tried to satisfy all students in the same class who may have different expectations and perspectives?
	3–6. For schools that accept both part-time and full-time students: How would you like to further develop the curriculum to embrace both part- and full-time students as well as students from various backgrounds who have different expectations and perspectives?
	*General: How would you like to further develop the curriculum to embrace students from various backgrounds who have different expectations and perspectives?
	3-7-1. For schoolwide DrPH directors: Many of the DrPH programs in different schools are still departmentally based. How was your DrPH program determined to be interdepartmental? I would like to hear your opinion about schoolwide DrPH programs vs. departmental-based DrPH programs.
	3-7-2. For departmentally based DrPH directors: Some of the departmentally based DrPH programs have or will have changed to schoolwide DrPH programs. Would your DrPH program be maintained as departmentally based? I would like to hear your opinion about schoolwide DrPH programs vs. departmentally based DrPH programs.

Twenty interviews were audio-recorded, and the average length of the interviews was 24 minutes (ranging from 13 to 40 minutes). The main interview modalities were by phone (9%, 20/21), followed by in person (5%, 1/21) and online Zoom audio conference call (5%, 1/21). NVivo 12 Plus for Windows (QSR International, Pty, Ltd.) was used to code and analyze 20 verbatim transcriptions. Transcripts were coded using a two-step process: deductive approach and inductive approach. For a deductive approach to coding, a list of predefined codes was created in the initial codebook, and units of data were coded to the relevant nodes. Subsequently, during the inductive approach to coding, units of data were attached to the new codes, and the collected data were analyzed and then coded to the newly generated nodes in the final codebook. Quotations used in the analysis or results sections are anonymous and cannot be linked to a particular individual, program, or institution.

## Results

### Quantitative analysis: Common themes and variations

The results of the quantitative analysis were organized into the following six common themes and variations: (1) DrPH admissions to completion by ASPPH members (2010–2019), (2) DrPH program structure, (3) DrPH program didactic curriculum, (4) DrPH culminating experience, (5) DrPH funding opportunities, and (6) DrPH graduate outcomes. In addition, S1 to S8 Tables in the supporting information file showed all the details regarding common themes and variations from each of the DrPH programs.

#### DrPH admissions to completion by ASPPH members (2010–2019)

**Fig 3:**
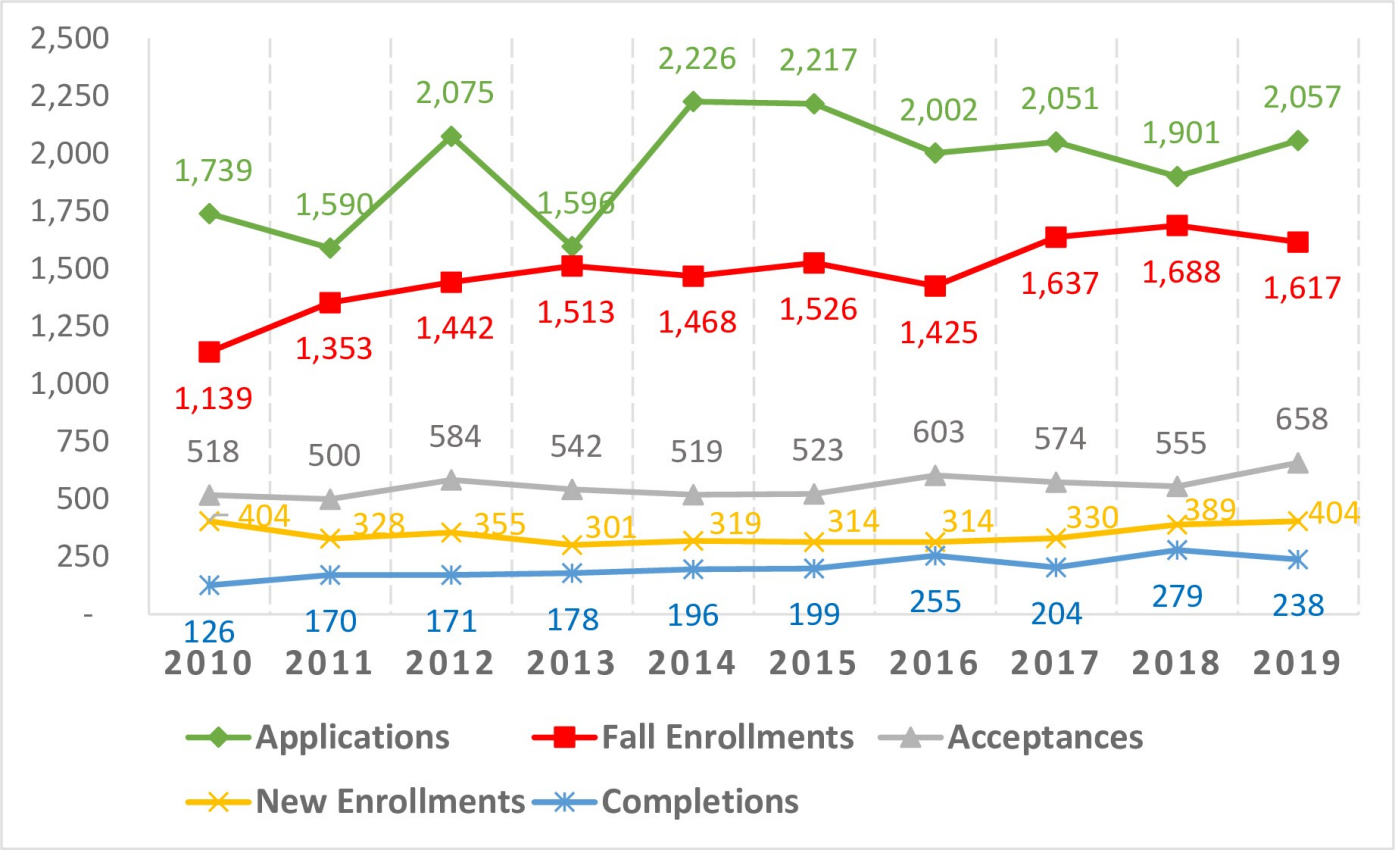
DrPH applications to completions (2010–2019).

The applications, new enrollments, fall enrollments, and completions data during 2010–2019 were collected and arrayed in the form of a time series (Fig 3). In the last 10 years, the total number of DrPH applications was 19,454. Among those applications, the total number of DrPH acceptances was 5,576. The total number of DrPH new enrollments during that time period was 3,390.

**Fig 4:**
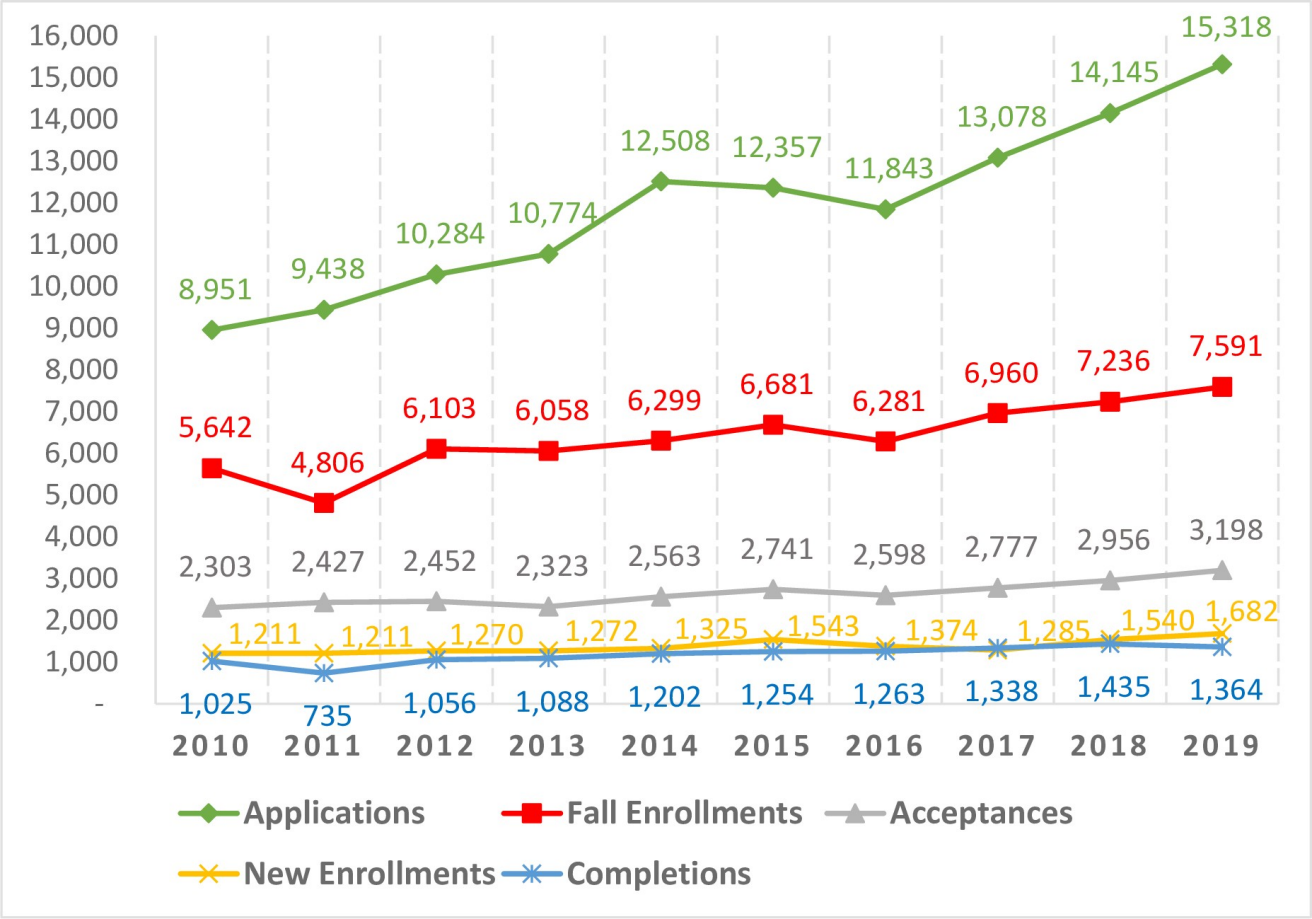
All other doctorates applications to completions (2010–2019).

Then, another time series graph was created for all other doctorates (PhD, ScD, Joint/Dual) (Fig 4) to compare that information with the following five components: DrPH applications, acceptances, new enrollment, fall enrollment, and completions. Overall, the number of students in all other doctorates in the public health program was from 4.1 times to 6.1 times higher than that in the DrPH degree.

Table 3 shows that almost 70% of new enrollments were female. Other/unknown includes individuals who reported their gender classifications as “Other than male or female” or did not report their gender classifications.

**Table 3:** DrPH admissions to completions by gender (2010–2019).

	Female	Male	Other/unknown	Total
Application	13,072 (67.2%)	6,317 (32.5%)	67 (0.3%)	19,456 (100%)
Acceptance	3,739 (67.1%)	1,820 (32.6%)	17 (0.3%)	5,576 (100%
New enrollment	2,341 (69.1%)	1,045 (30.8%)	4 (0.1%)	3,390 (100%)
Completion	1,442 (71.5%)	572 (28.4%)	2 (0.1%)	2,016 (100%)

*Due to an incorrect dataset from the University of Pittsburgh Graduate School of Public Health in 2011 (female: 27, male: 5, total: 31) and 2013 (female: 42, male: 12, total: 53), the total number of applications by gender was 19,456, which is different from 19,454 applications in general.

Table 4 displays the accumulated DrPH applications, acceptances, new enrollments, and completions by citizenship during the 2010–2019 period. US citizens or residents were defined as “individuals who are citizens or residents of the United States,” and foreign nationals were defined as “individuals who are neither citizens nor residents of the United States.” Among the 5,576 acceptances, 4,175 were US citizens/residents (74.9%), and their proportions of new enrollment (79.3%) and completion (82.7%) gradually increased compared with those of foreign nationals, implying that fewer foreign nationals who were accepted and matriculated successfully completed their DrPH degrees than US citizens/residents.

**Table 4:** DrPH admissions to completions by citizenship (2010–2019).

	US citizens/residents	Foreign nationals	Total
Application	13,784 (70.9%)	5,671 (29.2%)	19,455 (100%)
Acceptance	4,175 (74.9%)	1,400 (25.1%)	5,576 (100%)
New enrollment	2,688 (79.3%)	702 (20.7%)	3,390 (100%)
Completion	1,688 (82.7%)	348 (17.3%)	2,016 (100%)

*Due to an incorrect dataset from the University of Pittsburgh Graduate School of Public Health in 2011 (US citizens/residents: 35, foreign nationals: 19, total: 53), the total number of applications by citizenship was 19,455, which is different from 19,454 applications in general.

For race and ethnicity data, ASPPH followed Integrated Postsecondary Education Data System guidelines from the National Center for Education Statistics [31]. The largest accepted group was White (32.3%), followed by non-US citizens (25.1%) and Black or African American (15.8%) (Table 5). Reflecting the proportions of acceptance, White was the group that most often completed the degree (41.7%), followed by non-US citizens (17.3%) and Black or African American (15.7%). The proportion of acceptance among non-US citizens (25.1%) significantly dropped to 17.3% of degree completion rate.

**Table 5:** DrPH admissions to completions by race and ethnicity (2010–2019).

	Hispanic/Latino	American Indian/Alaska native	Asian	Black or African American	Native Hawaiian/Pacific Islander	White	Two or more races	Race unknown	Non-US citizens	Total
Application	1,400 (7.2%)	122 (0.6%)	1,844 (9.5%)	3,906 (20.1%)	41 (0.2%)	4,494 (23.1%)	448 (2.3%)	1,532 (7.9%)	5,671 (29.2%)	19,454 (100%)
Acceptance	455 (8.2%)	28 (0.5%)	487 (8.7%)	883 (15.8%)	8 (0.1%)	1,796 (32.3%)	147 (2.6%)	373 (6.7%)	1,400 (25.1%)	5,576 (100%)
New enrollment	332 (9.8%)	18 (0.5%)	288 (8.5%)	566 (16.7%)	6 (0.2%)	1,210 (35.7%)	106 (3.1%)	162 (4.8%)	702 (20.7%)	3,390 (100%)
Completion	178 (8.8%)	9 (0.4%)	186 (9.2%)	316 (15.7%)	10 (0.5%)	841 (41.7%)	40 (2%)	88 (4.4%)	348 (17.3%)	2,016 (100%)

#### DrPH program structure

**Fig 5:**
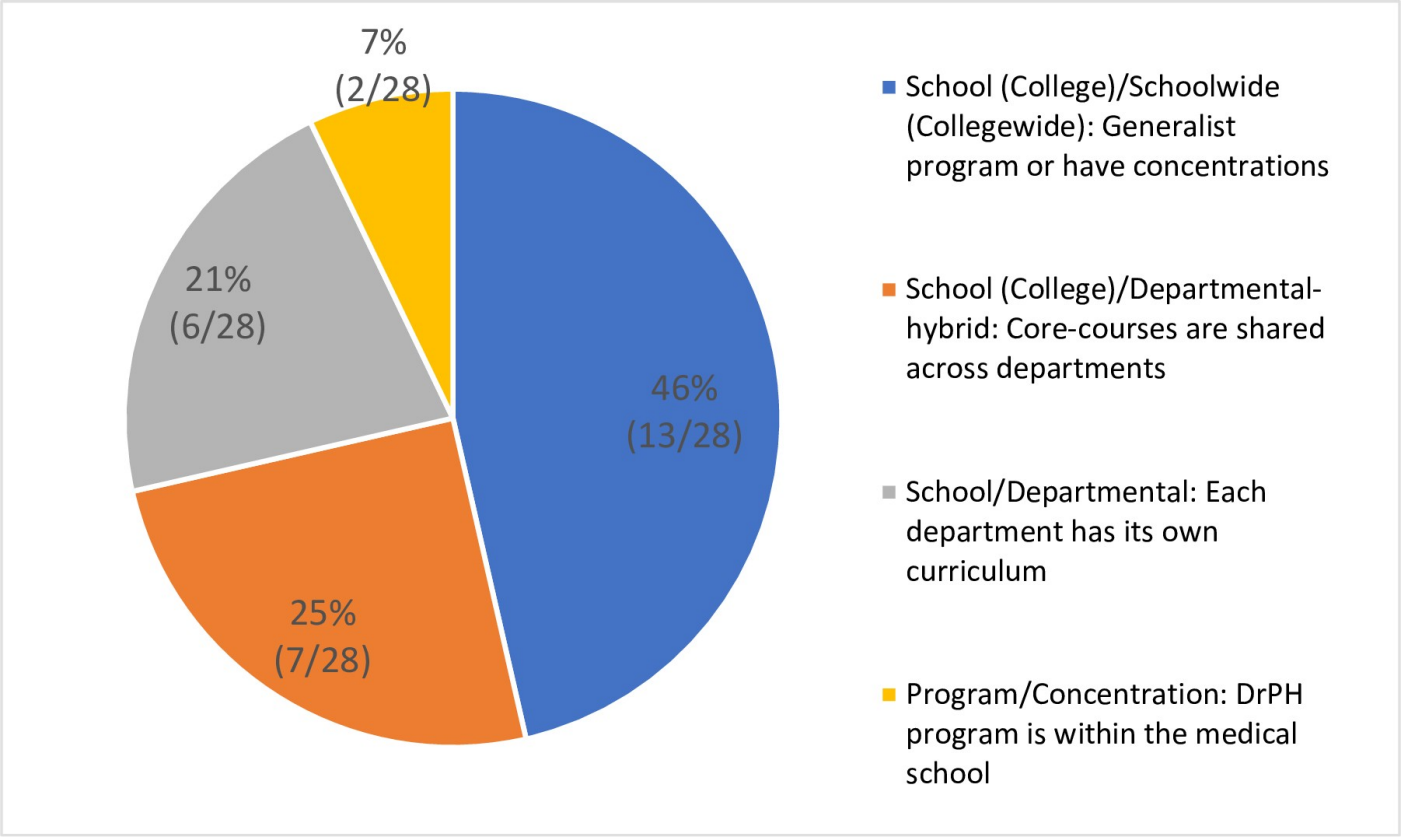
Types of DrPH programs in CEPH-accredited schools in the United States.

*DrPH program format options (program type)*. The DrPH degree is typically offered through a public health program, through its own department, or schoolwide. Types of DrPH programs can be divided into the following formats: (1) school (college)/schoolwide (collegewide); (2) school (college)/departmental–hybrid; (3) school/departmental; and (4) program/concentration (Fig 5).

**Fig 6:**
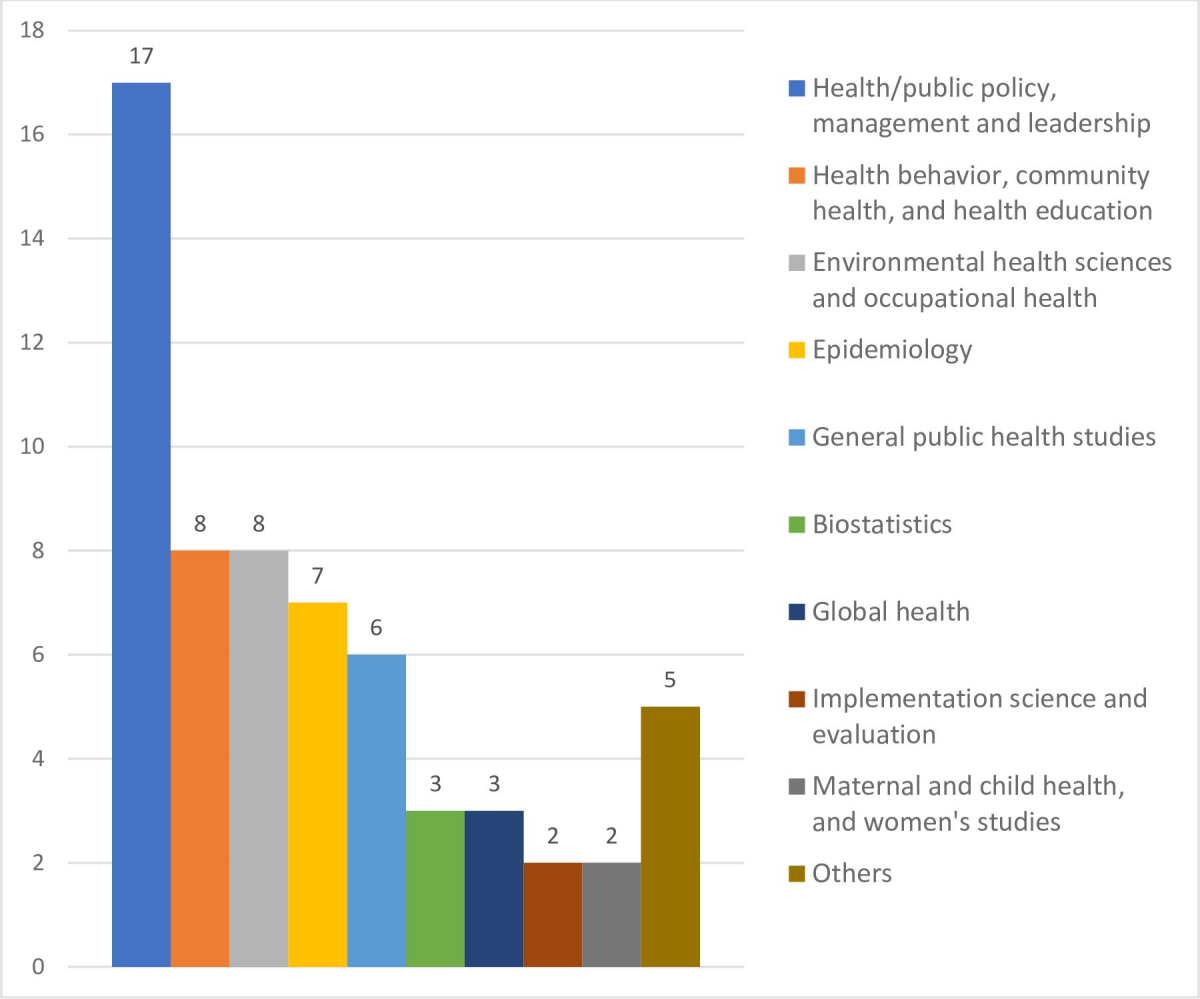
Distribution of specialization area or expertise from DrPH programs.

*Public health specialization area or expertise*. Referring to public health specialization area or expertise and reviewing all of the current program foci from Beck et al. (2018) and ASPPH (2018) [32, 33], the areas of study in the DrPH program were classified into the following 10 areas: general public health studies (nonspecialized); biostatistics; epidemiology; environmental health sciences and occupational health; health/public policy, management, and leadership; health behavior, community health, and health education; global health; implementation science and evaluation; maternal and child health and women’s studies; and others. The criterion for finalizing the list of areas was whether the area included at least two schools or programs. Following the ASPPH’s classification, if the specialization was from an epidemiology and biostatistics combined program, the data were recorded as in the area of epidemiology (Fig 6).

**Fig 7:**
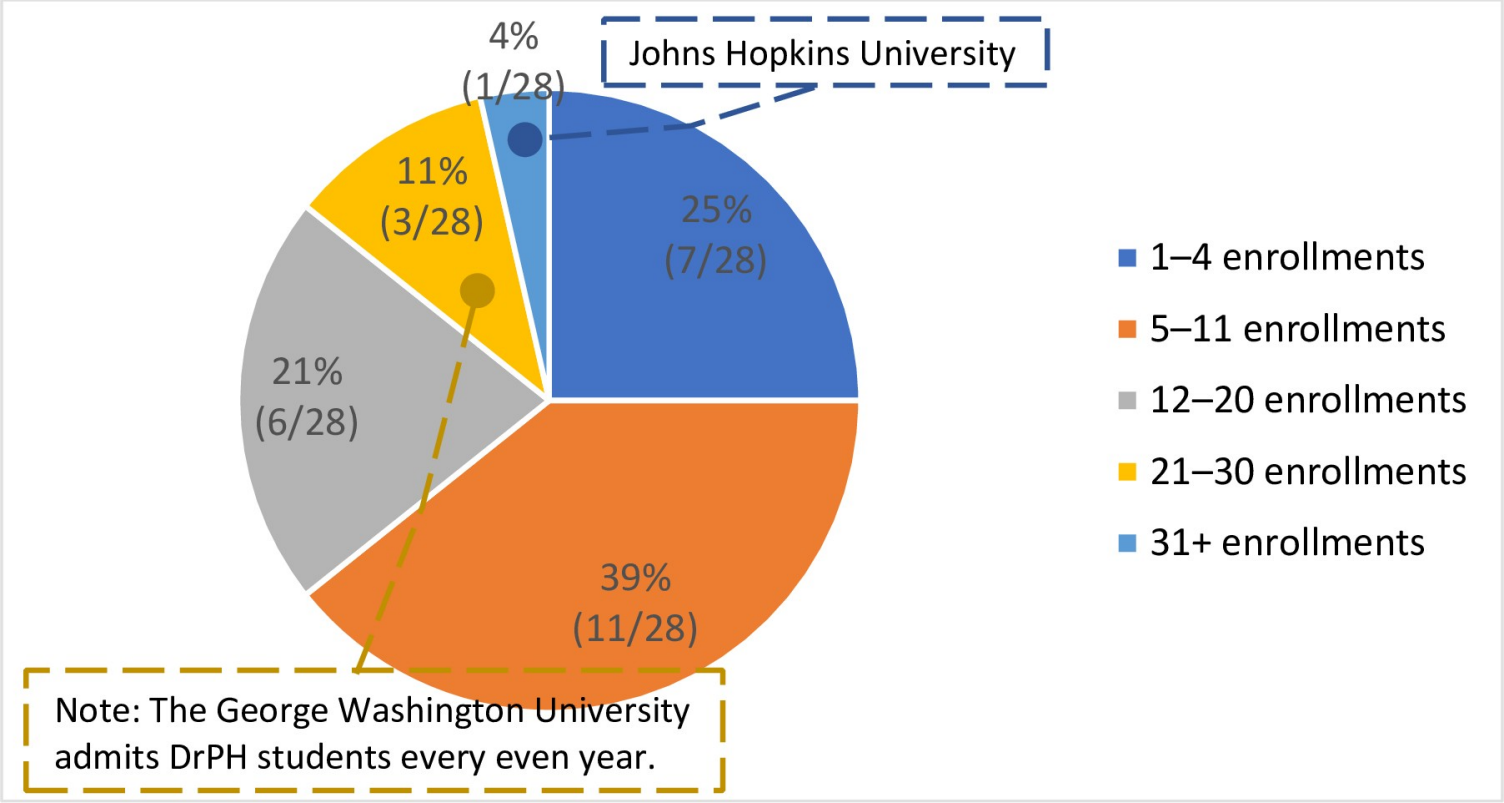
Size of enrolled student group or cohort per year.

*Size of student group or cohort*. Enrolled student group or cohort size per year varied across schools or programs (Fig 7). Notably, The George Washington University Milken Institute School of Public Health admits students every even year, whereas all other schools recruit students every year. The Johns Hopkins Bloomberg School of Public Health has the largest student group. The school currently has 144 DrPH students and plans to recruit 75–90 students for the 2020–2021 academic year, which would result in a maximum program size of 234.

*Semester vs*. *quarter (term)*. Although 24 schools (86%) have adopted semester-based education, 4 schools offer term- or quarter-based education (14%): Drexel University Dornsife School of Public Health, Johns Hopkins Bloomberg School of Public Health, Loma Linda University School of Public Health, and University of Puerto Rico Graduate School of Public Health.

**Fig 8:**
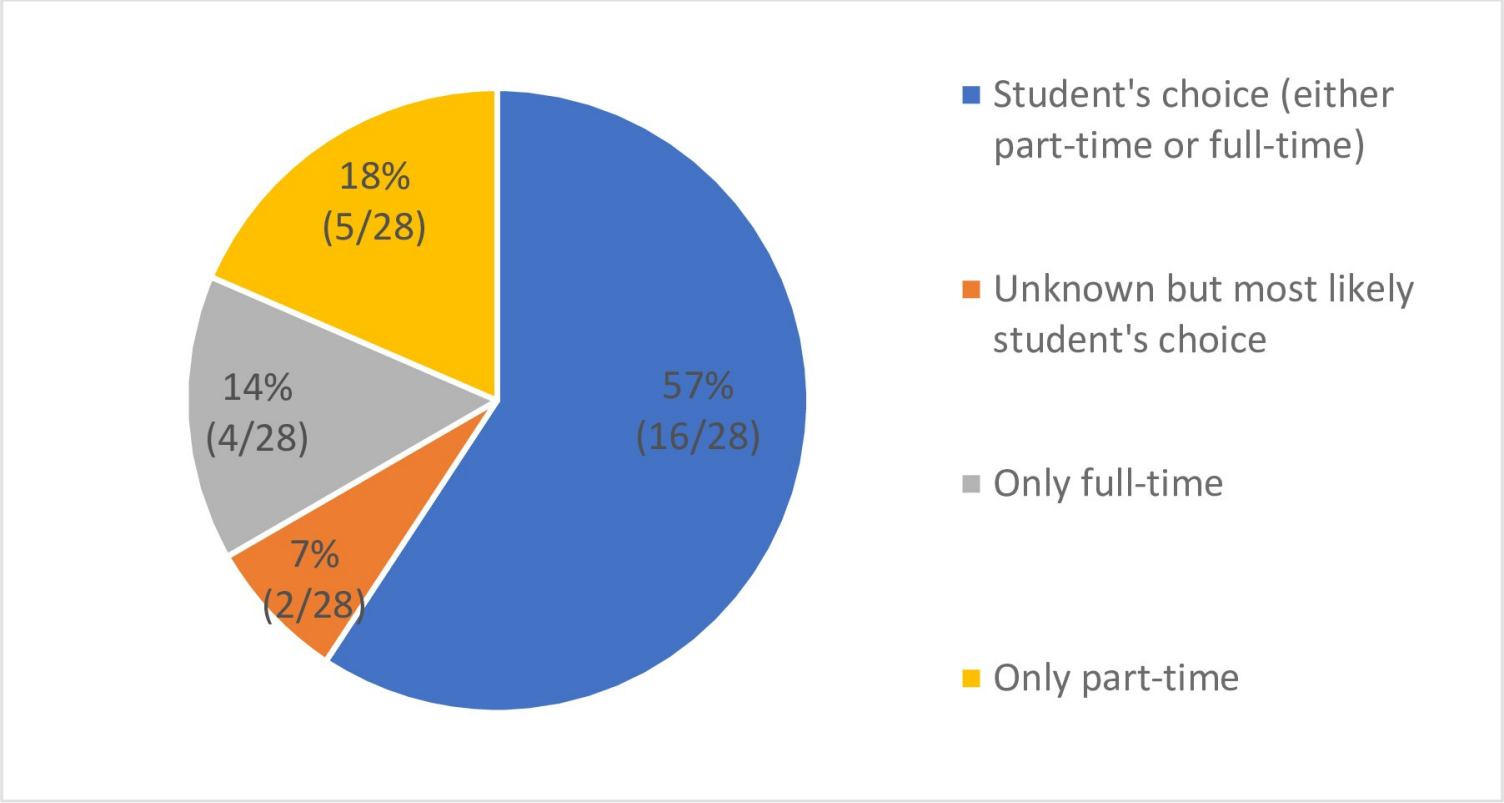
Residency requirement for coursework.

*Part-time vs*. *full-time study*. Fig 8 demonstrates the distribution of residency requirements for coursework. Overall, 19 DrPH programs allow students to choose how they want to approach their DrPH study either on a part- or full-time basis.

*Program length*. The minimum program length at any of these schools is 3 years, and the maximum program length is 7–10 years. The amount of time needed does not depend on whether the student goes full time or part time. Three schools have designed a fixed 3 to 4-year DrPH program (11%): the Harvard T. H. Chan School of Public Health (based on full-time enrollment), the University of California Berkeley School of Public Health (based on full-time enrollment), and the University of North Carolina Gillings School of Global Public Health (based on part-time enrollment). All other schools or programs have a maximum of allowed years, from 7 to 10 years, because graduation depends on students’ progress through the completion of their dissertation.

#### DrPH program didactic curriculum

*Prerequisites (MPH degree and work experience) and application requirements*. In September 2014, the ASPPH’s DrPH Expert Panel published the first DrPH degree report, *DrPH for the 21st Century*, since the ASPPH’s release of the DrPH Core Competency Model in 2009 [34].

Overall, 23 out of 28 schools (82%) require an MPH or equivalent master’s degree from a CEPH-accredited or another regionally accredited program as a prerequisite. Admitted students who do not have an MPH degree still must take between one and five core MPH courses. More than half of the schools or programs (54%, 15/28) require work experience in the field of public health. Some of them specifically ask for several years of postgraduate work experience or full-time work experience.

**Fig 9:**
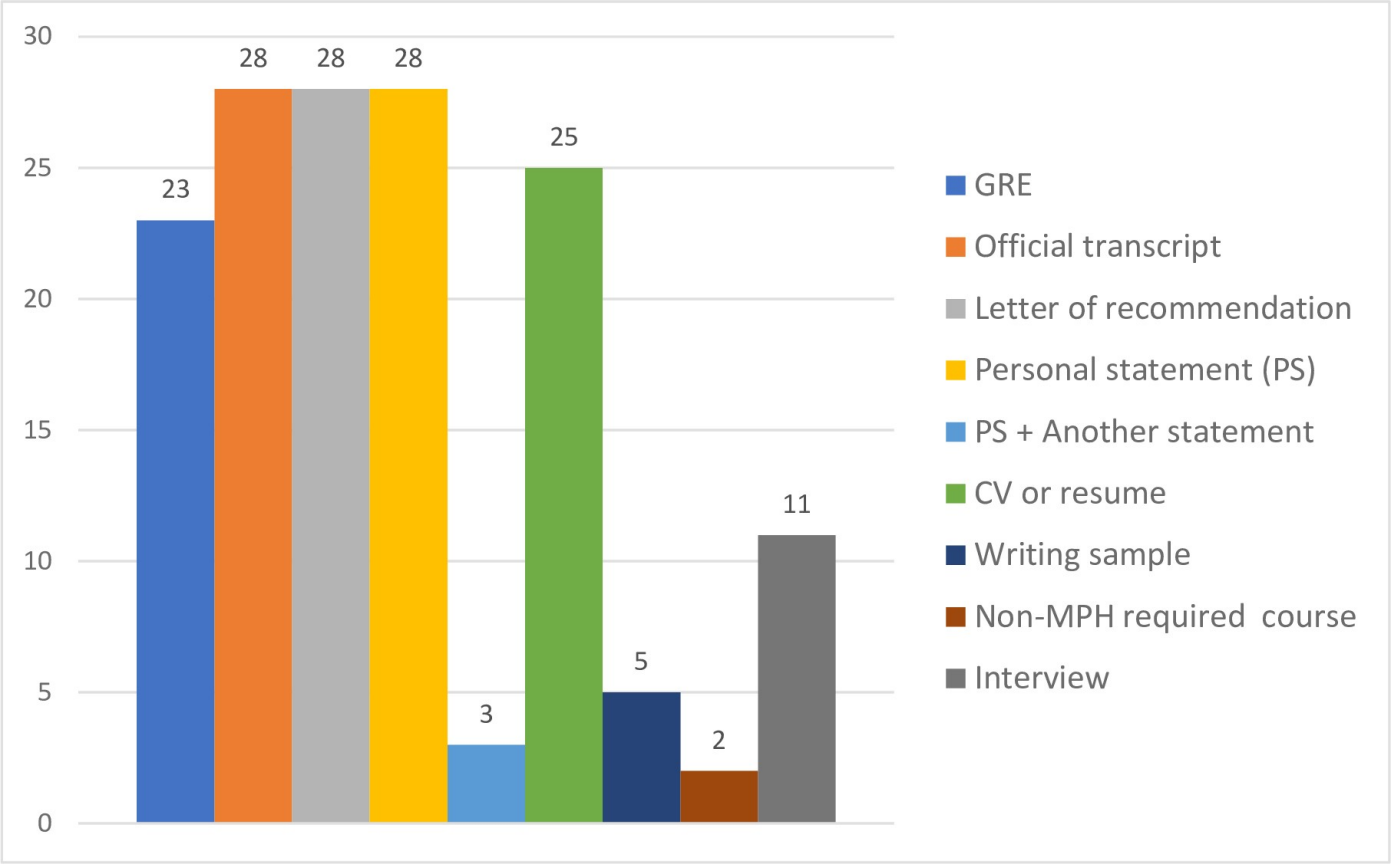
Requirements of the DrPH application.

Regarding application requirements, most schools or programs require five sets of documents: (1) GRE scores, (2) official transcripts, (3) three letters of recommendation, (4) personal statement (statement of experience, purpose, and objectives), and (5) CV or resume (Fig 9). There is a growing trend where the GRE is not a requirement, is optional, or can be waived (required: 61%, 17/28; not required: 18%, 5/28; optional: 21%, 6/28). The personal statement was indicated differently at different schools or programs: as a statement of purpose and objective(s); statement of purpose; personal statement; written statement of career goals; (personal) essay; written statement; career statements; advanced practice statement; policy statement; statement of experience, purpose, and objectives; or statement of purpose and degree objectives. In addition to the personal statement, 4 DrPH programs require an additional statement, such as a mission and values statement, doctoral program interests, a personal history statement, and a statement of public health experience; 4 DrPH programs require a writing sample; and 12 DrPH programs require a personal interview with the applicants, either in person or online.

*Coursework*: *Campus-based vs*. *distance-based*. Although 24 DrPH programs provide campus-based (on-site) courses, coursework at the following four programs—Johns Hopkins Bloomberg School of Public Health, University of Illinois at Chicago School of Public Health, University of North Carolina Gillings School of Global Public Health, and University of South Florida College of Public Health—is exclusively or primarily distance-based (online), with a small portion of residency requirements (campus-based: 86%, 24/28; distance-based: 14%, 4/28).

**Fig 10:**
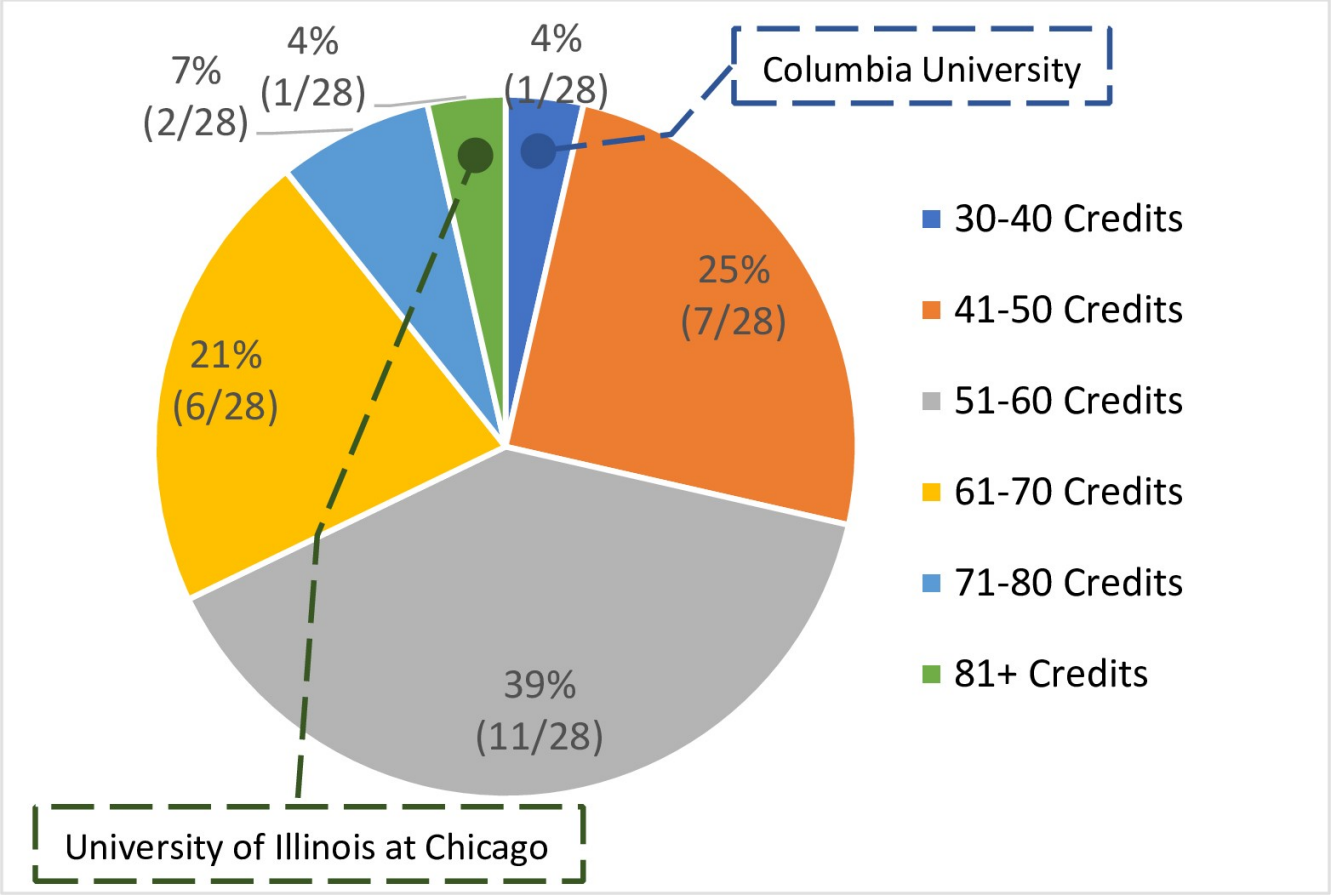
Credits/units needed for graduation.

*Credits/units needed for graduation*. According to the CEPH accreditation criteria, a minimum of 36 didactic semester-credits of post-master’s coursework or its equivalent, except for MPH-level prerequisites, the applied practice experience (practicum), and the integrative learning experience (written product), are required for the DrPH degree [22]. Research has shown that the total number of minimum credits needed for graduation, including post-master’s coursework, residency, internship, and other applied practice experience, ranged from 30–36 credits plus continuous registration (Columbia Mailman School of Public Health) to 96 credits (University of Illinois at Chicago School of Public Health). The Columbia University Mailman School of Public Health requires the completion of 30–36 coursework credits and continuous registration (one credit per semester) until completion (Fig 10). Aligning with the CEPH accreditation, schools require additional coursework for a non-MPH graduate.

Overall, there were two different characteristics of the coursework’s contents. For a fixed 3 to 4-year DrPH program, schools focused on fostering leadership skills. For other schools where the DrPH program was based on a department or a hybrid, coursework generally consisted of DrPH core requirements, departmental requirements, and departmental electives.

*Five MPH core courses waiver and transfer credit*. Abiding by MPH-level prerequisite courses or their equivalent, as required by the CEPH criteria [22], 11 DrPH programs require prerequisite coursework—five MPH core courses: Health Policy and Management, Environment Health Sciences, Health and Social Behavior, Statistics, and Epidemiology—but those credits can be waived for the admitted applicant who has earned an MPH degree. Schools require students without an MPH/MSPH degree to take either all five courses or one foundational course. In addition to those five MPH-level prerequisites, a total of 19 out of 28 DrPH programs (68%) allow transfer credits, from 6 to 32 credits, that have been completed at a CEPH-accredited or regionally accredited college or university. All schools that accept transfer credits require that students earn a minimum grade (B-, B, or higher) in the transfer courses.

**Fig 11:**
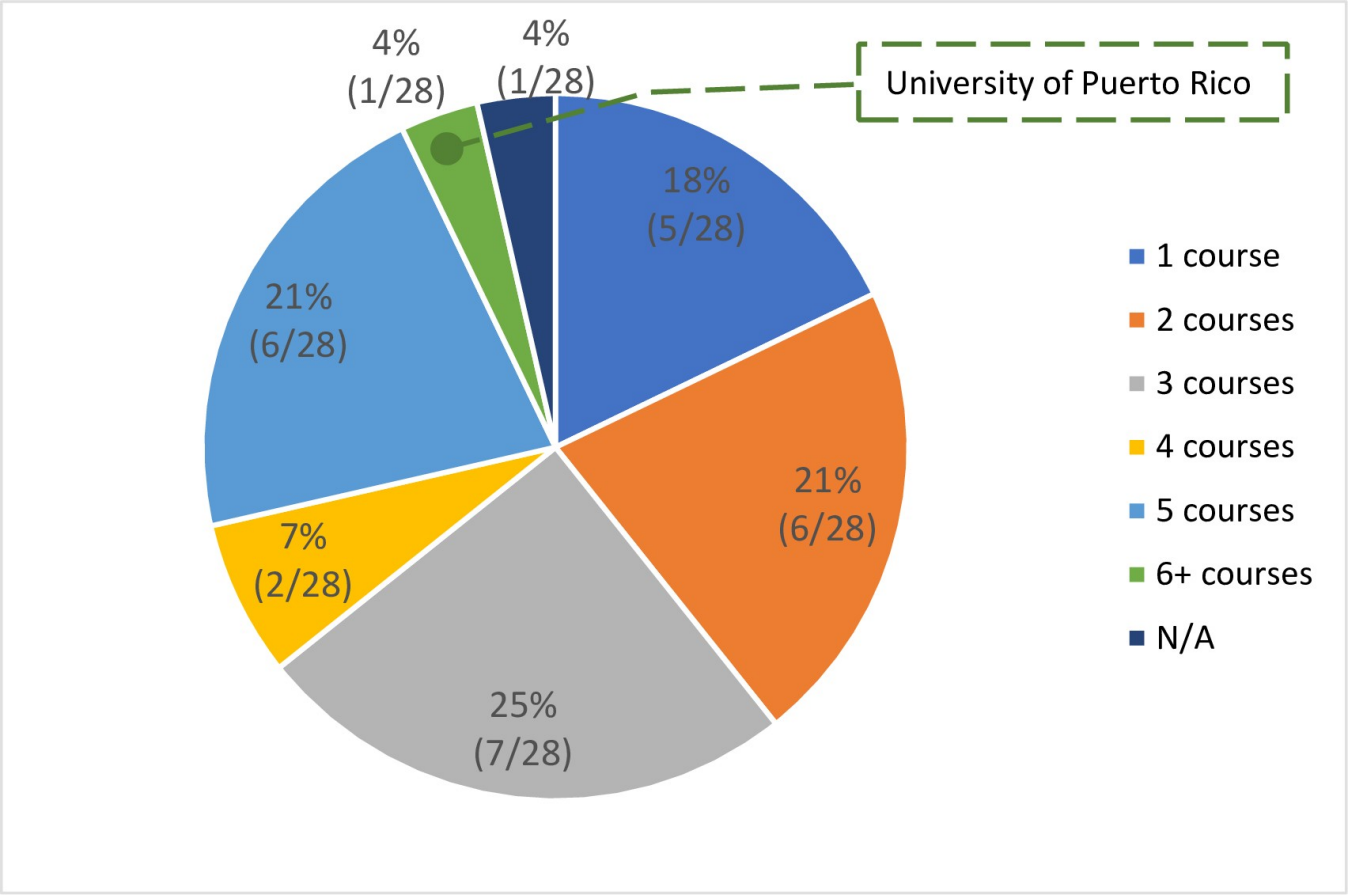
The number of leadership courses within the program.

All DrPH programs have established leadership-related courses as a required element of public health education within their school or program capacity or through collaboration with the business school. More than half of the DrPH programs (15/28) provide 3–5 leadership courses (Fig 11).

**Fig 12:**
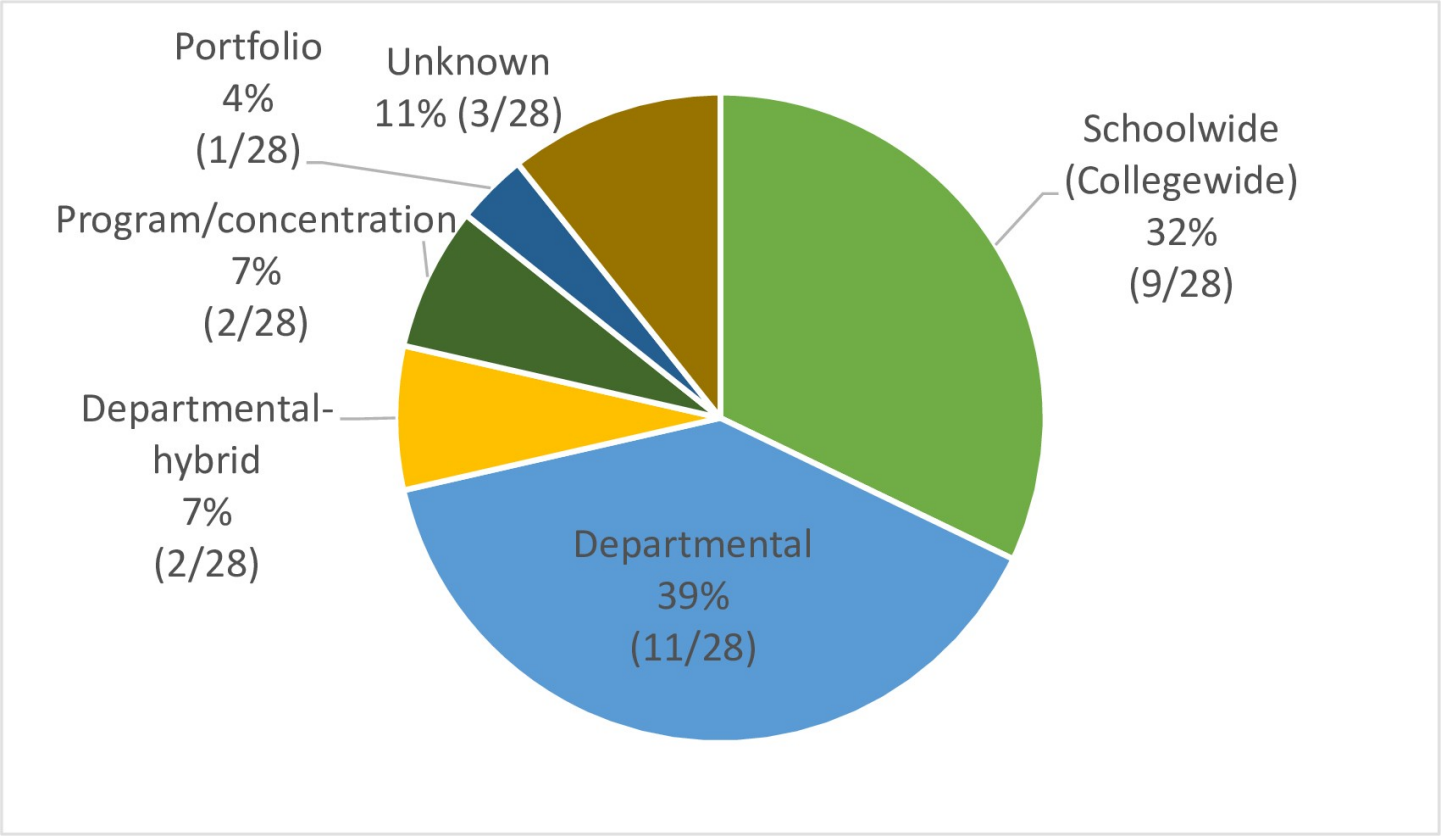
Types of qualifying/comprehensive exam.

*Qualifying/comprehensive exam*. Almost all DrPH programs require a qualifying/comprehensive exam, either take-home or closed-book. Only one school, the University of Illinois at Chicago School of Public Health, has adopted a different format, the portfolio—an integrative document of the student’s professional and academic experiences—to replace the usual doctoral preliminary and qualifying exams [35]. Fig 12 demonstrates the types of comprehensive exams. If departmental, Departmental-hybrid, and program/concentration are grouped one, 54% (15/28) of DrPH programs pursue specialty-specific comprehensive exams.

#### DrPH program practice and integrative learning experience

*DrPH applied practice experience*. The CEPH’s accreditation criteria, amended in October 2016, state that the completion of DrPH applied practice experience is required for all DrPH students “regardless of the amount or level of prior experience” [22]. This experience should demonstrate “advanced public health practice” and “a depth of competence” through a single project or multiple related projects [22]. Reference to DrPH applied practice experience differs across schools: as a practicum, advanced integrative practicum, high-level fieldwork, practical experience, field placement, field preceptorship, field immersion/DELTA, doctoral project internship, advanced field experience, consulting practicum, residency, executive management practicum, and professional practice.

Overall, each school subjectively interprets the criteria about the length of DrPH applied practice experience. It is not always clear how to objectively measure the following criterion: “substantive, quality opportunities that address the identified competencies.” As stated previously, more than half of schools (15 out of 28) require prior work experience in the field of public health to apply for a DrPH program; it is not clear how to ensure that DrPH applied practice experience can be a useful learning opportunity for those who have already worked substantially in public health.

*DrPH integrative learning experience*. For the integrative learning experience, almost all DrPH programs followed the traditional process of dissertation evaluation: dissertation proposal, dissertation proposal oral defense, and final dissertation oral defense. Students can choose either a traditional dissertation or a two to three paper option. The Harvard T. H. Chan School of Public Health program replaces the traditional dissertation evaluation process with DELTA progress reports, an oral final examination, and DELTA doctoral project deliverables [36].

#### DrPH internal funding opportunities

Since the DrPH is an advanced professional degree, most schools expect students to be responsible for working either inside or outside of the school on their own for self-funding.

Many schools state that graduate school funding can generally be provided in the form of graduate assistantships, graduate teaching assistantships, graduate research assistantships, fellowships, and various types of university- or school-wide scholarships. However, it is not clear whether those financial supports are also available to DrPH students.

#### DrPH graduate outcomes

**Fig 13:**
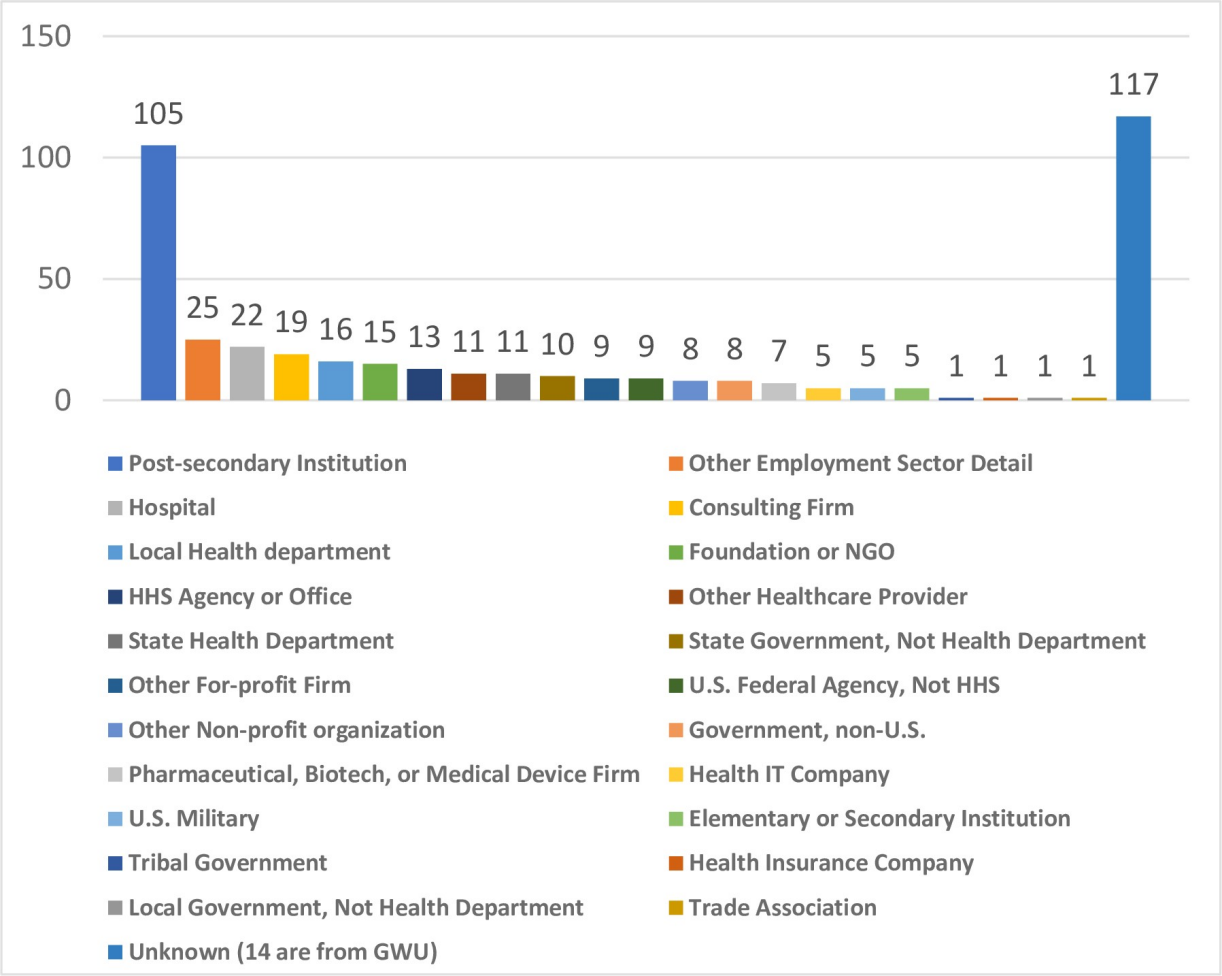
DrPH graduate outcome (2016–2018, grand total: 424).

The ASPPH Data Center Portal provided DrPH graduate outcomes in 2016, 2017, and 2018. The grand total of reported DrPH graduate outcome in the recent three years (2016–2018) was 424. The career pathways among DrPH graduates varied, from post-secondary institution to trade association (Fig 13).

**Fig 14:**
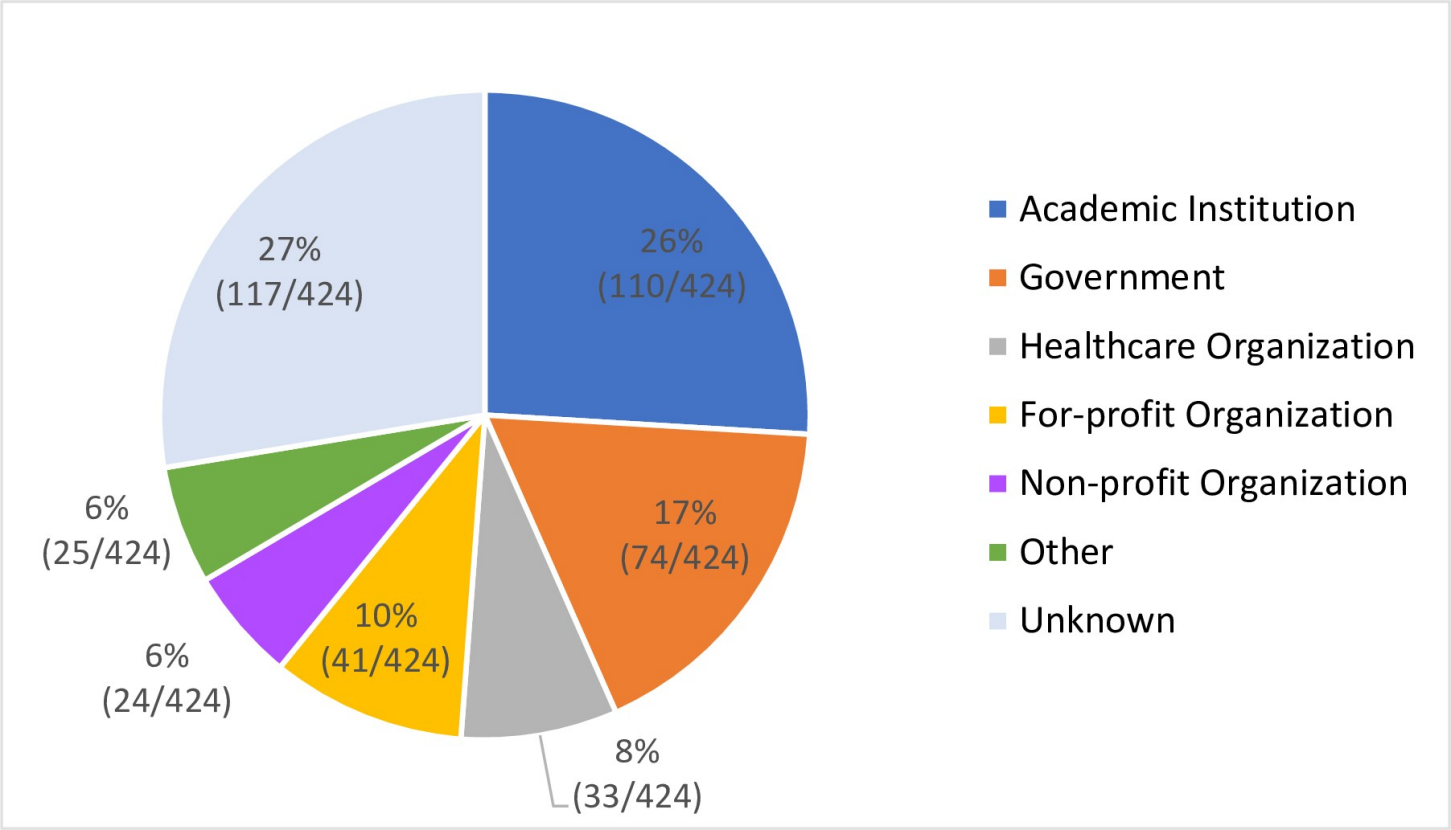
Distribution of graduation outcomes by employment sector.

As demonstrated in Fig 14, the academic institution sector took up the largest portion of the pie chart. 26% of DrPH alumni (110 out of 424 reported cases) entered academic institutions, mostly post-secondary institutions (95%, 105 out of 110). The second largest portion was government sector (17%, 74 out of 424 reported cases). In this sector, more than half of them went into local or state health departments, or the U.S. Department of Health and Human Services (54%, 40 out of 74), and a quarter of them went to local, state, or federal government positions that are not in a health department (27%, 20 out of 74).

### Qualitative analysis: In-depth interviews with DrPH directors

Results of the qualitative analysis were organized into the following three domains: 1) evolution of DrPH programs, 2) DrPH program principles and values, and 3) current status of DrPH programs. Quotations from verbatim transcriptions were left largely unaltered to reproduce exactly the way participants spoke. In addition, the names of the location, program, and institution were underscored to preserve participants’ confidentiality.

#### Evolution of DrPH programs

DrPH Programs have undergone many changes over the years, and they are still evolving. To gain insight into this evolution, participants were asked in how their DrPH program has evolved. The comments from participants fell into three main categories, in order of frequency: curriculum (85%, 17/20), program structure (50%, 10/20), and recruitment (15%, 3/20).

*Curriculum*. The evolution of the curriculum was sorted into the following six components: CEPH requirements, leadership, pedagogy, research methods, comprehensive exam, applied practice, and integrative learning experience.

*Curriculum–CEPH requirements*. DrPH programs have been evolving with the development of CEPH requirements. CEPH requirements have helped make the DrPH programs much more distinct from the PhD programs than they were in the past. For example, all DrPH programs have begun adopting the pedagogy requirements of CEPH by requiring students to teach two classes. Also, DrPH programs have become less focused on rigorous research and academics, favoring instead the development of leadership skills for training public health professionals to lead community-based participatory research:

*I think [CEPH requirements] really help distinguish the DrPH from the PhD a little bit more*, *so that the DrPH is less seen as a research and academic route*, *more for public health professionals who are going to work in leadership positions in a community setting*…. *I think it’s also just brought some additional leadership skills to our students*…. *We were already doing some classes on community-based participatory research. (Participant #21)*

*Curriculum–leadership*, *management & governance*. Leadership, Management & Governance is one of the DrPH foundational competencies in the CEPH criteria [22], and it must be emphasized when training students to become the future leaders of public health. Participants were asked how their DrPH program has focused on leadership courses and how those courses have been developed thus far. Those courses have evolved through the review of leadership courses from other DrPH programs or through alignment with CEPH competencies and their learning objectives:

*In developing that [leadership] course, I did review leadership courses from other DrPH programs*. *Also, we do have some faculty members there who have devoted their whole careers to studying leadership*.... *I would say that it’s kind of theory based, but there are practice-based experiences infused with the course that give them an opportunity to be out in the community during this course*, *and looking at how others are applying various approaches. (Participant #11)*

*Curriculum–pedagogy*. One of the major changes has involved including pedagogy requirements in the DrPH curricula of all DrPH programs based on the new CEPH competencies. Students are expected to acquire pedagogical experiences to be effective in educational roles, regardless of where their future takes them:

*We have purposefully added assignments to core courses that get students thinking about pedagogy*. *Whether they’re going to go into academia or whatever sector they choose, they’re probably going to have to be in some kind of educational role, whether it’s with a staff or a population-based group*. *I think we have a strong pedagogical influence in the leadership course. (Participant #11)*

*Curriculum–research methods*. Four participants described how their DrPH programs have been revamped to focus on research methods. Although the DrPH program focuses additionally on leadership and interdisciplinarity, developing research skills remains important because students are expected to conduct health services research, survey research, needs assessment, and gap analysis––activities essential to the field research. DrPH programs do not expect students to become a scientist or researcher; however, students are expected to acquire both quantitative and qualitative skills to develop a research design for, conduct, and complete a research project relevant to their field:

*We’ve been trying to get the balance correct between content and methods and… we don’t expect them to be biostatisticians*, *but we do expect them to have a fair amount of exposure to quantitative methods*. *(Participant #13)*

*Curriculum–comprehensive exam*. Four participants mentioned that a format and process for the DrPH comprehensive exam (qualifying exam) has been developed. While almost all DrPH programs require an oral and written exam, or a written exam, Participant #18’s DrPH program requires students to pass a unique comprehensive exam process called a portfolio:

*Even if you pass all of [qualifying exams], [it] doesn’t necessarily make you confident to lead change*.... *Can they demonstrate examples of how they can analyze quantitative, qualitative, and economic data? Can they apply the different kinds of research designs? They have to demonstrate all those pieces*.... *Anyone can go focus on a narrow area, but our program is more about “do you know how to do the work?” (Participant #18)*

*Curriculum–applied practice*. According to the CEPH criteria, significant advanced-level applied practice (practicum) can be completed from a student’s own work setting [22]. Among those who shared how the DrPH practicum functions for students working full-time, some participants (31%, 4/13) said the main topic of practicum should be completely different from the students’ current job aligning with the CEPH requirement. But the majority of participants (69%, 9/13) showed flexibility by allowing students to conduct a special project at their current job that was beyond the capacity of their normally required current duties. To complete the practicum requirement, students could set certain hours per week within a full-time work schedule without disrupting their career trajectory:

*They can complete it from their current job as long as they’re not doing the same thing as their regular job*. *If they’re doing a special project for their organization, they have to identify something new that is not part of their ongoing work requirement, and then they can do it in their job setting*. *Most of them do. (Participant #19)*

*Curriculum–integrative learning experience*. The last step of the DrPH curriculum is the integrative learning experience [22]. Students are expected to produce a high-quality field-based written product, such as dissertation, deliverable or report, which is related to their work from applied practice experience. Thus, the practicum and the dissertation are continuous steps that require students to demonstrate the ability to synthesize their advanced practice experience into a doctoral-level written product:

*We have modified our practicum and dissertation in the last couple of years to be more tailored to the CEPH requirement*.... *Most of [students] are already working with an organization or have an idea of which type of organization they want to work with… [or] they can work with their faculty advisor to identify potential ways of organizing their dissertation. (Participant #15)*

*Program formats*. A total of ten participants mentioned that the program format of their DrPH programs had developed in the following five areas: i) transforming from residential to online, ii) transforming from departmental-based to schoolwide, iii) transforming from research-focused to leadership-focused, iv) strengthening interdisciplinarity, and v) establishing a public health program.

Fifty percent of the participants (5/10) highlighted that interdisciplinarity has been strengthened by emphasizing the professional, leadership-geared aspect of the degree during the fixed-year term. Students have become much more exposed to interdisciplinary, interdepartmental, and independent research and the leadership environment:

*It’s evolved in two ways. We have tried to emphasize, more and more, the professional aspect of the programs… because it was once comparable to a PhD and now it’s considered a four-year program*.... *The other aspect is the interdisciplinarity of the program, now being stressed even more*. *We expect the students to really participate in multiple, interdisciplinary, interdepartmental, independent study development aspects of the program. (Participant #6)*

*Recruitment*. According to the quantitative analysis of DrPH programs, more than half of DrPH programs (54%, 15/28) require prior work experience. Among those programs, the required minimum years of work experience varies from two to five years to an unspecified number of years that should be sufficient to apply for the executive program (although not publicly stated, this number is expected to be 10-plus years). In-depth interviews regarding recruitment supported these quantitative results:

*We work hard to screen for people who are truly interested in asking questions… who have a focus on applied research, not a narrow knowledge, [but] to build knowledge and generate focus they must have at least three years of experience*.... *We’re talking about people who are mature emotionally and professionally, coming into the program with a shared desire and shared vision*.... *This cohort model [is] vital, so they become a learning community together and learn to work together. (Participant #18)*

#### DrPH program principles and values

The evolution of most DrPH programs follows a particular guiding principle or value. Interview participants did not receive a specific prompt pertaining to this topic. Rather, this question was left open to address any aspects that they felt were most important for the development of their DrPH programs from the past to the present. Most participants provided multiple answers; these principles or values were classified into the following four domains in order of the frequency with which they were mentioned: program direction (55%, 11/20), focus (specialty) (35%, 7/20), reports and guidelines (35%, 7/20), and program structure (10%, 2/20). Table 6 shows these results.

**Table 6:** DrPH program principles and values.

Category	Principles and values	Proportion
Domain 1: Program direction (55%, 11/20)	a) Missions, vision, and value, including goals and objectives	30%, 6/20
	b) Pedagogy	10%, 2/20
	c) Community partners	10%, 2/20
	d) Applied practice	10%, 2/20
	e) Diversity and inclusion	5%, 1/20
	f) Ethical decision making	5%, 1/20
	g) Training the best leader in public health	5%, 1/20
Domain 2: Focus (specialty) (35%, 7/20)	a) Leadership, management, communication, and innovation	30%, 6/20
	b) Health equity and social justice	15%, 3/20
	c) Research	5%, 1/20
	d) Social determinants of health	5%, 1/20
Domain 3: Reports and guidelines (35%, 7/20)	a) CEPH criteria	15%, 3/20
	b) Student handbook	15%, 3/20
	c) IOM report	5%, 1/20
Domain 4: Program structure (10%, 2/20)	a) Interdisciplinary	5%, 1/20
	b) Connection with organizations (transdisciplinarity)	5%, 1/20

*Program direction*. A total of 16 participants’ answers were categorized into seven different principles or values in Domain 1: Program direction (Table 6).

*Focus (specialty)*. Each of the DrPH programs focused on one overarching program direction; a total of 11 participants mainly mentioned leadership, research, health equity, social justice, or social determinant of health (Table 6).

*Reports and guidelines*. A total of seven participants highlighted that CEPH criteria (three participants), the student handbook (three participants), or the Institute of Medicine (IOM) report (one participant) were important sources for developing their DrPH programs (Table 6).

*Program structure*. Participants emphasized the unique character of the DrPH program structure: interdisciplinarity and furthermore, transdisciplinarity through a strong connection with diverse organizations (Table 6). In addition, bridging the gap between research (academia) and its application (practice) is the essential identity of the DrPH program [22]. To make this identity happen, both participants mentioned the importance of DrPH students developing leadership and management skills. Interdisciplinarity needs strong leadership to connect with real-world settings:

*Because of my situation in [region name], we try to be cognizant of reaching the populations there*. *And then I would say in general, we just really try to strengthen the leadership and management concentration so that we know that students are addressing those competencies*. *(Participant #8)*

#### Current status of DrPH programs

To have a better understanding of the current status of each of the DrPH programs, participants were asked about the programs’ strengths and weaknesses. Their answers were organized into six predominant positive categories and six predominant negative categories.

*Predominant positive categories*. Participants were asked about the strengths of their DrPH program and how these strengths successfully educate and empower future leaders of public health. Most participants provided multiple answers, grouped into the following six positive categories in order from highest percentage to lowest percentage: program structure and interdisciplinarity, curriculum–priorities and core expertise, faculty capacity, school location, partnership and application, and school reputation (Table 7).

**Table 7:** Predominant positive categories across DrPH programs.

Positive categories	Details
Category 1: Program structure and interdisciplinarity (100%, 20/20)	a) School (college)/school-wide (college-wide)
	b) School (college)/departmental
	c) School (college)/departmental-hybrid
	d) Program/concentration or track
Category 2: Curriculum—priorities and core expertise (90%, 18/20)	a) Embracing diverse students and accommodating part-time students
	b) Program specific
Category 3: Faculty capacity (55%, 11/20)	a) Mentoring
	b) Diverse disciplines
	c) Hard money
Category 4: School location (45%, 9/20)	a) Geographically easy to access to public health work
Category 5: Partnership and application (40%, 8/20)	a) Link to the community of practice
Category 6: School reputation (10%, 2/20)	a) School reputation to attract competitive students

Participants from each of the four program structures were interviewed. By and large, schoolwide programs train students to become generalists:

*Our model is for people who have had significant leadership experience because we believe that they can teach each other so much*. *We certainly have faculty who are experts but we also try to involve the students and bring their expertise into the classes as well. (Participant #19)*

Departmental-based and departmental-hybrid programs typically expect students to gain research skills along with a fair amount of leadership skills to become researcher-practitioners:

*While the PhD students focus on research-driven questions, our DrPH students can also focus on research-driven questions*, *but their focus is more on developing leadership and competencies*. *They bring advanced practice skill sets, and then they integrate with the students focused more on research*. *But they start to see the value of practice and research together. (Participant #15)*

Many programs embrace diverse students and accommodate part-time students. To equalize students’ level of knowledge for public health and embrace their diverse backgrounds, schools have made an effort to ensure the following activities: adhering to CEPH requirements and 20 DrPH foundational competencies, encouraging students to contact the schools before applying to discuss the nature of the program, reviewing all components of the student application holistically, having admitted students take prerequisite and core courses, and collecting student feedback:

*As we started the program and as we continue to refine it over time, and [we] collect student feedback*… *we’ve tried to be flexible, and some students really like that. Other students may not like that as much*. *They want more requirements and standards. And I think that we’ve been very responsive. (Participant #8)*

Strong faculty capacity—disciplinary diversity and mentoring—is another main strength of more than half of DrPH programs. Some schools have a large number of faculty from many different departments. Many schools also ensure the provision of one-to-one mentorship by assigning each student to a faculty advisor:

*Every student is assigned a faculty advisor when they come in, for one-to-one mentorship. They have that advisor throughout their stay in the program*, *but they also choose a chair for their qualifying exam and a chair for their dissertation committee; the chairs are in charge of content*. *(Participant #6)*

Almost half the participants mentioned a strong connection to the public health network close to the school. School location would therefore be one of the greatest strengths that might attract competitive students who want to have access to various public health work opportunities:

*I think one of the strengths of [school name] is the location is definitely a draw in terms of bringing Doctor of Public Health students to [school name] based upon our location [city name]*. *Many of our DrPH students are employed full-time in [city name], and they attend our program, which is another convenience; our program can be full-time or part-time*. *(Participant #1)*

By taking advantage of their locations, many schools and programs have created strong partnerships and collaborative relationships with communities, governmental organizations, NGOs, and any other CBOs geographically near to them. Their powerful links to communities can help students connect with their partners and build up hands-on experience through internships or practicums:

*We have a very strong belief in participation, so that even before they graduate, [students] have made links with the community of practice. So they do an internship between the first and second years that is more practical*.... *They don’t have experience of actual practice, but they have the opportunity, before diving into their dissertation, to select another area they haven’t worked in before to get more hands-on experience*. *(Participant #4)*

Finally, participants mentioned that their school’s high reputation and long history make up one of its biggest strengths. Those schools have produced a large number of successful alumni who still connect with students to collaborate with and support them.

*Predominant negative categories*. Next, participants answered the question about challenges they face in their DrPH programs. Participants’ answers were classified into the following six negative categories: cost of education, misunderstanding of DrPH degree, lack of faculty attention to mentoring, the difficulty of identifying ideal learners, unestablished program, and opportunity costs (Table 8).

**Table 8:** Predominant negative categories across DrPH programs.

Negative categories	Details
Category 1: Cost of education (70%, 14/20)	a) Lack of financial support
	b) High tuition cost
	c) High cost of living in a big city
Category 2: Misunderstanding of DrPH degree (30%, 6/20)	a) Unclear distinction between DrPH and PhD
	b) Mere extension of MPH
Category 3: Lack of faculty attention to mentoring (30%, 6/20)	a) Heavy burden on faculty (or lack of ownership)
	b) Incorrect guidance from PhD degreed faculty
Category 4: Difficulty of identifying ideal learners (25%, 5/20)	a) Selecting a consistent set of students
	b) Customizing curriculums for students with different backgrounds
Category 5: Unestablished program (20%, 4/20)	a) Testing all possible options for finding the best curriculum
	b) Dealing with new core competencies defined by CEPH
	c) Administratively separated
Category 6: Opportunity cost (5%, 1/20)	a) Unbalanced life

The majority of participants were concerned about the high cost of a DrPH education, including a lack of financial support, high tuition, and the high cost of living in a big city. Because the DrPH is a professional degree, it has no access to various types of grant funding; thus, financial aid opportunities for DrPH students are far fewer than those for PhD students. When attracting competitive students, even prestigious public schools have to compete with private universities that secure much better funding resources. However, those private universities are experiencing another challenge, given that tuition cost is expensive:

*The cost of living in the [region name] is one of the highest in the country. Housing is extremely expensive for our students*.... *Because it is a public university, we have limited financial aid that we can offer students*. *Especially because the DrPH is a professional degree, not an academic degree, we do not have access to grant money to support the students like PhD students have*.... *We are competing with private universities that often have much better financial aid packages than we can possibly offer. (Participant #6)*

Six participants cited a continued misunderstanding of what the DrPH degree is. Many of the PhD degreed faculty who lean toward an academic perspective still do not themselves have a deep understanding of the DrPH program nor a clear idea of how to guide DrPH students. Those in leadership positions at the university level do not understand the necessity of hiring faculty with nontraditional resumes for the DrPH program, which has given rise to hiring adjunct or part-time faculty. In addition, there is still an unclear distinction between the DrPH and PhD degrees. Moreover, some people share the misunderstanding that a DrPH degree is a mere extension of an MPH degree:

*There are some in the academic world that see the DrPH just as an extension of the MPH*… *Some would think the DrPH was a non-research degree. We don’t agree with that*.... *We [pursue] the kind of science that contributes to applied sciences outside the traditional paradigms clinical public health lives in. (Participant #18)*

Six participants were concerned about the lack of faculty mentoring of DrPH students. In the small DrPH program within the medical school, matching students with a particular mentor is challenging because mentors become saturated. For schoolwide DrPH programs, departments have no ownership of DrPH students. The resulting consequence is that the progress of the students’ dissertations may rely on faculty good will, which must be very challenging for students:

*If you have a student who’s struggling or someone who needs an additional person on their dissertation committee, a department chair can be very influential in encouraging faculty to participate at that level if they feel they have ownership over the program*. *That’s much more complicated for us to manage as a school-wide program, because I have neither carrots nor sticks for faculty besides just genuine good nature and a truly good experience working with our students. (Participant #17)*

Identifying ideal learners for DrPH programs is another challenge. Some participants mentioned that selecting a consistent set of students is challenging, and others felt it was challenging to customize curriculums for students with different backgrounds:

*We’ve seen that in the past three years we’ve attracted a lot of medical doctors, who make wonderful students, but then we have some others that are at a little bit of a different stage, both from a programmatic and a maturity level*. *That’s a challenge sometimes, to make sure the cohort that we select has a consistent mind-set. (Participant #13)*

*Some students who apply to our program are interested in earning a degree, and they’ve been in the field for 15, 20 years, very skilled professionally*, *but haven’t been in an academic setting in a while. [It] can be tricky, and we need to customize things for these different types of students. (Participant #15)*

Four participants mentioned that the establishment of the DrPH program is still in progress. While transitioning from one program structure to the other, revising the curriculum to adopt new CEPH competencies, or launching a new DrPH program, participants have been experiencing trials and errors in determining the best way to deliver the curriculum to students and keep it aligned with CEPH requirements:

*We completely revamped our DrPH program, and as a result, we still have not finished a cohort yet. Now, so far, it’s working pretty well*, *but it would be nice to be able to see one full cohort go through before we can call it a success*. *I guess one challenge might be that we’re still sort of tweaking the process as we’re going through it. (Participant #20)*

Finally, due to the difficulty of balancing their lives, students may struggle with graduating within a reasonable time frame. Faculty are experiencing an operational challenge in getting students out of the program within a reasonable timeline:

*How do you balance the need to have students do rigorous research with the knowledge that they've got demanding lives? One challenge is for us to make sure they’re getting the content curriculum*, *and experience they need for the program. But at the same time, getting them out of the program in a reasonable amount of time is another operational challenge. (Participant #18)*

## Discussion

It was worthwhile to conduct this study because the effort to find an optimal education strategy has not been deeply discussed among schools since the effort to standardize public health training in 1910. Catalyzed by the Welch-Rose Report in 1915, there have been many attempts to develop DrPH programs. However, those efforts have not been systematically corroborated to develop the identity and brand of a DrPH degree; as of March 2020, each school or program provides a different structure for the DrPH degree and has varied program lengths—from 3 years to 10 years—due to varying expectations and interpretations of this terminal degree in public health.

Establishing a strong common sense of DrPH identity would attract promising future leaders in public health who would want to choose a DrPH degree over a PhD degree. Once a general expectation for a DrPH degree is established, employers from various sectors would understand what kinds of common skill sets and abilities can be normally acquired by DrPH candidates. A DrPH degree has been continually suspended, removed, or created across schools, colleges, or universities in the United States even during recent years, presumably due to a lack of common understanding of its identify; if there were a more consistent format to a DrPH degree, academic institutions could expect to avoid barriers in designing a DrPH curriculum and differentiate it from a PhD curriculum––and then improve the DrPH degree. Establishing generally accepted US DrPH structures and curriculum models would provide a good example for other countries that wish to launch the DrPH degree and do benchmarking and strategic planning. Thus, establishing common themes and variations of DrPH programs in the United States will be eventually beneficial to academic institutions in locations that plan to further strengthen or newly establish such a degree.

There was a limitation to identify the entire CEPH-accredited DrPH programs across the country. The ASPPH Program Finder—the webpage used for finalizing the list of CEPH-accredited DrPH programs—was not a perfect tool, even if it is widely known as the most trustworthy database to search for any of the CEPH-accredited public health degrees. Manual searching was subsequently conducted to identify additional DrPH programs. After cross-checking between the CEPH webpage [28] and a manual search, six additional DrPH programs arose that were not searchable in the ASPPH Program Finder: East Carolina University, Florida A&M University, Indiana University Richard M. Fairbanks School of Public Health, Jackson State University School of Public Health, Morgan State University School of Community Health and Policy, and Ponce Health Sciences University. However, those manually identified DrPH programs were excluded in the final list because it was unknown whether they had successfully received DrPH-specific CEPH accreditation from the publicly available data, and the CEPH webpage only listed accredited schools and programs without being degree-specific [28]. To avoid the possibility of missing any other CEPH-accredited DrPH programs from the manual search, the final list of CEPH-accredited DrPH programs resulted from the most conservative method: the ASPPH Program Finder. Reflecting on the final list, the interview transcript from Indiana University Richard M. Fairbanks School of Public Health was excluded from the qualitative analysis. To include all CEPH-accredited DrPH programs across the country for future research, it is expected that websites and publicly available data from both ASPPH and CEPH should provide the up-to-date, exact information.

In a methodological sense, it is recommended that future studies of this nature include opinions from groups other than program directors such as students, applicants, and alumni to gain an understanding of different views and perceptions of the DrPH degree and training. In addition, interviews with CEPH officials would provide their opinions and expectations for the DrPH degree including a clearer concept of the distinction between DrPH and PhD programs. From the quantitative point of view, more people are entering the field of public health, either to acquire skills or advance their skills to take on the highly complex public health challenges of the day. From the qualitative point of view, DrPH programs are not only training these professionals to acknowledge core competencies but also striving to maintain the academic freedom, build unique curriculums, and meet specific areas of public health. The core competencies of the DrPH will prepare the candidate for the application of knowledge in addition to the generation of knowledge resulting in a uniquely well rounded and prepared professional who leads critical public health programs of the future.

## Conclusions

In the United States, the effort to confirm the clear identity of the DrPH education has not been completed in over 100 years. How should DrPH program train a well-balanced, educated public health workforce that established both academic and practical skills? Should each academic institution interpret adopt the DrPH program in the same way? Here are a few recommendations for a further development of DrPH education. First, DrPH education should focus on applied social science to promptly address complex humanitarian emergencies in real life. Second, a DrPH degree should increase the brand awareness as a practice-based terminal degree and establish a unique identity and its purpose, which should be distinct from a PhD degree. Third, beyond satisfying the CEPH criteria of leadership and governance, the DrPH program should be designed to train students practically to help them gain a higher level of leadership skills that are highly applicable on the ground. Fourth, a DrPH degree should train a generalist or a researcher-practitioner who mainly focuses on practice and application of various aspect of public health with a deep knowledge of research. Fifth, CEPH should provide clearer curriculum guidance on how to differentiate a DrPH degree from a PhD degree so that all faculty from DrPH programs have a better sense of mentoring DrPH students in the right direction. Last but not least, DrPH curriculum should be further standardized across the country, so everyone can understand a clear, overarching goal of a DrPH degree.

## Supporting information

S1 Table List of DrPH CEPH-accredited programs in the United States. (PDF)

S2 Table Prerequisites and application requirements. (PDF)

S3 Table Structure of 28 CEPH-accredited DrPH schools and programs. (PDF)

S4 Table Transfer credit and coursework. (PDF)

S5 Table Comprehensive/qualifying exam. (PDF)

S6 Table Applied practice experience and integrative learning experience. (PDF)

S7 Table Required leadership and finance courses. (PDF)

S8 Table Internal funding information. (PDF)

## Abbreviations

APHA: American Public Health Association
ASPH: Association of Schools of Public Health
ASPPH: Association of Schools and Programs of Public Health
CEPH: Council on Education for Public Health
DrPH: Doctor of Public Health
IOM: Institute of Medicine
RSC: Rockefeller Sanitary Commission
