# Supplementary material for: Analysis of CEPH-accredited DrPH programs in the United States: A mixed-methods study
Source: PLoS One. 2021 Feb 4;16(2):e0245892. doi: 10.1371/journal.pone.0245892 (PMC7861440; doi:10.1371/journal.pone.0245892)
Supplement: S2 Table — (PDF) [file pone.0245892.s002.pdf]

S2 Table. Prerequisites and application requirements

School Name	MPH degree	Work experience	Application requirements
1. Boston University School of Public Health [1-3]	MPH or other master's degree in a field of study pertinent to public health is needed.	Three or more years of full-time public health or relevant experience.	No GRE (from Fall 2020); official transcripts; three letters of recommendation; statement of purpose and objective; CV or resumé; SOPHAS supplemental application
2. Claremont Graduate University School of Community & Global Health [4-6]	MPH, related U.S. equivalent master's degree(s), or terminal clinical/doctoral degree from a regionally accredited college or university is needed.	Non-requirement	GRE; official transcripts; three letters of recommendation; statement of purpose; resumé
3. University of Colorado School of Public Health [7-10]	▪ It is recommended to have MPH or equivalent CEPH-accredited program suited for each department: Community Behavioral Health, Environmental and Occupational Health, and Epidemiology.<br>▪ If admitted without an equivalent degree, courses must be taken during the first year as co-requisites.<br>▪ Minimum GPA of 3.5/4.0 in previous graduate-level studies (If GPA is below 3.5, explanation of reason and planning to address prior academic deficiencies is needed)	At least two years of work in public health or a related field is preferred.	▪ GRE (optional); official transcripts; three letters of recommendation; personal statement; resumé or CV<br>▪ DrPH Program in Epidemiology: non-MPH required course (calculus) must be completed prior to admission.

School Name	MPH degree	Work experience	Application requirements
4. Columbia University Mailman School of Public Health [11-16]	It is required to have a MPH or its equivalent degree from CEPH-accredited program.	▪ DrPH Program in Leadership in Global Health and Humanitarian Systems: five years of post-graduate experience in global health, development, humanitarian work in a substantial management or leadership role	▪ General: GRE; official transcripts; three letters of recommendation; 500-word personal statement; CV or resumé<br>▪ DrPH Program in Leadership in Global Health and Humanitarian Systems: GPA of at least 3.5 in prior graduate study; 50th percentile or grater in the verbal and quantitative sections of GRE (GRE is exempted for applicants with professional doctoral degrees, such as MD, DDS, JD); MCAT scores can be substitution for GRE (25 for the pre-2015 MCAT and 500 for the post-2015 MCAT)
5. Drexel University Dornsife School of Public Health [17-19]	MPH or equivalent degree	▪ Minimum of 2 years of work experience in health-related field<br>▪ Ideally 5 years	GRE; official transcripts; three letters of recommendation; written statement of career goals; doctoral program interests; CV or resumé; writing sample
6. East Tennessee State University College of Public Health [20-25]	MPH or equivalent degree in a related field from a regionally accredited institution of higher learning in the United States	Non-requirement	GRE; official transcripts; three letters of recommendation; personal essay
7. George Washington University Milken Institute School of Public Health [26-30]	▪ Non-requirement<br>▪ MPH degree from CEPH accredited program is preferred.<br>▪ Another master's degree may be considered.<br>▪ Non-MPH graduates are required to take additional coursework.	Non-requirement	GRE; official transcripts; three letters of recommendation; 1,500 word personal statement; CV or resumé; interview

School Name	MPH degree	Work experience	Application requirements
8. Georgia Southern University Jiann-Ping Hsu College of Public Health [31-35]	▪ Should complete at least 1 graduate-level core course in each of the following 5 areas: biostatistics, epidemiology, social and behavioral sciences in public health, health policy and management, and environmental health.<br>▪ Exception: applicants who shows exceptional potential for success in the DrPH program—require to complete MPH core courses with grades of B or better before starting the DrPH program<br>▪ Minimum GPA of 3.0/4.0 in upper division graduate courses is preferred.	▪ Preferred<br>▪ If the applicant has completed another Master's degree, professional public health work experience in one or more of the five core areas may be substituted for the corresponding MPH level courses at the discretion of the College.	GRE; official transcripts; three letters of recommendation; 7000–1,000 word statement of purpose; CV or resumé
9. Georgia State University School of Public Health [36-38]	▪ Should possess at least a master's-level graduate degree<br>▪ MPH or equivalent degree is preferred.<br>▪ Master's degree other than public health may be considered on a case-by-case basis—those applicants accepted should take prerequisite MPH core curriculum courses.	Minimum of 3 years of verifiable, applied public health experience	GRE; official transcripts; three reference letters; statement of purpose and objectives; CV or resumé

School Name	MPH degree	Work experience	Application requirements
10. Harvard T.H. Chan School of Public Health [39-42]	A master's OR doctoral degree in the health sciences or in another related field or non-US equivalent.	▪ At least 6 years of full-time public health and/or public service experience in a relevant discipline.<br>▪ Advanced degrees will not be considered as work experience	GRE; official transcripts; three letters of recommendation; statement of purpose (answer for 3 questions, 500-word each); CV or resumé; interview
11. Johns Hopkins Bloomberg School of Public Health [43, 44]	▪ MPH or equivalent degree<br>▪ Student without MPH should complete additional coursework.	A minimum of 3 years of professional public health experience in the applicant's area of interest	GRE/GMAT (waivers may be granted in rare cases); official transcripts; three letters of recommendation; personal statement; CV or resumé; interview
12. Loma Linda University School of Public Health [45-50]	▪ MPH or master's degree in a related field<br>▪ Minimum of GPA of 3.5 of master's degree	Non-requirement	GRE; official transcripts; three letters of recommendation; written statement; interview
13. New York Medical College School of Health Sciences and Practice & Institute of Public Health [51]	▪ MPH in Health Policy and Management or a comparable degree with a minimum of GPA 3.5<br>▪ Accepted applicants without MPH need to complete coursework required for the MPH in Health Policy and Management.	Non-requirement	GRE; official transcripts; three letters of recommendation; 500-word personal statement; resume; public health prerequisite worksheet
14. Pennsylvania State University College of Medicine Public Health Program [52, 53]	▪ Non-requirement<br>▪ Student without MPH should take 15-credit prescribed MPH core courses before and/or during the DrPH program and pass a written exam.	Non-requirement	GRE; official transcripts; three letters of recommendation; 1,000-word statement of purpose; CV or resume

School Name	MPH degree	Work experience	Application requirements
15. SUNY Downstate Medical Center School of Public Health [54, 55]	▪ MPH and relevant master's degree<br>▪ The applicant should take additional course work if master's degree doesn't satisfy the core MPH curriculum.<br>▪ Minimum of GPA 3.5/4.0 in the major course of study for graduate academic record is preferred.	Non-requirement	GRE/MCAT/DAT/GMAT/LSAT/OAT/USMLE Step 1 or 2/NBVME (exempted if MPH with a GPA 3.0 or above); official transcripts; three letters of recommendation; 1,000 word personal statement; CV; personal interview; on-site interview
16. Texas A&M School of Public Health [56, 57]	Conferred master's degree	Non-requirement	GRE; official transcripts; three letters of recommendation; statement of purpose; CV or resumé; personal interview
17. Tulane University School of Public Health and Tropical Medicine [58]	▪ MPH or equivalent with an outstanding academic record<br>▪ A post-bachelor's degree GPA of 3.5 or higher is preferred.	Practical experience in public health is highly preferred.	GRE (preferred upper 50 percentiles); official transcripts; three letters of recommendation; career statements
18. University at Albany School of Public Health [59, 60]	▪ MPH or other master's degree in health, social science, or related field<br>▪ Student without MPH should take all MPH core courses	Normally at least post-master 2-year work experience in public health or related field	Official transcripts; three letters of recommendation; statement of purpose; CV or resumé; personal interview
19. University of Alabama at Birmingham School of Public Health [61, 62]	▪ Non-requirement<br>▪ DrPH in Health Care Organization & Policy: admitted applicant with MPH should complete 20–	Non-requirement	GRE (at least 70th percentile for both verbal and quantitative sections); official transcripts (at least 3.0/4.0 of a bachelor's degree); three letters of recommendation; statement of purpose and objectives; CV or resumé

School Name	MPH degree	Work experience	Application requirements
	23 credits of MPH core requirements.		
20. University of Arizona Mel and Enid Zuckerman College of Public Health [63-66]	▪ Should have Master's degree, Preferably MPH<br>▪ GPA of 3.2 or higher<br>▪ Students entering without an MPH must complete the MPH core courses in addition to degree requirements.	At least 2 years of public health experience	GRE; official transcripts (at least 3.0/4.0 of a bachelor's degree); three letters of recommendation; 500-word advanced practice statement (DrPH in Maternal and Child Health) or 500-word policy statement (DrPH in Public Health Policy); CV or resumé; writing sample; mission and values statement
21. University of Arkansas for Medical Sciences Fay W. Boozman College of Public Health [67]	▪ Candidates must have completed at least one graduate-level course with a grade of B or better in each of the following five areas: Biostatistics, Epidemiology, Health Behavior/Health Education, Health Management/Policy, and Environmental and Occupational Health.<br>▪ Exception: applicants who complete 4 of these courses and show exceptional potential for success in the program can be admitted conditionally.	Non-requirement	GRE; official transcripts; three letters of recommendation; 500-700 words of statement of experience, purpose, and objectives; CV or resumé; professional writing sample
22. University of California Berkeley School of Public Health [68, 69]	▪ MPH or Master's degree from a CEPH-accredited school	▪ Minimum of 2 years or more of professional public health experience post-master's degree	GRE/MCAT/DAT/OAT/GMAT/LSAT (exempted for MD, PHD, doctoral-level degree applicants, MBBS degree with passing Step 1 or 2 of the USMLE applicants);

School Name	MPH degree	Work experience	Application requirements
	▪ Prerequisite courses are required for applicants without MPH	▪ Some exceptions to the 2-year post-master's work requirement may be made in special circumstances.	official transcripts; three letters of recommendation; 1,500-word statement of purpose; 5,000-character personal history statement; CV or resumé; up to 7,000-word writing sample
23. University of Georgia College of Public Health [70, 71]	▪ Master's-level degree in public health (MPH or MSPH)<br>▪ Students with other master's degrees should complete the five MPH core courses (15 hours) prior to the DrPH program.<br>▪ Minimum GPA of 3.0/4.0<br>▪ Students with only a bachelor's degree will not be accepted. No exceptions.	At least 3-year post-master experience in the field of public health	Official transcripts; at least three letters of recommendation; statement of purpose and degree objectives; CV or resumé; personal interview
24. University of Illinois at Chicago School of Public Health [72-74]	▪ Master's degree in public health or related program<br>▪ Students without an MPH should take additional foundational, core MPH courses.	3 or more years of full-time, paid, professional experience in a public health leadership position, or in mid- to senior-level management positions that demonstrate leadership potential.	Official transcripts; at least three letters of recommendation; 500-800 words of personal statement; CV or resumé
25. University of North Carolina Gillings School of Global Public Health [75, 76]	▪ Master's or doctoral degree (not necessarily in public health)<br>▪ Students without a MPH or MSPH degree should complete one core course—Foundation Learning Objective online class	At least 5 years of post-graduate experience in the health field with significant management or leadership responsibilities	GRE/GMAT (combined score of 1,000 or 50th percentile or greater in both verbal and quantitative sections), MD, DO, DDS, JD applicants are exempted; Official transcripts; at least three letters of recommendation; single-spaced, no more than 5-page essay; resumé

School Name	MPH degree	Work experience	Application requirements
26. University of Puerto Rico Graduate School of Public Health [77-80]	Master's degree in public health and relevant degree with a minimum of 3.0/4.0	▪ DrPH in Health Systems Analysis and Management: have experience in teaching, research or service in the field of public health<br>▪ DrPH in Social Determinants of Health: have experience in teaching, research or service in the field of public health<br>▪ DrPH in Environmental Health: certifications of professional experiences of the last 5 years by the human resources department and the immediate supervisor of the companies or agencies	▪ General: Writing skills in Spanish and adequate understanding in English; GRE (average of 140 in both parts) or EXADEP (score of 500 or more); Official transcript(s) of graduate studies (master's or doctorate); three letters of recommendation; CV; interview<br>▪ DrPH in Health Systems Analysis and Management: (1) writing an essay through a word on the day of the oral interview; (2) complete a) undergraduate-level pre-calculus and graduate-level b) finances, c) statistical inference and d) economics with a minimum grade B; (3) computer program skills (MS word, MS Excel, MS PowerPoint) and at least one statistical program such as STATA, EPI-INO, SYSTAT<br>▪ DrPH in Social Determinants of Health: (1) 5-7 page essay; (2) performing other work (reading and presentation of professional article, or essay writing) on the day of the interview; (3) complete statistical inference course<br>▪ DrPH in Environmental Health: (1) writing an essay using Word on the day of the oral interview; (2) complete at least one graduate-level course, a) statistical inference, b) air pollution, c) aquatic environment, and d) environmental toxicology; (3) computer program skills (MS word, MS Excel, MS PowerPoint) and at least one statistical program such as STATA, EPI-INO, SYSTAT

School Name	MPH degree	Work experience	Application requirements
27. University of South Florida College of Public Health [81, 82]	MPH, MHA, or equivalent degree<br>Student with other graduate degree should complete all five public health core courses.	Minimum of 2 years of work experience in public health or closely related field or as a health professional	GRE; official transcripts; at least two letters of recommendation; statement of purpose; CV or resumé; statement of public health experience
28. University of Texas Health Science Center at Houston School of Public Health [83, 84]	MPH degree or equivalent preparation	No. Prior work experience is not necessary but helpful.	GRE; official transcripts; three letters of recommendation; essay/personal statement & objectives; CV or resumé; personal interview

References

1. Boston University School of Public Health: Doctor of Public Health (DrPH). https://www.bu.edu/sph/education/degrees-and-programs/doctor-of-public-health-drph/ (2019). Accessed August 12 2019.
2. Boston University School of Public Health: DrPH Program Guidelines 2018-2019. https://www.bu.edu/sph/files/2018/10/DrPH-Handbook-2018.pdf (2018). Accessed August 12 2019.
3. Boston University School of Public Health: Transfer credit or waiver policies. https://www.bu.edu/sph/students/advising-and-registration/policies-and-procedures/policies/transfer-credit-or-waiver-policies/ (2019). Accessed August 12 2019.
4. Claremont Graduate University School of Community & Global Health: Doctor of Public Health. https://www.cgu.edu/academics/program/doctor-public-health/ (2019). Accessed August 12 2019.
5. Claremont Graduate University School of Community & Global Health: Archived Bulletin - Public Health, DrPH. http://bulletin.cgu.edu/preview_program.php?catoid=13&poid=1646&returnto=1595 (2019). Accessed August 12 2019.
6. Claremont Graduate University School of Community & Global Health: Archived Bulletin - Registration & Enrollment. http://bulletin.cgu.edu/content.php?catoid=13&navoid=1511#Transfer (2019). Accessed August 12 2019.
7. Colorado School of Public Health: Doctor of Public Health - DrPH Programs at the Colorado School of Public Health. http://www.ucdenver.edu/academics/colleges/PublicHealth/Academics/degreesandprograms/Pages/DrPH.aspx (2019). Accessed August 12 2019.
8. Colorado School of Public Health: Doctor of Public Health 2019-2020 Student Handbook. http://www.ucdenver.edu/academics/colleges/PublicHealth/resourcesfor/currentstudents/academics/Documents/19_20_Handbooks/DRPH_Handbook_19_20.pdf (2019). Accessed September 30 2019.
9. Colorado School of Public Health: Practicum & Capstone. http://www.ucdenver.edu/academics/colleges/PublicHealth/resourcesfor/currentstudents/academics/Pages/PracticeBasedLearning.aspx (2019). Accessed August 12 2019.
10. Colorado School of Public Health: DrPH Requirements. http://www.ucdenver.edu/academics/colleges/PublicHealth/admissionsandaid/howtoapply/Pages/DrPHReqs.aspx (2019). Accessed August 12 2019.
11. Columbia University Mailman School of Public Health: Doctoral Guidelines - PhD and DrPH Programs in Epidemiology. https://www.mailman.columbia.edu/sites/default/files/pdf/doctoral_guidelines.pdf (2017). Accessed August 12 2019.
12. Columbia University Mailman School of Public Health: The Department of Biostatistics Student Handbook 2019-2020. https://www.mailman.columbia.edu/sites/default/files/biostats_student_handbook_2019-2020.pdf (2018). Accessed September 30 2019.
13. Columbia University Mailman School of Public Health: DrPH. https://www.mailman.columbia.edu/academics/degrees/degree-requirements/drph (2019). Accessed August 12 2019.

14. Columbia University Mailman School of Public Health: Department of Sociomedical Sciences Doctoral Student Handbook 2019-2020. https://www.mailman.columbia.edu/sites/default/files/pdf/sms-doctoral-handbook-2019-20.pdf (2019). Accessed September 30 2019.
15. Columbia University Mailman School of Public Health: Doctoral Student Handbook. https://www.mailman.columbia.edu/sites/default/files/pdf/doctoral-handbook_2.pdf (2018). Accessed August 12 2019.
16. Columbia University Mailman School of Public Health: Heilbrunn Department of Population & Family Health Doctoral Program Handbook Version 4.1 (March 2018). https://www.mailman.columbia.edu/sites/default/files/pdf/pfh-drph-handbook-march-2018.pdf (2019). Accessed January 8 2020.
17. Drexel University Dornsife School of Public Health. DSPH Student Handbook AY 2018 to 2019. 2018.
18. Drexel University Dornsife School of Public Health: DrPH in Health Management & Policy. https://drexel.edu/dornsife/academics/degrees/drph-in-health-management-and-policy/ (2019). Accessed August 12 2019.
19. Drexel University Dornsife School of Public Health: Health Management and Policy DrPH - About the Program. http://catalog.drexel.edu/graduate/schoolofpublichealth/healthmanagementandpolicydrph/#text (2019). Accessed August 12 2019.
20. East Tennessee State University College of Public Health: DrPH Field Experience Guidelines 2017-18. https://www.etsu.edu/cph/documents/drphfieldexperienceguidelines.pdf (2017). Accessed August 12 2019.
21. East Tennessee State University College of Public Health: Graduate Health Professions Education Doctor of Public Health 2019-2020 Student Handbook. https://www.etsu.edu/cph/documents/drphhandbook.pdf (2018). Accessed January 9 2020.
22. East Tennessee State University College of Public Health: Doctoral Programs. https://www.etsu.edu/cph/academics/doctoral.php#tab-7-1 (2019). Accessed August 12 2019.
23. East Tennessee State University College of Public Health: Archived Catalog - Public Health, Dr.P.H. (Community Health Concentration). https://catalog.etsu.edu/preview_program.php?catoid=27&poid=10427&returnto=1347 (2019). Accessed August 12 2019.
24. East Tennessee State University College of Public Health: Archived Catalog - Public Health, Dr.P.H. (Epidemiology Concentration). https://catalog.etsu.edu/preview_program.php?catoid=27&poid=10428&returnto=1347 (2019). Accessed August 12 2019.
25. East Tennessee State University College of Public Health: Archived Catalog - Public Health, Dr.P.H. (Health Management and Policy Concentration). https://catalog.etsu.edu/preview_program.php?catoid=27&poid=10502&returnto=1347 (2019). Accessed August 12 2019.
26. George Washington University Milken Institute School of Public Health: Program Guide - Doctor of Public Health, Environmental and Occupational Health. https://publichealth.gwu.edu/sites/default/files/DrPH%20EOH%202018%20%28May%202018%29.pdf (2018). Accessed August 12 2019.

27. George Washington University Milken Institute School of Public Health: Program Guide - Doctor of Public Health, Global Health. https://publichealth.gwu.edu/sites/default/files/DrPH%20Global%20Health%202018_0.pdf (2018). Accessed August 12 2019.
28. George Washington University Milken Institute School of Public Health: Program Guide - Doctor of Public Health, Health Behavior. https://publichealth.gwu.edu/sites/default/files/DrPH%20HB%202018_0.pdf (2018). Accessed August 12 2019.
29. George Washington University Milken Institute School of Public Health: Program Guide - Doctor of Public Health, Health Policy. https://publichealth.gwu.edu/sites/default/files/DrPH%20Health%20Policy%202018_0.pdf (2018). Accessed August 12 2019.
30. George Washington University Milken Institute School of Public Health: DrPH Programs. https://publichealth.gwu.edu/academics/graduate/drph-programs (2019). Accessed August 12 2019.
31. Georgia Southern University Jiann-Ping Hsu College of Public Health: Graduate Assistantship (GA) Handbook 2017-2018. https://docs.google.com/file/d/0B2ms15eoGveqMkJKS2J6U3pKSm8/edit?usp=embed_facebook (2017). Accessed August 12 2019.
32. Georgia Southern University Jiann-Ping Hsu College of Public Health: Student Handbook 2017-2018. https://drive.google.com/file/d/0B2ms15eoGveqOHlEUW53X0ZSWVE/view?usp=embed_facebook (2017). Accessed August 12 2019.
33. Georgia Southern University Jiann-Ping Hsu College of Public Health: Site Supervisor Handbook 2017-2018. https://drive.google.com/file/d/0B2ms15eoGveqdFNnTldkUlcyMVE/view?usp=embed_facebook (2017). Accessed August 12 2019.
34. Georgia Southern University Jiann-Ping Hsu College of Public Health: Degrees & Programs. https://jphcoph.georgiasouthern.edu/degrees/#DrPHDegree (2019). Accessed August 12 2019.
35. Georgia Southern University Jiann-Ping Hsu College of Public Health: Catalog 2019-2020 - Doctor of Public Health. https://catalog.georgiasouthern.edu/graduate/jiann-ping-hsu-public-health/doctor-public-health/ (2019). Accessed August 12 2019.
36. Georgia State University School of Public Health: Doctor of Public Health - School of Public Health. https://publichealth.gsu.edu/academics-student-life/degrees-programs/drph/ (2019). Accessed August 12 2019.
37. Georgia State University School of Public Health: Doctor of Public Health - Frequently Asked Questions. https://publichealth.gsu.edu/academics-student-life/degrees-programs/drph-faq/ (2019). Accessed August 12 2019.
38. Georgia State University School of Public Health: Doctor of Public Health - How to Apply. https://publichealth.gsu.edu/academics-student-life/degrees-programs/drph-apply/ (2019). Accessed August 12 2019.
39. Harvard T.H. Chan School of Public Health: Doctor of Public Health (DrPH) Student Information. updated 2014-07-24. https://www.hsph.harvard.edu/student-handbook/doctor-of-public-health-drph-student-information/ (2014). Accessed August 12 2019.

40. Harvard T.H. Chan School of Public Health: DrPH DELTA Doctoral Project Manual Class of 2019. https://cdn1.sph.harvard.edu/wp-content/uploads/sites/1496/1496/20/DrPH-Delta-Doctoral-Project-Manual-Cohort-3-1.pdf (2018). Accessed August 12 2019.
41. Harvard T.H. Chan School of Public Health: DrPH Program Student Manual - For students entering July 2019. https://cdn1.sph.harvard.edu/wp-content/uploads/sites/1496/2019/09/DrPH-Student-Manual-for-Class-of-2022.pdf (2018). Accessed August 12 2019.
42. Harvard T.H. Chan School of Public Health: The Harvard DrPH. https://www.hsph.harvard.edu/drph/ (2019). Accessed August 12 2019.
43. Johns Hopkins Bloomberg School of Public Health: Doctor of Public Health (DrPH). https://www.jhsph.edu/academics/degree-programs/doctoral-programs/doctor-of-public-health/index.html (2019). Accessed August 13 2019.
44. Johns Hopkins Bloomberg School of Public Health: Program Curriculum. https://www.jhsph.edu/academics/degree-programs/doctoral-programs/doctor-of-public-health/Curriculum.html (2019). Accessed August 13 2019.
45. Loma Linda University: Student Handbook 2019-2020. https://home.llu.edu/sites/home.llu.edu/files/docs/student-handbook.pdf (2019). Accessed August 13 2019.
46. Loma Linda University School of Public Health: Doctoral Programs. https://publichealth.llu.edu/academics/drph (2019). Accessed August 13 2019.
47. Loma Linda University School of Public Health: Doctoral Degrees. http://llucatalog.llu.edu/public-health/doctoral-degrees/doctoral-degrees.pdf (2019). Accessed August 13 2019.
48. Loma Linda University School of Public Health: Health Education — Dr.P.H. http://llucatalog.llu.edu/public-health/health-education-drph/#text (2019). Accessed August 13 2019.
49. Loma Linda University School of Public Health: Health Policy and Leadership — Dr.P.H. http://llucatalog.llu.edu/public-health/health-policy-leadership-drph/#text (2019). Accessed August 13 2019.
50. Loma Linda University School of Public Health: Preventive Care — Dr.P.H. (2019). Accessed August 13 2019.
51. New York Medical College School of Health Sciences and Practice & Institute of Public Health: Doctor of Public Health (Dr.P.H.). http://www.nymc.edu/school-of-health-sciences-and-practice-shsp/shsp-academics/degrees/doctor-of-public-health-drph/ (2019). Accessed August 13 2019.
52. Pennsylvania State University College of Medicine Public Health Program: DrPH Doctor of Public Health Program. https://med.psu.edu/drph (2019). Accessed August 13 2019.
53. Pennsylvania State University College of Medicine Public Health Program: 2019-20 Doctor of Public Health Handbook. https://students.med.psu.edu/doctor-of-public-health-drph/handbook/ (2019). Accessed August 13 2019.
54. SUNY Downstate Medical Center School of Public Health: Doctor of Public Health - Course Descriptions. https://www.downstate.edu/publichealth/programs/doctor-of-public-health.html (2019). Accessed August 13 2019.

55. SUNY Downstate Medical Center School of Public Health: 2019-2020 Student Handbook. https://sls.downstate.edu/student_affairs/_documents/student_handbooks/student_handbook_2019-20.pdf (2019). Accessed August 13 2019.
56. Texas A&M School of Public Health: Doctor of Public Health (DrPH). https://sph.tamhsc.edu/degrees/drph/index.html (2019). Accessed August 13 2019.
57. Texas A&M School of Public Health: Doctor of Public Health in Public Health Sciences. https://catalog.tamu.edu/graduate/colleges-schools-interdisciplinary/public-health/interdepartmental/public-health-sciences-drph/#text (2019). Accessed August 13 2019.
58. Tulane University School of Public Health and Tropical Medicine: DrPH in Global Community Health and Behavioral Sciences. https://sph.tulane.edu/gchb/drph (2019). Accessed August 13 2019.
59. University at Albany School of Public Health: Public Health Doctor of Public Health Degree Program (DrPH) - University at Albany-SUNY. https://www.albany.edu/graduatebulletin/public_health_drph_degree.htm (2019). Accessed August 13 2019.
60. University at Albany School of Public Health: Graduate Student Handbook 2017-2018. https://www.albany.edu/sph/assets/2017-2018_Graduate_Handbook_FINAL.pdf (2019). Accessed August 13 2019.
61. University of Alabama at Birmingham School of Public Health: Department of Biostatistics Graduate Student Handbook 2019-2020. https://www.soph.uab.edu/files/Student%20Handbooks/2019/BST_Graduate_Handbook_2019.pdf (2019). Accessed September 30 2019.
62. University of Alabama at Birmingham School of Public Health: Graduate Education. https://www.soph.uab.edu/graduate (2019). Accessed August 13 2019.
63. University of Arizona Mel and Enid Zuckerman College of Public Health: Doctor of Public Health (DrPH) in Maternal & Child Health | Mel and Enid Zuckerman College of Public Health. updated 2014-07-30T16:24-07:00. https://publichealth.arizona.edu/academics/doctoral-programs/drph-in-mch (2014). Accessed August 13 2019.
64. University of Arizona Mel and Enid Zuckerman College of Public Health: Doctor of Public Health (DrPH) in Public Health Policy and Management | Mel and Enid Zuckerman College of Public Health. updated 2014-07-30T16:10-07:00. https://publichealth.arizona.edu/academics/doctoral-programs/drph-in-phpm (2014). Accessed August 13 2019.
65. University of Arizona Mel and Enid Zuckerman College of Public Health: New Student Guide. https://publichealth.arizona.edu/sites/publichealth.arizona.edu/files/students/pdfs/New%20Student%20Guide%202018.pdf (2018). Accessed August 13 2019.
66. University of Arizona Mel and Enid Zuckerman College of Public Health: 2019-2020 DrPH-MCH Program Student Handbook. https://publichealth.arizona.edu/sites/publichealth.arizona.edu/files/MCH%202019-20%20Handbook.pdf (2019). Accessed September 30 2019.

67. University of Arkansas for Medical Sciences Fay W. Boozman College of Public Health: Doctor of Public Health in Public Health Leadership - Fay W. Boozman College of Public Health. https://publichealth.uams.edu/academics/doctoral/drph/ (2019). Accessed August 13 2019.
68. University of California Berkeley School of Public Health: Doctor of Public Health. updated 2013-05-24. https://sph.berkeley.edu/areas-study/doctor-public-health (2013). Accessed August 13 2019.
69. University of California Berkeley School of Public Health: 2019-20 DrPH Student Handbook. https://publichealth.berkeley.edu/wp-content/uploads/2019/08/2019-20_DrPH-Handbook.pdf (2019). Accessed September 30 2019.
70. University of Georgia College of Public Health: DrPH Residency Student Manual. publichealth.uga.edu/.../Doctor_of_Public_Health_Residency_Handbook_2019-2020 (2019). Accessed August 13 2019.
71. University of Georgia College of Public Health: Doctor of Public Health (DrPH) - College of Public Health UGA. https://publichealth.uga.edu/degree/doctor-of-public-health-drph/ (2019). Accessed August 13 2019.
72. University of Illinois at Chicago School of Public Health: Doctor of Public Health Leadership. https://publichealth.uic.edu/academics/public-health-degrees/drph/ (2019). Accessed December 30 2019.
73. University of Illinois at Chicago School of Public Health: FAQs. https://publichealth.uic.edu/academics/public-health-degrees/doctor-public-health-leadership/faqs/ (2019). Accessed August 13 2019.
74. University of Illinois at Chicago School of Public Health: 2019-20 The Doctor of Public Health Program Graduate Student Handbook. https://apps.sph.uic.edu/webdocs/pdf/shandbooks/DrPH_Student_Handbook_2019_2020_Final.pdf (2019). Accessed September 30 2019.
75. University of North Carolina Gillings School of Global Public Health: Doctoral Program in Health Leadership (DrPH) - Academic Policies, Guidelines, and Procedures. http://hpmadmittedstudents.web.unc.edu/files/2018/10/DrPH_Handbook_latest.pdf (2018). Accessed August 13 2019.
76. University of North Carolina Gillings School of Global Public Health: Programs Archive. https://sph.unc.edu/programs/ (2019). Accessed August 13 2019.
77. University of Puerto Rico Graduate School of Public Health: Doctorate in Public Health with Specialization in Health Systems Analysis and Management (DrPH HSAM). http://sp.rcm.upr.edu/asuntos-academicos/programas-academicos/doctorado-en-salud-publica-con-especialidad-en-analisis-de-sistemas-de-salud-y-gerencia-drph-hsam/ (2019). Accessed August 13 2019.
78. University of Puerto Rico Graduate School of Public Health: Doctorate in Public Health (DrPH) with Specialization in Environmental Health. http://sp.rcm.upr.edu/asuntos-academicos/programas-academicos/doctorado-en-salud-publica-drph-con-especialidad-en-salud-ambiental/ (2019). Accessed August 13 2019.
79. University of Puerto Rico Graduate School of Public Health: Doctorate in Public Health with Specialization in Social Determinants of Health. http://sp.rcm.upr.edu/asuntos-academicos/programas-academicos/doctorado-en-salud-publica-drph-con-especialidad-en-determinantes-sociales-de-la-salud/ (2019). Accessed August 13 2019.

80. University of Puerto Rico: Medical Sciences Campus Catalog 2017-2020. http://www.rcm.upr.edu/wp-content/uploads/sites/3/2019/01/UPR-MSC-CATALOG-2017-2020-REV-01092019.pdf (2017). Accessed August 13 2019.
81. University of South Florida College of Public Health: Doctor of Public Health Program (DrPH) Student Manual. https://health.usf.edu/-/media/Files/Public-Health/Global-Health/DrPHStudentManual11011.ashx (2011). Accessed August 13 2019.
82. University of South Florida College of Public Health: Doctor of Public Health (DrPH). https://health.usf.edu/publichealth/apply/graduate-admissions/drph (2019). Accessed August 13 2019.
83. University of Texas Health Science Center at Houston School of Public Health: General Information Catalog 2018–2020. https://www.uth.edu/academics/docs/school-catalogs/2018-2020-General-Information-FINAL.pdf (2018). Accessed August 13 2019.
84. University of Texas Health Science Center at Houston School of Public Health: Doctor of Public Health (DrPH). https://sph.uth.edu/academics/degree-programs/doctor-of-public-health-drph/ (2019). Accessed August 13 2019.
