# Supplementary material for: Analysis of CEPH-accredited DrPH programs in the United States: A mixed-methods study
Source: PLoS One. 2021 Feb 4;16(2):e0245892. doi: 10.1371/journal.pone.0245892 (PMC7861440; doi:10.1371/journal.pone.0245892)
Supplement: S3 Table — (PDF) [file pone.0245892.s003.pdf]

S3 Table. Structure of 28 CEPH-accredited DrPH schools and programs

School Name	Size of student group or cohort	Semester vs Quarter (Term)	Part-time vs Full-time Study	Coursework: On-site vs Online	Credits Needed for Graduation	Program Length
1. Boston University School of Public Health [1-3]	▪ 8 students per year<br>▪ Total: 45	Semester	Student's choice	On-site	48 credits	▪ Full time: maximum 5 years<br>▪ Part-time: maximum 7 years
2. Claremont Graduate University School of Community & Global Health [4-6]	13 new enrollments in 2018-2019 academic year	Semester	Student's choice	On-site	72 credits	5 years
3. University of Colorado School of Public Health [7-10]	▪ 3 new enrollments in 2018-2019 academic year<br>▪ Total program size in EOH: 8	Semester	Full-time at least first couple of years	On-site	55 credits	▪ 4 years at full-time effort<br>▪ Must complete within 7 years
4. Columbia University Mailman School of Public Health [11-16]	▪ DrPH in LGHHS: 4-5 per year (Total: 26)<br>▪ 8 new enrollments in 2018-	Semester	▪ Full-time is recommended during the first three semester<br>▪ After the second year, it	On-site	30–36 credits of coursework	▪ 4-5 years<br>▪ Time limit of 7 years

School Name	Size of student group or cohort	Semester vs Quarter (Term)	Part-time vs Full-time Study	Coursework: On-site vs Online	Credits Needed for Graduation	Program Length
	2019 academic year		is totally up to student's choice			
5. Drexel University Dornsife School of Public Health [17-19]	1 new enrollment in 2018-2019 academic year	Quarter	Student's choice	On-site	69 credits	▪ 4 years<br>▪ Maximum 7 years
6. East Tennessee State University College of Public Health [20-25]	6 new enrollments in 2018-2019 academic year	Semester	Student's choice	▪ Both.<br>▪ Domestic students can take online courses as many as they want.<br>▪ Internationa l students can take only one online course per semester.	58 credits	▪ Minimum 3 years to<br>▪ maximum 7 years.
7. George Washington University Milken Institute School of Public Health[29-33]<br><br>* School admits students every even year.	22 new enrollments in 2018-2019 academic year	Semester	Student's choice	On-site	48 credits	▪ Minimum: 3 years<br>▪ Maximum: 7 years
8. Georgia Southern University Jiann-Ping Hsu College of Public Health [34-38]	24 new enrollments in 2018-2019	Semester	Student's choice	▪ On-site<br>▪ Some courses are	60 credits	▪ Minimum 3 years

School Name	Size of student group or cohort	Semester vs Quarter (Term)	Part-time vs Full-time Study	Coursework: On-site vs Online	Credits Needed for Graduation	Program Length
	academic year			offered partially at a distance (face-to-face and online)		▪ Maximum 7 years
9. Georgia State University School of Public Health [26-28]	11 new enrollments in 2018-2019 academic year	Semester	Part-time	On-site	▪ For MPH graduate: 54 credits<br>▪ For non-MPH graduate: 54 credits + 9–19 credit of core public health prerequisites	3 years for students who work full-time, maximum of 7 years
10. Harvard T.H. Chan School of Public Health [39-42]	▪ 10 students per year<br>▪ Total program size: 30-35	Semester	Full-time	On-site	68.75 – 71.25 credits	▪ Three-year program.<br>▪ Having an option of being completed in four years.
11. Johns Hopkins Bloomberg School of Public Health [43, 44]	▪ 75-90 students will be recruited for 2020-2021 academic year<br>▪ Total program size: 234	Term	▪ Predominantly part-time program.<br>▪ In certain exceptional cases, full-time is allowed by contacting the	▪ Primarily online<br>▪ Required on-site course work in summer (for one weeks each June) and winter (for 5	▪ The number of credits varies depending on the applicant's prior master/s degrees.<br>▪ A minimum of 64 term credits must be taken (57 term credits	▪ Anticipating 4-6 years.<br>▪ Minimum 4 years to maximum 9 years

School Name	Size of student group or cohort	Semester vs Quarter (Term)	Part-time vs Full-time Study	Coursework: On-site vs Online	Credits Needed for Graduation	Program Length
			DrPH Program Office.	days in January) during the first and second year of the program.	of coursework + minimum of 7 term credits of proposal and dissertation work).	
12. Loma Linda University School of Public Health [45-50]	19 new enrollments in 2018-2019 academic year	Quarter unit	Student's choice	▪ On-site<br>▪ For Health Education: on-campus and as an on-line hybrid	62–65 units	▪ Minimum 3 years<br>▪ DrPH – Health Education: average 7 years<br>▪ DrPH – Health Policy and Leadership: average 3.5–5 years<br>▪ DrPH – Preventive Care: average 7 years
13. New York Medical College School of Health Sciences and Practice & Institute of Public Health [51]	▪ 7 students per year<br>▪ Total program size: 30	Semester	Student's choice	On-site	54 credits	▪ 3 years<br>▪ Typically, 4–5 years
14. Pennsylvania State University College of Medicine Public Health Program [52, 53]	▪ 7-10 per year<br>▪ Total program size: 35	Semester	Student's choice	On-site	60 credits	▪ Designed to be completed in 4 years<br>▪ 8-year limit

School Name	Size of student group or cohort	Semester vs Quarter (Term)	Part-time vs Full-time Study	Coursework: On-site vs Online	Credits Needed for Graduation	Program Length
15. SUNY Downstate Medical Center School of Public Health [54, 55]	10 new enrollments in 2018-2019 academic year	Semester	Student's choice	On-site	45 credits	Maximum: 8 years
16. Texas A&M School of Public Health [56, 57]	5–11 students per year	Semester	Student's choice	On-site	▪ Epidemiology and Environmental Health: 66 credits<br>▪ Health Promotion and Community Health Sciences: 54 credits	▪ Requires a minimum of 36 semester-credits of post-master's coursework or its equivalent (Full time: 9 credits in Fall/Spring; 6 credits in Summer)<br>▪ Maximum 10 years
17. Tulane University School of Public Health and Tropical Medicine [58]	3-4 per year	Semester	▪ Should be full-time for the first year.<br>▪ Student's choice from the second year.	On-site	▪ 72 credits<br>▪ Among 72 credits, 36 credits should be from doctoral study at Tulane	Must be completed within seven years
18. University at Albany School of Public Health [59, 60]	4 new enrollments in 2018-2019 academic year	Semester	▪ Student's choice<br>▪ Full-time study is encouraged.	On-site	▪ 54 credits<br>▪ Among 54 credits, at least 39 graduate credits should be taken at the	N/A

School Name	Size of student group or cohort	Semester vs Quarter (Term)	Part-time vs Full-time Study	Coursework: On-site vs Online	Credits Needed for Graduation	Program Length
					University at Albany	
19. University of Alabama at Birmingham School of Public Health [61, 62]	3 new enrollments in 2018-2019 academic year	Semester	Student's choice	On-site	42 credits	Allows maximum of 7 years
20. University of Arizona Mel and Enid Zuckerman College of Public Health [63-66]	▪ 2-3 students per year<br>▪ Total program size in MCH: 20<br>▪ Total in PHPM: less than 20	Semester	Student's choice	On-site	64 credits	▪ Average: 4.64 years; 5.08 years<br>▪ 3–5 years for full-time students (2-3 years for coursework, 1-2 years for dissertation work)<br>▪ 5–8 years for part-time students
21. University of Arkansas for Medical Sciences Fay W. Boozman College of Public Health [67]	2-3 students per year<br>Total program size: 12-15	Semester	Student's choice	On-site	61 credits	▪ Complete the qualifying exam within 5 years<br>▪ Maximum 10 years
22. University of California Berkeley School of Public Health [68, 69]	8-10 students per year<br>Total program size: 40-42	Semester	Full-time	On-site	60 credits	▪ 3 years<br>▪ 4th year is allowed as an exception.

School Name	Size of student group or cohort	Semester vs Quarter (Term)	Part-time vs Full-time Study	Coursework: On-site vs Online	Credits Needed for Graduation	Program Length
23. University of Georgia College of Public Health [70, 71]	15 students in 2017	Semester	Unknown – could be student's choice	On-site	57 credits	▪ 4 years<br>▪ Must be completed within 6 years
24. University of Illinois at Chicago School of Public Health [72-74]	16 new enrollments in 2018-2019 academic year	Semester	Part-time	Online	96 credits	Average 4.5 years
25. University of North Carolina Gillings School of Global Public Health [75, 76]	15 students per year	Semester	▪ Part-time<br>▪ All classes are provided via internet video one evening a week.	▪ Primarily online<br>▪ Required to visit campus in the fall, spring and summer (August, January, and May) during the two year of coursework (for 3-4 days each visit)<br>▪ On-campus visit can be replaced one time with a visit outside North	45-51 credits	Expecting 3 years

School Name	Size of student group or cohort	Semester vs Quarter (Term)	Part-time vs Full-time Study	Coursework: On-site vs Online	Credits Needed for Graduation	Program Length
				Carolina or overseas.		
26. University of Puerto Rico Graduate School of Public Health [77-80]	21 new enrollments in 2018-2019 academic year	Quarter	Unknown – could be student's choice	On-site	55 quarter credits	▪ Minimum 4 years<br>▪ Maximum 8 years
27. University of South Florida College of Public Health [81, 82]	19 new enrollments in 2018-2019 academic year	Semester	Part-time	▪ Primarily online<br>▪ Requires one-week campus sessions during each summer	43 credits	▪ Have 4 years to be admitted to doctoral candidacy<br>▪ Have another 4 years to complete degree requirements<br>▪ Maximum 8 years
28. University of Texas Health Science Center at Houston School of Public Health [83, 84]	6–11 students per year	Semester	Student's choice	On-site	48 credits	▪ 4 years<br>▪ Maximum 7 years

References

1. Boston University School of Public Health: Doctor of Public Health (DrPH). https://www.bu.edu/sph/education/degrees-and-programs/doctor-of-public-health-drph/ (2019). Accessed August 12 2019.
2. Boston University School of Public Health: DrPH Program Guidelines 2018-2019. https://www.bu.edu/sph/files/2018/10/DrPH-Handbook-2018.pdf (2018). Accessed August 12 2019.
3. Boston University School of Public Health: Transfer credit or waiver policies. https://www.bu.edu/sph/students/advising-and-registration/policies-and-procedures/policies/transfer-credit-or-waiver-policies/ (2019). Accessed August 12 2019.
4. Claremont Graduate University School of Community & Global Health: Doctor of Public Health. https://www.cgu.edu/academics/program/doctor-public-health/ (2019). Accessed August 12 2019.
5. Claremont Graduate University School of Community & Global Health: Archived Bulletin - Public Health, DrPH. http://bulletin.cgu.edu/preview_program.php?catoid=13&poid=1646&returnto=1595 (2019). Accessed August 12 2019.
6. Claremont Graduate University School of Community & Global Health: Archived Bulletin - Registration & Enrollment. http://bulletin.cgu.edu/content.php?catoid=13&navoid=1511#Transfer (2019). Accessed August 12 2019.
7. Colorado School of Public Health: Doctor of Public Health - DrPH Programs at the Colorado School of Public Health. http://www.ucdenver.edu/academics/colleges/PublicHealth/Academics/degreesandprograms/Pages/DrPH.aspx (2019). Accessed August 12 2019.
8. Colorado School of Public Health: Doctor of Public Health 2019-2020 Student Handbook. http://www.ucdenver.edu/academics/colleges/PublicHealth/resourcesfor/currentstudents/academics/Documents/19_20_Handbooks/DRPH_Handbook_19_20.pdf (2019). Accessed September 30 2019.
9. Colorado School of Public Health: Practicum & Capstone. http://www.ucdenver.edu/academics/colleges/PublicHealth/resourcesfor/currentstudents/academics/Pages/PracticeBasedLearning.aspx (2019). Accessed August 12 2019.
10. Colorado School of Public Health: DrPH Requirements. http://www.ucdenver.edu/academics/colleges/PublicHealth/admissionsandaid/howtoapply/Pages/DrPHReqs.aspx (2019). Accessed August 12 2019.
11. Columbia University Mailman School of Public Health: Doctoral Guidelines - PhD and DrPH Programs in Epidemiology. https://www.mailman.columbia.edu/sites/default/files/pdf/doctoral_guidelines.pdf (2017). Accessed August 12 2019.
12. Columbia University Mailman School of Public Health: The Department of Biostatistics Student Handbook 2019-2020. https://www.mailman.columbia.edu/sites/default/files/biostats_student_handbook_2019-2020.pdf (2018). Accessed September 30 2019.

13. Columbia University Mailman School of Public Health: DrPH. https://www.mailman.columbia.edu/academics/degrees/degree-requirements/drph (2019). Accessed August 12 2019.
14. Columbia University Mailman School of Public Health: Department of Sociomedical Sciences Doctoral Student Handbook 2019-2020. https://www.mailman.columbia.edu/sites/default/files/pdf/sms-doctoral-handbook-2019-20.pdf (2019). Accessed September 30 2019.
15. Columbia University Mailman School of Public Health: Doctoral Student Handbook. https://www.mailman.columbia.edu/sites/default/files/pdf/doctoral-handbook_2.pdf (2018). Accessed August 12 2019.
16. Columbia University Mailman School of Public Health: Heilbrunn Department of Population & Family Health Doctoral Program Handbook Version 4.1 (March 2018). https://www.mailman.columbia.edu/sites/default/files/pdf/pfh-drph-handbook-march-2018.pdf (2019). Accessed January 8 2020.
17. Drexel University Dornsife School of Public Health. DSPH Student Handbook AY 2018 to 2019. 2018.
18. Drexel University Dornsife School of Public Health: DrPH in Health Management & Policy. https://drexel.edu/dornsife/academics/degrees/drph-in-health-management-and-policy/ (2019). Accessed August 12 2019.
19. Drexel University Dornsife School of Public Health: Health Management and Policy DrPH - About the Program. http://catalog.drexel.edu/graduate/schoolofpublichealth/healthmanagementandpolicydrph/#text (2019). Accessed August 12 2019.
20. East Tennessee State University College of Public Health: DrPH Field Experience Guidelines 2017-18. https://www.etsu.edu/cph/documents/drphfieldexperienceguidelines.pdf (2017). Accessed August 12 2019.
21. East Tennessee State University College of Public Health: Graduate Health Professions Education Doctor of Public Health 2019-2020 Student Handbook. https://www.etsu.edu/cph/documents/drphhandbook.pdf (2018). Accessed January 9 2020.
22. East Tennessee State University College of Public Health: Doctoral Programs. https://www.etsu.edu/cph/academics/doctoral.php#tab-7-1 (2019). Accessed August 12 2019.
23. East Tennessee State University College of Public Health: Archived Catalog - Public Health, Dr.P.H. (Community Health Concentration). https://catalog.etsu.edu/preview_program.php?catoid=27&poid=10427&returnto=1347 (2019). Accessed August 12 2019.
24. East Tennessee State University College of Public Health: Archived Catalog - Public Health, Dr.P.H. (Epidemiology Concentration). https://catalog.etsu.edu/preview_program.php?catoid=27&poid=10428&returnto=1347 (2019). Accessed August 12 2019.
25. East Tennessee State University College of Public Health: Archived Catalog - Public Health, Dr.P.H. (Health Management and Policy Concentration). https://catalog.etsu.edu/preview_program.php?catoid=27&poid=10502&returnto=1347 (2019). Accessed August 12 2019.
26. Georgia State University School of Public Health: Doctor of Public Health - School of Public Health. https://publichealth.gsu.edu/academics-student-life/degrees-programs/drph/ (2019). Accessed August 12 2019.

27. Georgia State University School of Public Health: Doctor of Public Health - Frequently Asked Questions. https://publichealth.gsu.edu/academics-student-life/degrees-programs/drph-faq/ (2019). Accessed August 12 2019.
28. Georgia State University School of Public Health: Doctor of Public Health - How to Apply. https://publichealth.gsu.edu/academics-student-life/degrees-programs/drph-apply/ (2019). Accessed August 12 2019.
29. George Washington University Milken Institute School of Public Health: Program Guide - Doctor of Public Health, Environmental and Occupational Health. https://publichealth.gwu.edu/sites/default/files/DrPH%20EOH%202018%20%28May%202018%29.pdf (2018). Accessed August 12 2019.
30. George Washington University Milken Institute School of Public Health: Program Guide - Doctor of Public Health, Global Health. https://publichealth.gwu.edu/sites/default/files/DrPH%20Global%20Health%202018_0.pdf (2018). Accessed August 12 2019.
31. George Washington University Milken Institute School of Public Health: Program Guide - Doctor of Public Health, Health Behavior. https://publichealth.gwu.edu/sites/default/files/DrPH%20HB%202018_0.pdf (2018). Accessed August 12 2019.
32. George Washington University Milken Institute School of Public Health: Program Guide - Doctor of Public Health, Health Policy. https://publichealth.gwu.edu/sites/default/files/DrPH%20Health%20Policy%202018_0.pdf (2018). Accessed August 12 2019.
33. George Washington University Milken Institute School of Public Health: DrPH Programs. https://publichealth.gwu.edu/academics/graduate/drph-programs (2019). Accessed August 12 2019.
34. Georgia Southern University Jiann-Ping Hsu College of Public Health: Graduate Assistantship (GA) Handbook 2017-2018. https://docs.google.com/file/d/0B2ms15eoGveqMkJKS2J6U3pKSm8/edit?usp=embed_facebook (2017). Accessed August 12 2019.
35. Georgia Southern University Jiann-Ping Hsu College of Public Health: Student Handbook 2017-2018. https://drive.google.com/file/d/0B2ms15eoGveqOHlEUW53X0ZSWVE/view?usp=embed_facebook (2017). Accessed August 12 2019.
36. Georgia Southern University Jiann-Ping Hsu College of Public Health: Site Supervisor Handbook 2017-2018. https://drive.google.com/file/d/0B2ms15eoGveqdFNnTldkUlcyMVE/view?usp=embed_facebook (2017). Accessed August 12 2019.
37. Georgia Southern University Jiann-Ping Hsu College of Public Health: Degrees & Programs. https://jphcoph.georgiasouthern.edu/degrees/#DrPHDegree (2019). Accessed August 12 2019.
38. Georgia Southern University Jiann-Ping Hsu College of Public Health: Catalog 2019-2020 - Doctor of Public Health. https://catalog.georgiasouthern.edu/graduate/jiann-ping-hsu-public-health/doctor-public-health/ (2019). Accessed August 12 2019.

39. Harvard T.H. Chan School of Public Health: Doctor of Public Health (DrPH) Student Information. updated 2014-07-24. https://www.hsph.harvard.edu/student-handbook/doctor-of-public-health-drph-student-information/ (2014). Accessed August 12 2019.
40. Harvard T.H. Chan School of Public Health: DrPH DELTA Doctoral Project Manual Class of 2019. https://cdn1.sph.harvard.edu/wp-content/uploads/sites/1496/1496/20/DrPH-Delta-Doctoral-Project-Manual-Cohort-3-1.pdf (2018). Accessed August 12 2019.
41. Harvard T.H. Chan School of Public Health: DrPH Program Student Manual - For students entering July 2019. https://cdn1.sph.harvard.edu/wp-content/uploads/sites/1496/2019/09/DrPH-Student-Manual-for-Class-of-2022.pdf (2018). Accessed August 12 2019.
42. Harvard T.H. Chan School of Public Health: The Harvard DrPH. https://www.hsph.harvard.edu/drph/ (2019). Accessed August 12 2019.
43. Jackson State University School of Public Health: Doctor of Public Health Degree Admission Requirements. http://www.jsums.edu/health/files/2018/05/DrPH-Admission-Requirements-1.pdf (2019). Accessed August 13 2019.
44. Jackson State University School of Public Health: School of Public Health Doctor of Public Health Degree Student Handbook,. http://www.jsums.edu/health/files/2017/12/DrPHStudentHandbook-2017-2019.pdf?x17560 (2017). Accessed August 13 2019.
45. Loma Linda University: Student Handbook 2019-2020. https://home.llu.edu/sites/home.llu.edu/files/docs/student-handbook.pdf (2019). Accessed August 13 2019.
46. Loma Linda University School of Public Health: Doctoral Programs. https://publichealth.llu.edu/academics/drph (2019). Accessed August 13 2019.
47. Loma Linda University School of Public Health: Doctoral Degrees. http://llucatalog.llu.edu/public-health/doctoral-degrees/doctoral-degrees.pdf (2019). Accessed August 13 2019.
48. Loma Linda University School of Public Health: Health Education — Dr.P.H. http://llucatalog.llu.edu/public-health/health-education-drph/#text (2019). Accessed August 13 2019.
49. Loma Linda University School of Public Health: Health Policy and Leadership — Dr.P.H. http://llucatalog.llu.edu/public-health/health-policy-leadership-drph/#text (2019). Accessed August 13 2019.
50. Loma Linda University School of Public Health: Preventive Care — Dr.P.H. (2019). Accessed August 13 2019.
51. New York Medical College School of Health Sciences and Practice & Institute of Public Health: Doctor of Public Health (Dr.P.H.). http://www.nymc.edu/school-of-health-sciences-and-practice-shsp/shsp-academics/degrees/doctor-of-public-health-drph/ (2019). Accessed August 13 2019.
52. Pennsylvania State University College of Medicine Public Health Program: DrPH Doctor of Public Health Program. https://med.psu.edu/drph (2019). Accessed August 13 2019.
53. Pennsylvania State University College of Medicine Public Health Program: 2019-20 Doctor of Public Health Handbook. https://students.med.psu.edu/doctor-of-public-health-drph/handbook/ (2019). Accessed August 13 2019.

54. SUNY Downstate Medical Center School of Public Health: Doctor of Public Health - Course Descriptions. https://www.downstate.edu/publichealth/programs/doctor-of-public-health.html (2019). Accessed August 13 2019.
55. SUNY Downstate Medical Center School of Public Health: 2019-2020 Student Handbook. https://sls.downstate.edu/student_affairs/_documents/student_handbooks/student_handbook_2019-20.pdf (2019). Accessed August 13 2019.
56. Texas A&M School of Public Health: Doctor of Public Health (DrPH). https://sph.tamhsc.edu/degrees/drph/index.html (2019). Accessed August 13 2019.
57. Texas A&M School of Public Health: Doctor of Public Health in Public Health Sciences. https://catalog.tamu.edu/graduate/colleges-schools-interdisciplinary/public-health/interdepartmental/public-health-sciences-drph/#text (2019). Accessed August 13 2019.
58. Tulane University School of Public Health and Tropical Medicine: DrPH in Global Community Health and Behavioral Sciences. https://sph.tulane.edu/gchb/drph (2019). Accessed August 13 2019.
59. University at Albany School of Public Health: Public Health Doctor of Public Health Degree Program (DrPH) - University at Albany-SUNY. https://www.albany.edu/graduatebulletin/public_health_drph_degree.htm (2019). Accessed August 13 2019.
60. University at Albany School of Public Health: Graduate Student Handbook 2017-2018. https://www.albany.edu/sph/assets/2017-2018_Graduate_Handbook_FINAL.pdf (2019). Accessed August 13 2019.
61. University of Alabama at Birmingham School of Public Health: Department of Biostatistics Graduate Student Handbook 2019-2020. https://www.soph.uab.edu/files/Student%20Handbooks/2019/BST_Graduate_Handbook_2019.pdf (2019). Accessed September 30 2019.
62. University of Alabama at Birmingham School of Public Health: Graduate Education. https://www.soph.uab.edu/graduate (2019). Accessed August 13 2019.
63. University of Arizona Mel and Enid Zuckerman College of Public Health: Doctor of Public Health (DrPH) in Maternal & Child Health | Mel and Enid Zuckerman College of Public Health. updated 2014-07-30T16:24-07:00. https://publichealth.arizona.edu/academics/doctoral-programs/drph-in-mch (2014). Accessed August 13 2019.
64. University of Arizona Mel and Enid Zuckerman College of Public Health: Doctor of Public Health (DrPH) in Public Health Policy and Management | Mel and Enid Zuckerman College of Public Health. updated 2014-07-30T16:10-07:00. https://publichealth.arizona.edu/academics/doctoral-programs/drph-in-phpm (2014). Accessed August 13 2019.
65. University of Arizona Mel and Enid Zuckerman College of Public Health: New Student Guide. https://publichealth.arizona.edu/sites/publichealth.arizona.edu/files/students/pdfs/New%20Student%20Guide%202018.pdf (2018). Accessed August 13 2019.
66. University of Arizona Mel and Enid Zuckerman College of Public Health: 2019-2020 DrPH-MCH Program Student Handbook. https://publichealth.arizona.edu/sites/publichealth.arizona.edu/files/MCH%202019-20%20Handbook.pdf (2019). Accessed September 30 2019.

67. University of Arkansas for Medical Sciences Fay W. Boozman College of Public Health: Doctor of Public Health in Public Health Leadership - Fay W. Boozman College of Public Health. https://publichealth.uams.edu/academics/doctoral/drph/ (2019). Accessed August 13 2019.
68. University of California Berkeley School of Public Health: Doctor of Public Health. updated 2013-05-24. https://sph.berkeley.edu/areas-study/doctor-public-health (2013). Accessed August 13 2019.
69. University of California Berkeley School of Public Health: 2019-20 DrPH Student Handbook. https://publichealth.berkeley.edu/wp-content/uploads/2019/08/2019-20_DrPH-Handbook.pdf (2019). Accessed September 30 2019.
70. University of Georgia College of Public Health: DrPH Residency Student Manual. publichealth.uga.edu/.../Doctor_of_Public_Health_Residency_Handbook_2019-2020 (2019). Accessed August 13 2019.
71. University of Georgia College of Public Health: Doctor of Public Health (DrPH) - College of Public Health UGA. https://publichealth.uga.edu/degree/doctor-of-public-health-drph/ (2019). Accessed August 13 2019.
72. University of Illinois at Chicago School of Public Health: Doctor of Public Health Leadership. https://publichealth.uic.edu/academics/public-health-degrees/drph/ (2019). Accessed December 30 2019.
73. University of Illinois at Chicago School of Public Health: FAQs. https://publichealth.uic.edu/academics/public-health-degrees/doctor-public-health-leadership/faqs/ (2019). Accessed August 13 2019.
74. University of Illinois at Chicago School of Public Health: 2019-20 The Doctor of Public Health Program Graduate Student Handbook. https://apps.sph.uic.edu/webdocs/pdf/shandbooks/DrPH_Student_Handbook_2019_2020_Final.pdf (2019). Accessed September 30 2019.
75. University of North Carolina Gillings School of Global Public Health: Doctoral Program in Health Leadership (DrPH) - Academic Policies, Guidelines, and Procedures. http://hpmadmittedstudents.web.unc.edu/files/2018/10/DrPH_Handbook_latest.pdf (2018). Accessed August 13 2019.
76. University of North Carolina Gillings School of Global Public Health: Programs Archive. https://sph.unc.edu/programs/ (2019). Accessed August 13 2019.
77. University of Puerto Rico Graduate School of Public Health: Doctorate in Public Health with Specialization in Health Systems Analysis and Management (DrPH HSAM). http://sp.rcm.upr.edu/asuntos-academicos/programas-academicos/doctorado-en-salud-publica-con-especialidad-en-analisis-de-sistemas-de-salud-y-gerencia-drph-hsam/ (2019). Accessed August 13 2019.
78. University of Puerto Rico Graduate School of Public Health: Doctorate in Public Health (DrPH) with Specialization in Environmental Health. http://sp.rcm.upr.edu/asuntos-academicos/programas-academicos/doctorado-en-salud-publica-drph-con-especialidad-en-salud-ambiental/ (2019). Accessed August 13 2019.
79. University of Puerto Rico Graduate School of Public Health: Doctorate in Public Health with Specialization in Social Determinants of Health. http://sp.rcm.upr.edu/asuntos-academicos/programas-academicos/doctorado-en-salud-publica-drph-con-especialidad-en-determinantes-sociales-de-la-salud/ (2019). Accessed August 13 2019.

80. University of Puerto Rico: Medical Sciences Campus Catalog 2017-2020. http://www.rcm.upr.edu/wp-content/uploads/sites/3/2019/01/UPR-MSC-CATALOG-2017-2020-REV-01092019.pdf (2017). Accessed August 13 2019.
81. University of South Florida College of Public Health: Doctor of Public Health Program (DrPH) Student Manual. https://health.usf.edu/-/media/Files/Public-Health/Global-Health/DrPHStudentManual11011.ashx (2011). Accessed August 13 2019.
82. University of South Florida College of Public Health: Doctor of Public Health (DrPH). https://health.usf.edu/publichealth/apply/graduate-admissions/drph (2019). Accessed August 13 2019.
83. University of Texas Health Science Center at Houston School of Public Health: General Information Catalog 2018–2020. https://www.uth.edu/academics/docs/school-catalogs/2018-2020-General-Information-FINAL.pdf (2018). Accessed August 13 2019.
84. University of Texas Health Science Center at Houston School of Public Health: Doctor of Public Health (DrPH). https://sph.uth.edu/academics/degree-programs/doctor-of-public-health-drph/ (2019). Accessed August 13 2019.
