# Supplementary material for: Analysis of CEPH-accredited DrPH programs in the United States: A mixed-methods study
Source: PLoS One. 2021 Feb 4;16(2):e0245892. doi: 10.1371/journal.pone.0245892 (PMC7861440; doi:10.1371/journal.pone.0245892)
Supplement: S5 Table — (PDF) [file pone.0245892.s005.pdf]

S5 Table. Comprehensive/qualifying exam

School Name	Type of DrPH Comprehensive/Qualifying Exam<br>(First bullet: type of comprehensive exam, Second bullet: format of comprehensive exam)
1. Boston University School of Public Health [1-3]	▪ Schoolwide<br>▪ Written + Oral:<br>1) Three formats:<br>i. a 25-page written report<br>ii. a 2-page memo to a policymaker: *A) presents the student's advocacy for the plan in a political context and B) proves persuasive to current political leaders on their terms*<br>iii. orally: a brief (15 minutes maximum) presentation followed by a question and answer period that may cover all three products<br>2) Each student will be required to select and respond to one of the two real-life public health situations, one U.S.-based and one international, presented by the examiners.
2. Claremont Graduate University School of Community & Global Health [4-6]	▪ Schoolwide<br>▪ Written + Oral
3. Colorado School of Public Health [7-10]	▪ Departmental<br>▪ Directed Reading course + Written + Oral:<br>1) Directed Reading course: Students should take 2-credit Directed Reading course before their comprehensive exams and dissertation research.<br>2) Written comprehensive exam: Upon completion of the Directed Reading course, students take 2-week written comprehensive exam including 3-5 essay questions.<br>3) Oral comprehensive exam: 30-40 minute presentation + up to 2 hours for committee feedback and questions
4. Columbia University Mailman School of Public Health [11-16]	▪ Departmental<br>1) DrPH – Biostatistics: Three part—Basic inference, Statistical Applications Exam, Oral comprehensive examination<br>2) DrPH – Environmental Health Sciences: N/A

School Name	Type of DrPH Comprehensive/Qualifying Exam<br>(First bullet: type of comprehensive exam, Second bullet: format of comprehensive exam)
	3) DrPH – Epidemiology: Two qualifying examinations (the Methods Examination and the Foundation Essay) + two oral dissertation proposal defenses (internal defense + public defense)<br>4) DrPH – Leadership in Global Health and Humanitarian Systems: Two qualifying examinations—the Methods Examination (from required methods coursework) and the Substantive Examination (two components: a comprehensive reading list and an oral exam)<br>5) DrPH – Sociomedical Sciences: Methods and Theme Essays
5. Drexel University Dornsife School of Public Health [17-19]	▪ Departmental<br>▪ Written + Oral
6. East Tennessee State University College of Public Health [20-25]	▪ Departmental<br>▪ Written + Oral
7. George Washington University Milken Institute School of Public Health [26-30]	▪ Departmental<br>▪ Written + Dissertation Course + Oral (Written comprehensive examination + Dissertation course for the preparation of proposal + The dissertation proposal defense)
8. Georgia Southern University Jiann-Ping Hsu College of Public Health [31-35]	▪ Departmental (Concentration-specific candidacy examination).<br>▪ Format is unknown.
9. Georgia State University School of Public Health [36-38]	▪ Schoolwide<br>▪ Format is unknown.
10. Harvard T.H. Chan School of Public Health [39-42]	▪ Schoolwide<br>▪ Written + Closed book in class exam + Oral:<br>1) Three deliverables for written qualifying examination:<br>i. Deliverable #1: Analysis and interpretation of a public health related dataset in the format of a 3 to 5-page paper.<br>ii. Deliverable #2: Development of a PowerPoint presentation for senior leadership regarding organizational, financial and operational impact of proposed major change (maximum of 15 slides).<br>iii. Deliverable #3: 3-hour closed book in class exam. Writing of policy memo on a public health related issue with analysis and recommendations. Student may choose a domestic policy or international policy question.

School Name	Type of DrPH Comprehensive/Qualifying Exam<br>(First bullet: type of comprehensive exam, Second bullet: format of comprehensive exam)
	2) For oral qualifying examination: 90-minute examination with the Doctoral Project Committee (First 30-minute: proposal presentation + remaining minutes: Q&A and advice/suggestions from the Doctoral Project Committee)
11. Johns Hopkins Bloomberg School of Public Health [43, 44]	▪ Schoolwide + departmental<br>▪ Written + Oral:<br>1) Comprehensive exam: departmental<br>2) Preliminary oral exam: school-wide
12. Loma Linda University School of Public Health [45-50]	▪ Schoolwide<br>▪ Written + Oral:<br>1) Written comprehensive exam<br>2) Oral qualifying exam: dissertation proposal defense
13. New York Medical College School of Health Sciences [51]	▪ Program/concentration<br>▪ Format is unknown.
14. Pennsylvania State University College of Medicine Public Health Program [52, 53]	▪ Program/concentration<br>▪ Qualifying exam + Pre-comps meeting + Written comprehensive exam + Oral:<br>1) Qualifying exam: format is unknown.<br>2) Pre-comps meeting: discuss the student's specific aims and the academic domains which the student's exam will cover.<br>3) Written comprehensive exam: take-home exam<br>4) Oral examination
15. SUNY Downstate Medical Center School of Public Health [54, 55]	▪ Schoolwide + departmental<br>▪ Closed book written exam:<br>1) Day 1: 6-hour exam within the department<br>2) Day 2: 6-hour exam from Doctor of Public Health core courses
16. Texas A&M School of Public Health [56, 57]	▪ Unknown<br>▪ Written, Oral, or Combination of written and oral
17. Tulane University School of Public Health and Tropical Medicine [58]	▪ Departmental<br>▪ Written
18. University at Albany School of Public Health [59, 60]	▪ Might be schoolwide.<br>▪ Format is unknown.

School Name	Type of DrPH Comprehensive/Qualifying Exam<br>(First bullet: type of comprehensive exam, Second bullet: format of comprehensive exam)
19. University of Alabama at Birmingham School of Public Health [61, 62]	▪ Departmental<br>▪ Written + readings courses + Oral<br>1) DrPH in Health Care Organization and Policy: should take 3-credit Directed Readings for DrPH Comprehensive Exam, 2-day, 9am-5pm exam—Day 1 (Part I): General Public Health, Day 2 (Part II): Research Methodology, Part III: Oral examination<br>2) DrPH in biostatistics: 2-week written comprehensive exam, register for readings courses + oral examination (proposal defense)
20. University of Arizona Mel and Enid Zuckerman College of Public Health [63-66]	▪ Departmental<br>▪ Closed book exam + Written + Oral<br>1) Written exam Day 1: 8-hour proctored exam<br>2) Written exam Day 2: 8-hour take home exam<br>3) Oral exam: 20-minute presentation for policy position from Day 2 exam, 30-minute Q&A, 1 hour general questions
21. University of Arkansas Fay W. Boozman College of Public Health [67]	▪ Collegewide<br>▪ Closed book exam + Oral
22. University of California Berkeley School of Public Health [68, 69]	▪ Schoolwide<br>▪ Written + Oral
23. University of Georgia College of Public Health [70, 71]	▪ Departmental<br>▪ Written + Oral
24. University of Illinois at Chicago School of Public Health [72-74]	▪ The portfolio—integrative document of the student's professional and academic experiences—replaces the usual doctoral preliminary and qualifying exams.
25. University of North Carolina Gillings School of Global Public Health [75, 76]	▪ Departmental<br>▪ Written
26. University of Puerto Rico Graduate School of Public Health [77-80]	▪ Unknown<br>▪ Written
27. University of South Florida College of Public Health [81, 82]	▪ Schoolwide<br>▪ Written + Optional Oral
28. University of Texas Health Science Center at Houston School of Public Health [83, 84]	▪ Unknown<br>▪ Format is unknown.

References

1. Boston University School of Public Health: Doctor of Public Health (DrPH). https://www.bu.edu/sph/education/degrees-and-programs/doctor-of-public-health-drph/ (2019). Accessed August 12 2019.
2. Boston University School of Public Health: DrPH Program Guidelines 2018-2019. https://www.bu.edu/sph/files/2018/10/DrPH-Handbook-2018.pdf (2018). Accessed August 12 2019.
3. Boston University School of Public Health: Transfer credit or waiver policies. https://www.bu.edu/sph/students/advising-and-registration/policies-and-procedures/policies/transfer-credit-or-waiver-policies/ (2019). Accessed August 12 2019.
4. Claremont Graduate University School of Community & Global Health: Doctor of Public Health. https://www.cgu.edu/academics/program/doctor-public-health/ (2019). Accessed August 12 2019.
5. Claremont Graduate University School of Community & Global Health: Archived Bulletin - Public Health, DrPH. http://bulletin.cgu.edu/preview_program.php?catoid=13&poid=1646&returnto=1595 (2019). Accessed August 12 2019.
6. Claremont Graduate University School of Community & Global Health: Archived Bulletin - Registration & Enrollment. http://bulletin.cgu.edu/content.php?catoid=13&navoid=1511#Transfer (2019). Accessed August 12 2019.
7. Colorado School of Public Health: Doctor of Public Health - DrPH Programs at the Colorado School of Public Health. http://www.ucdenver.edu/academics/colleges/PublicHealth/Academics/degreesandprograms/Pages/DrPH.aspx (2019). Accessed August 12 2019.
8. Colorado School of Public Health: Doctor of Public Health 2019-2020 Student Handbook. http://www.ucdenver.edu/academics/colleges/PublicHealth/resourcesfor/currentstudents/academics/Documents/19_20_Handbooks/DRPH_Handbook_19_20.pdf (2019). Accessed September 30 2019.
9. Colorado School of Public Health: Practicum & Capstone. http://www.ucdenver.edu/academics/colleges/PublicHealth/resourcesfor/currentstudents/academics/Pages/PracticeBasedLearning.aspx (2019). Accessed August 12 2019.
10. Colorado School of Public Health: DrPH Requirements. http://www.ucdenver.edu/academics/colleges/PublicHealth/admissionsandaid/howtoapply/Pages/DrPHReqs.aspx (2019). Accessed August 12 2019.
11. Columbia University Mailman School of Public Health: Doctoral Guidelines - PhD and DrPH Programs in Epidemiology. https://www.mailman.columbia.edu/sites/default/files/pdf/doctoral_guidelines.pdf (2017). Accessed August 12 2019.
12. Columbia University Mailman School of Public Health: The Department of Biostatistics Student Handbook 2019-2020. https://www.mailman.columbia.edu/sites/default/files/biostats_student_handbook_2019-2020.pdf (2018). Accessed September 30 2019.
13. Columbia University Mailman School of Public Health: DrPH. https://www.mailman.columbia.edu/academics/degrees/degree-requirements/drph (2019). Accessed August 12 2019.

14. Columbia University Mailman School of Public Health: Department of Sociomedical Sciences Doctoral Student Handbook 2019-2020. https://www.mailman.columbia.edu/sites/default/files/pdf/sms-doctoral-handbook-2019-20.pdf (2019). Accessed September 30 2019.
15. Columbia University Mailman School of Public Health: Doctoral Student Handbook. https://www.mailman.columbia.edu/sites/default/files/pdf/doctoral-handbook_2.pdf (2018). Accessed August 12 2019.
16. Columbia University Mailman School of Public Health: Heilbrunn Department of Population & Family Health Doctoral Program Handbook Version 4.1 (March 2018). https://www.mailman.columbia.edu/sites/default/files/pdf/pfh-drph-handbook-march-2018.pdf (2019). Accessed January 8 2020.
17. Drexel University Dornsife School of Public Health. DSPH Student Handbook AY 2018 to 2019. 2018.
18. Drexel University Dornsife School of Public Health: DrPH in Health Management & Policy. https://drexel.edu/dornsife/academics/degrees/drph-in-health-management-and-policy/ (2019). Accessed August 12 2019.
19. Drexel University Dornsife School of Public Health: Health Management and Policy DrPH - About the Program. http://catalog.drexel.edu/graduate/schoolofpublichealth/healthmanagementandpolicydrph/#text (2019). Accessed August 12 2019.
20. East Tennessee State University College of Public Health: DrPH Field Experience Guidelines 2017-18. https://www.etsu.edu/cph/documents/drphfieldexperienceguidelines.pdf (2017). Accessed August 12 2019.
21. East Tennessee State University College of Public Health: Graduate Health Professions Education Doctor of Public Health 2019-2020 Student Handbook. https://www.etsu.edu/cph/documents/drphhandbook.pdf (2018). Accessed January 9 2020.
22. East Tennessee State University College of Public Health: Doctoral Programs. https://www.etsu.edu/cph/academics/doctoral.php#tab-7-1 (2019). Accessed August 12 2019.
23. East Tennessee State University College of Public Health: Archived Catalog - Public Health, Dr.P.H. (Community Health Concentration). https://catalog.etsu.edu/preview_program.php?catoid=27&poid=10427&returnto=1347 (2019). Accessed August 12 2019.
24. East Tennessee State University College of Public Health: Archived Catalog - Public Health, Dr.P.H. (Epidemiology Concentration). https://catalog.etsu.edu/preview_program.php?catoid=27&poid=10428&returnto=1347 (2019). Accessed August 12 2019.
25. East Tennessee State University College of Public Health: Archived Catalog - Public Health, Dr.P.H. (Health Management and Policy Concentration). https://catalog.etsu.edu/preview_program.php?catoid=27&poid=10502&returnto=1347 (2019). Accessed August 12 2019.
26. George Washington University Milken Institute School of Public Health: Program Guide - Doctor of Public Health, Environmental and Occupational Health. https://publichealth.gwu.edu/sites/default/files/DrPH%20EOH%202018%20%28May%202018%29.pdf (2018). Accessed August 12 2019.

27. George Washington University Milken Institute School of Public Health: Program Guide - Doctor of Public Health, Global Health. https://publichealth.gwu.edu/sites/default/files/DrPH%20Global%20Health%202018_0.pdf (2018). Accessed August 12 2019.
28. George Washington University Milken Institute School of Public Health: Program Guide - Doctor of Public Health, Health Behavior. https://publichealth.gwu.edu/sites/default/files/DrPH%20HB%202018_0.pdf (2018). Accessed August 12 2019.
29. George Washington University Milken Institute School of Public Health: Program Guide - Doctor of Public Health, Health Policy. https://publichealth.gwu.edu/sites/default/files/DrPH%20Health%20Policy%202018_0.pdf (2018). Accessed August 12 2019.
30. George Washington University Milken Institute School of Public Health: DrPH Programs. https://publichealth.gwu.edu/academics/graduate/drph-programs (2019). Accessed August 12 2019.
31. Georgia Southern University Jiann-Ping Hsu College of Public Health: Graduate Assistantship (GA) Handbook 2017-2018. https://docs.google.com/file/d/0B2ms15eoGveqMkJKS2J6U3pKSm8/edit?usp=embed_facebook (2017). Accessed August 12 2019.
32. Georgia Southern University Jiann-Ping Hsu College of Public Health: Student Handbook 2017-2018. https://drive.google.com/file/d/0B2ms15eoGveqOHlEUW53X0ZSWVE/view?usp=embed_facebook (2017). Accessed August 12 2019.
33. Georgia Southern University Jiann-Ping Hsu College of Public Health: Site Supervisor Handbook 2017-2018. https://drive.google.com/file/d/0B2ms15eoGveqdFNnTldkUlcyMVE/view?usp=embed_facebook (2017). Accessed August 12 2019.
34. Georgia Southern University Jiann-Ping Hsu College of Public Health: Degrees & Programs. https://jphcoph.georgiasouthern.edu/degrees/#DrPHDegree (2019). Accessed August 12 2019.
35. Georgia Southern University Jiann-Ping Hsu College of Public Health: Catalog 2019-2020 - Doctor of Public Health. https://catalog.georgiasouthern.edu/graduate/jiann-ping-hsu-public-health/doctor-public-health/ (2019). Accessed August 12 2019.
36. Georgia State University School of Public Health: Doctor of Public Health - School of Public Health. https://publichealth.gsu.edu/academics-student-life/degrees-programs/drph/ (2019). Accessed August 12 2019.
37. Georgia State University School of Public Health: Doctor of Public Health - Frequently Asked Questions. https://publichealth.gsu.edu/academics-student-life/degrees-programs/drph-faq/ (2019). Accessed August 12 2019.
38. Georgia State University School of Public Health: Doctor of Public Health - How to Apply. https://publichealth.gsu.edu/academics-student-life/degrees-programs/drph-apply/ (2019). Accessed August 12 2019.
39. Harvard T.H. Chan School of Public Health: Doctor of Public Health (DrPH) Student Information. updated 2014-07-24. https://www.hsph.harvard.edu/student-handbook/doctor-of-public-health-drph-student-information/ (2014). Accessed August 12 2019.

40. Harvard T.H. Chan School of Public Health: DrPH DELTA Doctoral Project Manual Class of 2019. https://cdn1.sph.harvard.edu/wp-content/uploads/sites/1496/1496/20/DrPH-Delta-Doctoral-Project-Manual-Cohort-3-1.pdf (2018). Accessed August 12 2019.
41. Harvard T.H. Chan School of Public Health: DrPH Program Student Manual - For students entering July 2019. https://cdn1.sph.harvard.edu/wp-content/uploads/sites/1496/2019/09/DrPH-Student-Manual-for-Class-of-2022.pdf (2018). Accessed August 12 2019.
42. Harvard T.H. Chan School of Public Health: The Harvard DrPH. https://www.hsph.harvard.edu/drph/ (2019). Accessed August 12 2019.
43. Jackson State University School of Public Health: Doctor of Public Health Degree Admission Requirements. http://www.jsums.edu/health/files/2018/05/DrPH-Admission-Requirements-1.pdf (2019). Accessed August 13 2019.
44. Jackson State University School of Public Health: School of Public Health Doctor of Public Health Degree Student Handbook,. http://www.jsums.edu/health/files/2017/12/DrPHStudentHandbook-2017-2019.pdf?x17560 (2017). Accessed August 13 2019.
45. Loma Linda University: Student Handbook 2019-2020. https://home.llu.edu/sites/home.llu.edu/files/docs/student-handbook.pdf (2019). Accessed August 13 2019.
46. Loma Linda University School of Public Health: Doctoral Programs. https://publichealth.llu.edu/academics/drph (2019). Accessed August 13 2019.
47. Loma Linda University School of Public Health: Doctoral Degrees. http://llucatalog.llu.edu/public-health/doctoral-degrees/doctoral-degrees.pdf (2019). Accessed August 13 2019.
48. Loma Linda University School of Public Health: Health Education — Dr.P.H. http://llucatalog.llu.edu/public-health/health-education-drph/#text (2019). Accessed August 13 2019.
49. Loma Linda University School of Public Health: Health Policy and Leadership — Dr.P.H. http://llucatalog.llu.edu/public-health/health-policy-leadership-drph/#text (2019). Accessed August 13 2019.
50. Loma Linda University School of Public Health: Preventive Care — Dr.P.H. (2019). Accessed August 13 2019.
51. New York Medical College School of Health Sciences and Practice & Institute of Public Health: Doctor of Public Health (Dr.P.H.). http://www.nymc.edu/school-of-health-sciences-and-practice-shsp/shsp-academics/degrees/doctor-of-public-health-drph/ (2019). Accessed August 13 2019.
52. Pennsylvania State University College of Medicine Public Health Program: DrPH Doctor of Public Health Program. https://med.psu.edu/drph (2019). Accessed August 13 2019.
53. Pennsylvania State University College of Medicine Public Health Program: 2019-20 Doctor of Public Health Handbook. https://students.med.psu.edu/doctor-of-public-health-drph/handbook/ (2019). Accessed August 13 2019.
54. SUNY Downstate Medical Center School of Public Health: Doctor of Public Health - Course Descriptions. https://www.downstate.edu/publichealth/programs/doctor-of-public-health.html (2019). Accessed August 13 2019.

55. SUNY Downstate Medical Center School of Public Health: 2019-2020 Student Handbook. https://sls.downstate.edu/student_affairs/_documents/student_handbooks/student_handbook_2019-20.pdf (2019). Accessed August 13 2019.
56. Texas A&M School of Public Health: Doctor of Public Health (DrPH). https://sph.tamhsc.edu/degrees/drph/index.html (2019). Accessed August 13 2019.
57. Texas A&M School of Public Health: Doctor of Public Health in Public Health Sciences. https://catalog.tamu.edu/graduate/colleges-schools-interdisciplinary/public-health/interdepartmental/public-health-sciences-drph/#text (2019). Accessed August 13 2019.
58. Tulane University School of Public Health and Tropical Medicine: DrPH in Global Community Health and Behavioral Sciences. https://sph.tulane.edu/gchb/drph (2019). Accessed August 13 2019.
59. University at Albany School of Public Health: Public Health Doctor of Public Health Degree Program (DrPH) - University at Albany-SUNY. https://www.albany.edu/graduatebulletin/public_health_drph_degree.htm (2019). Accessed August 13 2019.
60. University at Albany School of Public Health: Graduate Student Handbook 2017-2018. https://www.albany.edu/sph/assets/2017-2018_Graduate_Handbook_FINAL.pdf (2019). Accessed August 13 2019.
61. University of Alabama at Birmingham School of Public Health: Department of Biostatistics Graduate Student Handbook 2019-2020. https://www.soph.uab.edu/files/Student%20Handbooks/2019/BST_Graduate_Handbook_2019.pdf (2019). Accessed September 30 2019.
62. University of Alabama at Birmingham School of Public Health: Graduate Education. https://www.soph.uab.edu/graduate (2019). Accessed August 13 2019.
63. University of Arizona Mel and Enid Zuckerman College of Public Health: Doctor of Public Health (DrPH) in Maternal & Child Health | Mel and Enid Zuckerman College of Public Health. updated 2014-07-30T16:24-07:00. https://publichealth.arizona.edu/academics/doctoral-programs/drph-in-mch (2014). Accessed August 13 2019.
64. University of Arizona Mel and Enid Zuckerman College of Public Health: Doctor of Public Health (DrPH) in Public Health Policy and Management | Mel and Enid Zuckerman College of Public Health. updated 2014-07-30T16:10-07:00. https://publichealth.arizona.edu/academics/doctoral-programs/drph-in-phpm (2014). Accessed August 13 2019.
65. University of Arizona Mel and Enid Zuckerman College of Public Health: New Student Guide. https://publichealth.arizona.edu/sites/publichealth.arizona.edu/files/students/pdfs/New%20Student%20Guide%202018.pdf (2018). Accessed August 13 2019.
66. University of Arizona Mel and Enid Zuckerman College of Public Health: 2019-2020 DrPH-MCH Program Student Handbook. https://publichealth.arizona.edu/sites/publichealth.arizona.edu/files/MCH%202019-20%20Handbook.pdf (2019). Accessed September 30 2019.

67. University of Arkansas for Medical Sciences Fay W. Boozman College of Public Health: Doctor of Public Health in Public Health Leadership - Fay W. Boozman College of Public Health. https://publichealth.uams.edu/academics/doctoral/drph/ (2019). Accessed August 13 2019.
68. University of California Berkeley School of Public Health: Doctor of Public Health. updated 2013-05-24. https://sph.berkeley.edu/areas-study/doctor-public-health (2013). Accessed August 13 2019.
69. University of California Berkeley School of Public Health: 2019-20 DrPH Student Handbook. https://publichealth.berkeley.edu/wp-content/uploads/2019/08/2019-20_DrPH-Handbook.pdf (2019). Accessed September 30 2019.
70. University of Georgia College of Public Health: DrPH Residency Student Manual. publichealth.uga.edu/.../Doctor_of_Public_Health_Residency_Handbook_2019-2020 (2019). Accessed August 13 2019.
71. University of Georgia College of Public Health: Doctor of Public Health (DrPH) - College of Public Health UGA. https://publichealth.uga.edu/degree/doctor-of-public-health-drph/ (2019). Accessed August 13 2019.
72. University of Illinois at Chicago School of Public Health: Doctor of Public Health Leadership. https://publichealth.uic.edu/academics/public-health-degrees/drph/ (2019). Accessed December 30 2019.
73. University of Illinois at Chicago School of Public Health: FAQs. https://publichealth.uic.edu/academics/public-health-degrees/doctor-public-health-leadership/faqs/ (2019). Accessed August 13 2019.
74. University of Illinois at Chicago School of Public Health: 2019-20 The Doctor of Public Health Program Graduate Student Handbook. https://apps.sph.uic.edu/webdocs/pdf/shandbooks/DrPH_Student_Handbook_2019_2020_Final.pdf (2019). Accessed September 30 2019.
75. University of North Carolina Gillings School of Global Public Health: Doctoral Program in Health Leadership (DrPH) - Academic Policies, Guidelines, and Procedures. http://hpmadmittedstudents.web.unc.edu/files/2018/10/DrPH_Handbook_latest.pdf (2018). Accessed August 13 2019.
76. University of North Carolina Gillings School of Global Public Health: Programs Archive. https://sph.unc.edu/programs/ (2019). Accessed August 13 2019.
77. University of Puerto Rico Graduate School of Public Health: Doctorate in Public Health with Specialization in Health Systems Analysis and Management (DrPH HSAM). http://sp.rcm.upr.edu/asuntos-academicos/programas-academicos/doctorado-en-salud-publica-con-especialidad-en-analisis-de-sistemas-de-salud-y-gerencia-drph-hsam/ (2019). Accessed August 13 2019.
78. University of Puerto Rico Graduate School of Public Health: Doctorate in Public Health (DrPH) with Specialization in Environmental Health. http://sp.rcm.upr.edu/asuntos-academicos/programas-academicos/doctorado-en-salud-publica-drph-con-especialidad-en-salud-ambiental/ (2019). Accessed August 13 2019.
79. University of Puerto Rico Graduate School of Public Health: Doctorate in Public Health with Specialization in Social Determinants of Health. http://sp.rcm.upr.edu/asuntos-academicos/programas-academicos/doctorado-en-salud-publica-drph-con-especialidad-en-determinantes-sociales-de-la-salud/ (2019). Accessed August 13 2019.

80. University of Puerto Rico: Medical Sciences Campus Catalog 2017-2020. http://www.rcm.upr.edu/wp-content/uploads/sites/3/2019/01/UPR-MSC-CATALOG-2017-2020-REV-01092019.pdf (2017). Accessed August 13 2019.
81. University of South Florida College of Public Health: Doctor of Public Health Program (DrPH) Student Manual. https://health.usf.edu/-/media/Files/Public-Health/Global-Health/DrPHStudentManual11011.ashx (2011). Accessed August 13 2019.
82. University of South Florida College of Public Health: Doctor of Public Health (DrPH). https://health.usf.edu/publichealth/apply/graduate-admissions/drph (2019). Accessed August 13 2019.
83. University of Texas Health Science Center at Houston School of Public Health: General Information Catalog 2018–2020. https://www.uth.edu/academics/docs/school-catalogs/2018-2020-General-Information-FINAL.pdf (2018). Accessed August 13 2019.
84. University of Texas Health Science Center at Houston School of Public Health: Doctor of Public Health (DrPH). https://sph.uth.edu/academics/degree-programs/doctor-of-public-health-drph/ (2019). Accessed August 13 2019.
