# Supplementary material for: Analysis of CEPH-accredited DrPH programs in the United States: A mixed-methods study
Source: PLoS One. 2021 Feb 4;16(2):e0245892. doi: 10.1371/journal.pone.0245892 (PMC7861440; doi:10.1371/journal.pone.0245892)
Supplement: S7 Table — (PDF) [file pone.0245892.s007.pdf]

S7 Table. Required leadership and finance courses

School Name	Course Name	Course Description
1. Boston University School of Public Health [1]	▪ PH 866 Public Health Leadership Seminar (0 credits)<br>▪ Management and Finance electives (4 credits)	▪ PH 866: Non-credit course for three semesters (Year 1 Fall, Spring, Summer, Year 2 Fall). It provides 1) the main integrative pedagogic experience of the DrPH Program, 2) a place for combining and applying knowledge from previous courses and field experience, 3) address of crosscutting issues that were not covered in other courses, 4) opportunity to meet and interact with senior public health officials or practitioners.<br>▪ Management and Finance electives: student should select four credits of public health management and/or finance course.
2. Claremont Graduate University School of Community & Global Health [2]	▪ CGH 316 Public Health Leadership (4 credits)<br>▪ CGH 318 Management of International Health Programs and Organizations	N/A
3. University of Colorado School of Public Health [3]	▪ CBHS/EPID/EHOH 7020 DrPH Seminar in Leadership (3 credits)<br>▪ HSMP 7605 Managing a Learning Healthcare System (3 credits)	▪ CBHS/EPID/EHOH 7020: It provides 1) a foundation of emerging public health leaders and 2) address central leadership topics.
4. Columbia University Mailman School of Public Health [4-6]	▪ DrPH in Biostatistics: P9070 DrPH Case Studies in Public Health Leadership I & II (1.5 credits)<br>▪ DrPH in Environmental Health Sciences: N/A<br>▪ DrPH in Epidemiology: N/A<br>▪ DrPH in Leadership in Global Health and Humanitarian Systems: Science, Practice & Leadership in Public Health (3 credits), Program selective - 1.3-3 credits required, among P8601 Public Health Program Planning (3 credits), P8640 Methods in Program	▪ DrPH in Sociomedical Sciences: P9070 Case Studies in Public Health Leadership is provided in fall (1.5 credits) and spring semester (1.5 credits)

School Name	Course Name	Course Description
	Evaluation (3 credits), P9620 Applications of Implementation Science in Low & Middle Income Countries (1.5 credits)<br>▪ DrPH in Sociomedical Sciences: P9070 Case Studies in Public Health Leadership (3 credits)	
5. Drexel University Dornsife School of Public Health [7, 8]	▪ HMP 500 Health Management and Policy I (3 credits)<br>▪ HMP 501 Health Management and Policy II (3 credits)<br>▪ HMP 512 The Business of Healthcare: Advanced Healthcare Financial Management (3 credits)<br>▪ HMP 515 Health Organizational Leadership (3 credits)<br>▪ HMP 556 Public Health Leadership (3 credits)	▪ HMP 500 & 501: It focuses on broad-based understanding of the public health and health care systems in the U.S., including their relationship and interactions.<br>▪ HMP 512: It is designed for non-financial health care managers, introducing the most-used tools and techniques of health care financial management<br>▪ HMP 515: It focuses on leadership in the health industry from three perspectives: individual leadership styles, common elements of effective teams, and organizational dynamics.<br>▪ HMP 556: It explores today's fields of public health and health care challenges leaders.
6. East Tennessee State University College of Public Health [9]	▪ HSMP 6130 Public Health Leadership, Policy Development, and Ethics (3 credits)<br>▪ HSMP 6310 Population Health Management<br>▪ HSMP 6340 Leadership, Management, & Governance in Public Health (3 credits)	▪ HSMP 6130: It is core DrPH course.<br>▪ HSMP 6310 & 6340: It is for Health Management and Policy Concentration.
7. George Washington University Milken Institute School of Public Health [10-14]	▪ PUBH 8401 Foundations of Public Health Leadership and Practice – Doctoral Seminar (3 credits)<br>▪ PUBH 8402 Leadership and Decision Making: A Skills Based Approach (2 credits)<br>▪ PUBH 8403 Leadership in Public Health Practice and Policy (2 credits)	▪ PUBH 8401: It is an interactive seminar course providing a fundamental understanding of the history and current issues of health policy, health behavior, global health, and environmental and occupational health.<br>▪ PUBH 8402: It focuses on how to solve complex health problems and implement successful solutions to improve population health using leadership and decision-making skills.

School Name	Course Name	Course Description
		▪ PUBH 8403: Students work for clients from public health-related agencies or organization in the Washington DC area.
8. Georgia Southern University Jiann-Ping Hsu College of Public Health [15]	▪ PHLD 9131 Leadership Foundations and Strategies for Health Organizations (3 credits)<br>▪ PHLD 9334 Financial Management of Public Health Organizations and Programs (3 credits)	▪ PHLD 9334: It is for DrPH in Public Health Leadership.
9. Georgia State University School of Public Health [16]	▪ PH 9005 Doctoral Seminar: Public Health Policy, Analysis, and Advocacy for Leaders (3 credits)<br>▪ PH 9300 Public Health Leadership and Practice (3 credits)<br>▪ PH 9310 Public Health Management and Finance (3 credits)	N/A
10. Harvard T.H. Chan School of Public Health [17-19]	▪ DRPH 290A Integrating (DELTA) Seminar (1.25 credits)<br>▪ DRPH 290B Integrating (DELTA) Seminar Year 2 (1.25 credits)<br>▪ HPM 219 Financial Transactions and Analysis (2.5 credits)<br>▪ HPM 220 Financial Management and Control (2.5 credits)	▪ HPM 219: It introduces concepts of financial accounting for the non-accountant user of financial information.<br>▪ HPM 220: It focuses on cost accounting, management control concepts, and uses for health service organizations.
11. Johns Hopkins Bloomberg School of Public Health [20]	▪ DrPH First-year Monthly Seminar (non-credit)<br>▪ 2nd Year Schoolwide DrPH Seminar (2 credits)	N/A
12. Loma Linda University School of Public Health [21-26]	▪ PHCJ 607 Professional Leadership (3 credits)<br>▪ PHCJ 616 Administrative Systems in Agency Management (3 credits)<br>▪ PHCJ 617 Building Healthy Systems (3 credits)	▪ PHCJ 607: It is an applied course that exposes students to leadership styles and applications within public health and health-care settings, and in which students explore and develop their personal leadership attributes.<br>▪ PHCJ 616: It reviews the administrative systems and knowledge necessary to manage public health, health-care, and other agencies.

School Name	Course Name	Course Description
		▪ PHCJ 617: It develops advanced public health leadership in building sustainable health systems.
13. New York Medical College School of Health Sciences and Practice & Institute of Public Health [27, 28]	▪ HPMM 8014 Public Health Leadership (4 credits)	▪ HPMM 8014: It is to introduce students to theories and concepts of leadership, provides students the opportunity to identify their personal leadership attributes, and through case study development and analysis, review leadership challenges from public health practice.
14. Pennsylvania State University College of Medicine Public Health Program [29]	▪ PHS 575 Integrative Public Health Leadership (3 credits)	▪ PHS 575: It examines the dynamic nature of leadership in the public health sector and introduce major theories and concepts of leadership and ways to apply these to public health issues requiring leadership
15. SUNY Downstate Medical Center School of Public Health [30]	▪ HPMG 7200 Public Health Management and Ethics (3 credits)<br>▪ CHSC 7203 Program Evaluation: Theory, Practice, and Research (3 credits)<br>▪ EOHS 7202 Advanced Topics in Risk Assessment and Management (3 credits)	▪ HPMG 7200: It emphasizes cross-disciplinary approaches to addressing and resolving public health problems through the development of key management and leadership skills.<br>▪ CHSC 7203: It is Community Health Sciences Core course. It Examines the planning of evaluation, construction of instruments and strategies of measurement, and methods of effective data collection, management, and analysis.<br>▪ EOHS 7202: It is Environmental & Occupational Health Sciences Core course. It reviews the core foundations of risk assessment including hazard identification, dose response, exposure assessment, and risk characterization and provides practical experience in the study of risk management.
16. Texas A&M School of Public Health	▪ Leadership courses from the Bush School of Government and Public Service<br>▪ Taught by rotating faculty within the department	N/A

School Name	Course Name	Course Description
17. Tulane University School of Public Health and Tropical Medicine [31, 32]	▪ Advanced Monitoring and Evaluation, choose 1 from the following three courses (3 credits): GCHB 7070 Social Impact of HIV/AIDS; GCHB 7120 Monitoring and Evaluation of Maternal Child Health; GCHB 7210 Survey Data for Family Planning/Reproductive Health	▪ GCHB 7070: It critically reflects on current strategies to stem the epidemic through a sociological approach to understanding epidemiological patterns.<br>▪ GCHB 7210: It introduces a number of key concepts and measures used in the monitoring and evaluation of family planning and reproductive health programs.
18. University at Albany School of Public Health [33]	▪ Student should select one of the following (3 credits): Management (e.g., HPM 641-approval of advisor), Leadership (e.g., SPH 569-approval of advisor), Policy (e.g., HPM 501-approval of advisor)<br>▪ HPM 647 Program Evaluation (3 credits)<br>▪ HPM 650 Strategy and Leadership Applications in Health Management (3 credits)	▪ HPM 647, 650: it is Health Policy, Management and Behavior Concentration courses.
19. University of Alabama at Birmingham School of Public Health [34, 35]	▪ HCO 716 Advanced Leadership and Practice Seminar (3 credits)<br>▪ HCO 718 Management Concepts in Public Health Programs (3 credits)<br>▪ HCO 706 Strategic Management & Theory (3 credits)<br>▪ HCO 715 Finance for Health Professionals (3 credits)<br>▪ HCO 718 Management Concepts in Public Health Programs (3 credits)	▪ HCO 718: It is Maternal & Child Health Policy DrPH Concentration core course.<br>▪ HCO 706, 715, 718: They are Health Care Organization and Policy Core courses.
20. University of Arizona Mel and Enid Zuckerman College of Public Health [36-39]	▪ PHPM 569 Fundamentals of Health Budgeting and Financial Management (3 credits)<br>▪ HPS 544 Fundamentals of Evaluation (3 credits)<br>▪ HPS 704 Public Health Leadership to Reduce Health Disparities (3 credits)	▪ PHPM 569: It offers a current approach to the fundamentals of budgeting and financial management with an emphasis on non-profit and health care organizations, in particular the community health sector.<br>▪ HPS 544: It serves as an introduction to those evaluation tools most commonly used to assess the performance of public health programs,

School Name	Course Name	Course Description
		including implementation and process assessment, methods of outcome evaluation, and evaluation of public health initiatives such as community coalition and advocacy work.<br>▪ HPS 704: It aims to advance leadership development knowledge, attitudes, and practice among doctorate of public health students to advance public health policy and systems change efforts.
21. University of Arkansas for Medical Sciences Fay W. Boozman College of Public Health [40, 41]	▪ HPMT 6114 Advanced Health Policy and Management (3 credits)<br>▪ HPMT 5114 Management of Health Care Organizations (3 credits)<br>▪ HPMT 5124 Strategic Planning (3 credits)<br>▪ HBHE 6436 Communication for Public Health Leaders (3 credits)	▪ HPMT 6114: It provides an advanced examination of issues related to the development, implementation, and impact of public policies and health system management strategies on population health.<br>▪ HPMT 5114: It focuses on the fundamental management issues and techniques that can be used to administer a health care organization.<br>▪ HPMT 5124: It focuses on the major types of health planning in the U.S. including related supply regulation.<br>▪ HBHE 6436: It focuses on theoretical overview of organizational communication, including communication flow, networks, organizational relationships, groups, conflict, and language.
22. University of California Berkeley School of Public Health	N/A	N/A
23. University of Georgia College of Public Health [42, 43]	▪ HPAM 9100 Doctor of Public Health Seminar I (3 credits)<br>▪ HPAM 9200 Doctor of Public Health Seminar II (3 credits)<br>▪ HPAM 8700 Management of Healthcare Organizations (3 credits)<br>▪ HPAM 8450 Policy Evaluation (3 credits)	▪ HPAM 9100, 9200: They address to link theory and practice in data and analysis, leadership, management and governance, policy and programs, and education and workforce development in public health.<br>▪ HPAM 8700: It stimulates critical thinking about modern public health administrative issues,

School Name	Course Name	Course Description
	▪ HPAM 8800 Public Health Leadership (3 credits)	addresses MPH core competencies, and develops selected management techniques and perspectives.<br>▪ HPAM 8450: It provides a survey of standard quantitative and qualitative techniques for prospectively analyzing health policy issues<br>▪ HPAM 8800: It focuses on the development of leadership and managerial competencies relevant for work in public and private health care institutions.
24. University of Illinois at Chicago School of Public Health [44, 45]	▪ IPHS 501 Public Health Leadership Seminar 1 (3 credits)<br>▪ IPHS 502 Public Health Leadership Seminar 2 (3 credits)<br>▪ IPHS 510 Leadership in Public Health Policy Development (3 credits)<br>▪ IPHS 511 Personal Leadership Development (3 credits)<br>▪ IPHS 512 Public Health Leadership Tools (3 credits)	▪ IPHS 501: It is intended to build organizational and systems leadership skills for public health professionals who are expected to be in leadership positions at the highest levels within the public health system.<br>▪ IPHS 502: It applies strategic management as a leadership tool to drive change and foster innovation within public health organizations and public health systems.<br>▪ IPHS 510: It covers the policy process and role of policy analysis in policy development from a leadership perspective.<br>▪ IPHS 511: It examines the personal dimensions of leadership and is intended to give students a basis for understanding their leadership styles, those of others, and to further professional leadership development.<br>▪ IPHS 512: It covers some of the most commonly used and practical leadership process management tools from both a theoretical and applied perspective.
25. University of North Carolina Gillings School of Global Public Health [46]	▪ HPM 963 Program Evaluation for Health Leaders	▪ HPM 963: It reviews fundamental evaluation frameworks, and helps students identify

School Name	Course Name	Course Description
		appropriate evaluation and research design and data collection strategies.
26. University of Puerto Rico Graduate School of Public Health [47-49]	Common:<br>▪ ADSS 8005 Organizational and Administrative Elements in Health Care (3 credits)<br>For DrPH in Environmental Health:<br>▪ SAAM 8017 Health Risk Assessment (3 credits)<br>▪ SALP 8020 Public Health Leadership Seminar (1 credit)<br>For DrPH in Health Systems Analysis and Management:<br>▪ ADSS 8009 Quality and Results Management in Health Systems (3 credits)<br>▪ ADSS 8205 Financial Management of Health Systems (3 credits)<br>▪ ADSS 8008 Health Systems Planning and Strategic Management (3 credits)<br>▪ ADSS 8307 Health Systems Evaluation (3 credits)<br>▪ ADSS 8206 Economic Analysis of Health Systems Management (3 credits)<br>▪ ADSS 8105 Leadership in Public Health (2 credits)<br>For DrPH in Social Determinants of Health:<br>▪ DESS 8206 Foundation and Community Organization in the Social Determinants of Health (3 credits)	N/A
27. University of South Florida College of Public Health [50, 51]	PHC 7149 Practical Applications II: Public Health Leadership (1 credit)	N/A

School Name	Course Name	Course Description
28. University of Texas Health Science Center at Houston School of Public Health [52]	▪ PHD 1113 Advanced Methods for Planning and Implementing Health Promotion Programs (Intervention Mapping) (3 credits)<br>▪ PHD 1120 Program Evaluation (3 credits)<br>▪ PHD 3950 Advanced Leadership Studies in Public Health (3 credits)	N/A

References

1. Boston University School of Public Health: DrPH Program Guidelines 2018-2019. https://www.bu.edu/sph/files/2018/10/DrPH-Handbook-2018.pdf (2018). Accessed August 12 2019.
2. Claremont Graduate University School of Community & Global Health: Archived Bulletin - Public Health, DrPH. http://bulletin.cgu.edu/preview_program.php?catoid=13&poid=1646&returnto=1595 (2019). Accessed August 12 2019.
3. Colorado School of Public Health: Doctor of Public Health 2019-2020 Student Handbook. http://www.ucdenver.edu/academics/colleges/PublicHealth/resourcesfor/currentstudents/academics/Documents/19_20_Handbooks/DRPH_Handbook_19_20.pdf (2019). Accessed September 30 2019.
4. Columbia University Mailman School of Public Health: The Department of Biostatistics Student Handbook 2019-2020. https://www.mailman.columbia.edu/sites/default/files/biostats_student_handbook_2019-2020.pdf (2018). Accessed September 30 2019.
5. Columbia University Mailman School of Public Health: Department of Sociomedical Sciences Doctoral Student Handbook 2019-2020. https://www.mailman.columbia.edu/sites/default/files/pdf/sms-doctoral-handbook-2019-20.pdf (2019). Accessed September 30 2019.
6. Columbia University Mailman School of Public Health: Heilbrunn Department of Population & Family Health Doctoral Program Handbook Version 4.1 (March 2018). https://www.mailman.columbia.edu/sites/default/files/pdf/pfh-drph-handbook-march-2018.pdf (2019). Accessed January 8 2020.
7. Drexel University: Search Results: HMP 556 Public Health Leadership 3.0 Credits. http://catalog.drexel.edu/search/?P=HMP%20556 (2019). Accessed October 1 2019.
8. Drexel University: Search Results: HMP 515 Health Organizational Leadership 3.0 Credits. (2019). Accessed October 1 2019.
9. East Tennessee State University College of Public Health: Graduate Health Professions Education Doctor of Public Health 2019-2020 Student Handbook. https://www.etsu.edu/cph/documents/drphhandbook.pdf (2018). Accessed January 9 2020.
10. George Washington University Milken Institute School of Public Health: Program Guide - Doctor of Public Health, Environmental and Occupational Health. https://publichealth.gwu.edu/sites/default/files/DrPH%20EOH%202018%20%28May%202018%29.pdf (2018). Accessed August 12 2019.
11. George Washington University Milken Institute School of Public Health: Program Guide - Doctor of Public Health, Global Health. https://publichealth.gwu.edu/sites/default/files/DrPH%20Global%20Health%202018_0.pdf (2018). Accessed August 12 2019.
12. George Washington University Milken Institute School of Public Health: Program Guide - Doctor of Public Health, Health Behavior. https://publichealth.gwu.edu/sites/default/files/DrPH%20HB%202018_0.pdf (2018). Accessed August 12 2019.

13. George Washington University Milken Institute School of Public Health: Program Guide - Doctor of Public Health, Health Policy. https://publichealth.gwu.edu/sites/default/files/DrPH%20Health%20Policy%202018_0.pdf (2018). Accessed August 12 2019.
14. George Washington University Milken Institute School of Public Health: Public Health (PUBH) - Explanation of Course Numbers. http://bulletin.gwu.edu/courses/pubh/ (2019). Accessed December 30 2019.
15. Georgia Southern University Jiann-Ping Hsu College of Public Health: Plans of Study & Program Maps. https://jphcoph.georgiasouthern.edu/degrees/plans-of-study/ (2019). Accessed October 3 2019.
16. Georgia State University School of Public Health: Doctor of Public Health - Curriculum. https://publichealth.gsu.edu/academics-student-life/degrees-programs/drph-curriculum/ (2019). Accessed October 3 2019.
17. Harvard T.H. Chan School of Public Health: DrPH Program Student Manual - For students entering July 2019. https://cdn1.sph.harvard.edu/wp-content/uploads/sites/1496/2019/09/DrPH-Student-Manual-for-Class-of-2022.pdf (2018). Accessed August 12 2019.
18. Coursicle: HPM 220 - Financial Management and Control at Harvard University | Coursicle Harvard. https://www.coursicle.com/harvard/courses/HPM/220/ (2019). Accessed October 3 2019.
19. Coursicle: HPM 219 - Financial Transactions and Analysis at Harvard University | Coursicle Harvard. @coursicle. https://www.coursicle.com/harvard/courses/HPM/219/ (2019). Accessed October 3 2019.
20. Johns Hopkins Bloomberg School of Public Health: Program Curriculum. https://www.jhsph.edu/academics/degree-programs/doctoral-programs/doctor-of-public-health/Curriculum.html (2019). Accessed August 13 2019.
21. Loma Linda University School of Public Health: Health Education — Dr.P.H. http://llucatalog.llu.edu/public-health/health-education-drph/#text (2019). Accessed August 13 2019.
22. Loma Linda University School of Public Health: Health Policy and Leadership — Dr.P.H. http://llucatalog.llu.edu/public-health/health-policy-leadership-drph/#text (2019). Accessed August 13 2019.
23. Loma Linda University School of Public Health: Preventive Care — Dr.P.H. (2019). Accessed August 13 2019.
24. Loma Linda University: Search Results - PHCJ 607. Professional Leadership. 3 Units. http://llucatalog.llu.edu/search/?P=PHCJ%20607 (2019). Accessed October 3 2019.
25. Loma Linda University: Search Results - PHCJ 616. Administrative Systems in Agency Management. 3 Units. http://llucatalog.llu.edu/search/?P=PHCJ%20616 (2019). Accessed October 3 2019.
26. Loma Linda University: Search Results - PHCJ 617. Building Healthy Systems. 3 Units. http://llucatalog.llu.edu/search/?P=PHCJ%20617 (2019). Accessed October 3 2019.
27. New York Medical College School of Health Sciences and Practice & Institute of Public Health: Curriculum. @wearetouro. http://www.nymc.edu/school-of-health-sciences-and-practice-shsp/shsp-academics/degrees/doctor-of-public-health-drph/curriculum/ (2019). Accessed October 3 2019.

28. New York Medical College School of Health Sciences and Practice & Institute of Public Health: Course Description - HPMM 8014 Public Health Leadership (Fall and Spring). https://www.nymc.edu/academics/course-descriptions/school-of-health-sciences-and-practice-shsp/health-policy-and-management/hpmm-8014-public-health-leadership.php (2019). Accessed October 3 2019.
29. Pennsylvania State University College of Medicine Public Health Program: 2019-20 Doctor of Public Health Handbook. https://students.med.psu.edu/doctor-of-public-health-drph/handbook/ (2019). Accessed August 13 2019.
30. SUNY Downstate Medical Center School of Public Health: Doctor of Public Health - Course Descriptions. https://www.downstate.edu/publichealth/programs/doctor-of-public-health.html (2019). Accessed August 13 2019.
31. Tulane University School of Public Health and Tropical Medicine: DrPH in Global Community Health and Behavioral Sciences. https://sph.tulane.edu/gchb/drph (2019). Accessed August 13 2019.
32. Tulane University School of Public Health and Tropical Medicine: Course Descriptions - Global Comm Hlth Sci & Beh (GCHB). https://catalog.tulane.edu/courses/gchb/ (2019). Accessed October 4 2019.
33. University at Albany School of Public Health: Public Health Doctor of Public Health Degree Program (DrPH) - University at Albany-SUNY. https://www.albany.edu/graduatebulletin/public_health_drph_degree.htm (2019). Accessed August 13 2019.
34. University of Alabama at Birmingham School of Public Health: Department of Biostatistics Graduate Student Handbook 2019-2020. https://www.soph.uab.edu/files/Student%20Handbooks/2019/BST_Graduate_Handbook_2019.pdf (2019). Accessed September 30 2019.
35. University of Alabama at Birmingham School of Public Health: Doctor of Public Health - Program Description. https://www.soph.uab.edu/doctor-public-health-1 (2019). Accessed October 3 2019.
36. University of Arizona Mel and Enid Zuckerman College of Public Health: 2019-2020 DrPH-MCH Program Student Handbook. https://publichealth.arizona.edu/sites/publichealth.arizona.edu/files/MCH%202019-20%20Handbook.pdf (2019). Accessed September 30 2019.
37. University of Arizona Mel and Enid Zuckerman College of Public Health: Syllabus, Fall 2019 PHPM 569: Fundamentals of Health Budgeting and Financial Management. https://publichealth.arizona.edu/sites/publichealth.arizona.edu/files/academics/Syllabi/PHPM%20569%20Fundamentals%20of%20Health%20Budgeting%20and%20Financial%20Management_2019_Fall.pdf (2019). Accessed October 3 2019.
38. University of Arizona Mel and Enid Zuckerman College of Public Health: Syllabus, Spring 2019 HPS 544: Applied Aspects of Program Planning, Implementation & Evaluation. https://publichealth.arizona.edu/sites/publichealth.arizona.edu/files/academics/Syllabi/HPS%20544%20Applied%20Evaluation%20of%20Public%20Health%20Programs_2019_Spring.pdf (2019). Accessed October 3 2019.
39. University of Arizona Mel and Enid Zuckerman College of Public Health: Syllabus, Spring 2019 HPS 704: Doctoral Public Health Leadership for Health Equity.

https://publichealth.arizona.edu/sites/publichealth.arizona.edu/files/academics/Syllabi/HPS%20704%20Doctoral%20Leadership%20for%20Health%20Equity_2019_Spring.pdf (2019). Accessed October 3 2019.
40. University of Arkansas for Medical Sciences Fay W. Boozman College of Public Health: Doctor of Public Health Degree Track Planner. http://publichealth.uams.edu/wp-content/uploads/sites/3/2012/07/DrPH-Doctor-in-Public-Health-Leadership-Degree-Track-Planner-8.1.2017.pdf (2019). Accessed October 4 2019.
41. University of Arkansas for Medical Sciences Fay W. Boozman College of Public Health: Course Catalog and Student Handbook. https://secure.uams.edu/cophstudent/courses.aspx (2019). Accessed October 4 2019.
42. University of Georgia College of Public Health: Doctor of Public Health (DrPH) - College of Public Health UGA. https://publichealth.uga.edu/degree/doctor-of-public-health-drph/ (2019). Accessed August 13 2019.
43. University of Georgia College of Public Health: UGA Bulletin - Courses. http://bulletin.uga.edu/CoursesHome.aspx?Prefix=HPAM (2019). Accessed October 4 2019.
44. University of Illinois at Chicago School of Public Health: 2019-20 The Doctor of Public Health Program Graduate Student Handbook. https://apps.sph.uic.edu/webdocs/pdf/shandbooks/DrPH_Student_Handbook_2019_2020_Final.pdf (2019). Accessed September 30 2019.
45. University of Illinois at Chicago School of Public Health: Interdisciplinary Public Health Sciences (IPHS) - Courses. https://catalog.uic.edu/gcat/course-descriptions/iphs/ (2019). Accessed October 4 2019.
46. University of North Carolina Gillings School of Global Public Health: DrPH Curriculum and Degree Requirements. https://sph.unc.edu/hpm/hpm-drph-curriculum/ (2019). Accessed October 4 2019.
47. University of Puerto Rico Graduate School of Public Health: Doctorate in Public Health with Specialization in Health Systems Analysis and Management (DrPH HSAM). http://sp.rcm.upr.edu/asuntos-academicos/programas-academicos/doctorado-en-salud-publica-con-especialidad-en-analisis-de-sistemas-de-salud-y-gerencia-drph-hsam/ (2019). Accessed August 13 2019.
48. University of Puerto Rico Graduate School of Public Health: Doctorate in Public Health (DrPH) with Specialization in Environmental Health. http://sp.rcm.upr.edu/asuntos-academicos/programas-academicos/doctorado-en-salud-publica-drph-con-especialidad-en-salud-ambiental/ (2019). Accessed August 13 2019.
49. University of Puerto Rico Graduate School of Public Health: Doctorate in Public Health with Specialization in Social Determinants of Health. http://sp.rcm.upr.edu/asuntos-academicos/programas-academicos/doctorado-en-salud-publica-drph-con-especialidad-en-determinantes-sociales-de-la-salud/ (2019). Accessed August 13 2019.
50. University of South Florida College of Public Health: DrPH 2019-2020Plan of StudyConcentration in Advanced Practice Leadership in Public Health. https://usf.app.box.com/s/8u7h2fy7v37luov6fb3598blc3fvqv99 (2019). Accessed October 3 2019.
51. University of South Florida College of Public Health: DrPH 2019-2020Plan of StudyConcentration in PublicHealth and Clinical Laboratory Science and Practice. https://usf.app.box.com/s/7d98f09e9adpl342do5g165rbsaoiypl (2019). Accessed October 3 2019.

52. University of Texas Health Science Center at Houston School of Public Health: Doctor of Public Health (DrPH). https://sph.uth.edu/academics/degree-programs/doctor-of-public-health-drph/ (2019). Accessed August 13 2019.
