# Supplementary material for: Analysis of CEPH-accredited DrPH programs in the United States: A mixed-methods study
Source: PLoS One. 2021 Feb 4;16(2):e0245892. doi: 10.1371/journal.pone.0245892 (PMC7861440; doi:10.1371/journal.pone.0245892)
Supplement: S8 Table — (PDF) [file pone.0245892.s008.pdf]

S8 Table. Internal funding information

School Name	Internal Funding	Internal Funding resource website or handbook
1. Boston University School of Public Health	▪ For incoming DrPH students: school offers tuition scholarships.<br>▪ For current DrPH students: various doctoral training grants, including the HRSA/MCHB-funded MCH Applied Research Fellowship Program, are available.<br>▪ For part-time DrPH students: the tuition remission program is available.	https://www.bu.edu/sph/admissions/financing-your-education/
2. Claremont Graduate University School of Community & Global Health	There are three fellowships provided to DrPH students: Departmental Fellowships, Blaisdell Fellowships, and Trustee Fellowship Awards.	https://www.cgu.edu/academics/program/doctor-public-health/cost-aid/
3. University of Colorado School of Public Health	School provides Public Health Scholarships and Global Health Scholarships to graduate students.	http://www.ucdenver.edu/academics/colleges/PublicHealth/research/centers/globalhealth/education/Pages/scholarships.aspx
4. Columbia University Mailman School of Public Health	School provides scholarships, fellowships, and grants as well as five-year funding packages.	https://www.mailman.columbia.edu/become-student/how-apply/financial-aid/funding-options/scholarships-fellowships-and-grants
5. Drexel University Dornsife School of Public Health	School provides scholarships, financial aid, teaching and/or research assistantships, and fellowships.	▪ https://drexel.edu/dornsife/admissions/apply/faqs<br>▪ https://drexel.edu/dornsife/admissions/scholarships-tuition-financial-aid/
6. East Tennessee State University College of Public Health	School provides a Graduate Assistantship and Tuition Scholarship.	https://www.etsu.edu/cph/documents/drphhandbook.pdf
7. George Washington University Milken Institute School of Public Health	▪ School provides Graduate Assistantship funding to DrPH students.<br>▪ There are funding, scholarship opportunities	▪ https://publichealth.gwu.edu/content/funding-current-students#Dept<br>▪ https://www.jhsph.edu/admissions/funding-opportunities

School Name	Internal Funding	Internal Funding resource website or handbook
8. Georgia Southern University Jiann-Ping Hsu College of Public Health	School provides scholarships and Graduate Assistantship.	https://jphcoph.georgiasouthern.edu/students/assistantships/
9. Georgia State University School of Public Health	Funding is not available for the DrPH program. School refers to Financial Aid Office.	https://sfs.gsu.edu/
10. Harvard T.H. Chan School of Public Health	▪ An anonymous donor provided funding when the new DrPH program was launched.<br>▪ There is no scholarship or fellowship specifically for DrPH students. Instead, school encourages students to explore all avenues of funding opportunities.	https://www.hsph.harvard.edu/osfs/scholarship-information/
11. Johns Hopkins Bloomberg School of Public Health	The Bloomberg American Health Initiative provides full-tuition scholarships to 10 Bloomberg Doctoral (DrPH) Fellows each year.	https://americanhealth.jhu.edu/fellowship/doctoral-program
12. Loma Linda University School of Public Health	School provides Selma Andrews travel funds and Selma Andrews scholarship funds to School of Public Health students.	https://home.llu.edu/sites/home.llu.edu/files/docs/student-handbook.pdf
13. New York Medical College School of Health Sciences and Practice & Institute of Public Health	School provides special arrangement (e.g., scholarship), but there is no information available in public.	https://www.nymc.edu/current-students/student-services/bursar/tuition-and-fees/school-of-health-sciences-and-practice/
14. Pennsylvania State University College of Medicine Public Health Program	School provides Graduate Assistantships.	https://med.psu.edu/drph/tuition
15. SUNY Downstate Medical Center School of Public Health	School provides scholarship and fellowship.	https://sls.downstate.edu/financial_aid/
16. Texas A&M School of Public Health	School offers scholarship and assistantship.	https://sph.tamhsc.edu/future/financial.html

School Name	Internal Funding	Internal Funding resource website or handbook
17. Tulane University School of Public Health and Tropical Medicine	School offers fellowships and other student fundings.	https://catalog.tulane.edu/public-health-tropical-medicine/global-community-health-behavioral-sciences/global-community-health-science-behavior-dph/#text
18. University at Albany School of Public Health	School provides grants and scholarships to DrPH full-time students.	▪ https://www.albany.edu/sph/assets/2017-2018_Graduate_Handbook_FINAL.pdf<br>▪ https://www.albany.edu/graduatebulletin/public_health_drph_degree.htm
19. University of Alabama at Birmingham School of Public Health	▪ Biostatistics department provides fellowships, traineeships, and/or assistantship.<br>▪ School provides scholarships.	▪ https://www.soph.uab.edu/files/Student%20Handbooks/2018/BST_Graduate_Handbook_2018.pdf<br>▪ https://uab.academicworks.com/
20. University of Arizona Mel and Enid Zuckerman College of Public Health	School provides teaching and research assistantship and scholarships to graduate students.	▪ School-wide: https://publichealth.arizona.edu/graduate-students/tuition-financial-assistance<br>▪ Maternal and Child Health Program: https://publichealth.arizona.edu/sites/publichealth.arizona.edu/files/MCH%202018-19%20Handbook.pdf
21. University of Arkansas for Medical Sciences Fay W. Boozman College of Public Health	School provides scholarships.	https://publichealth.uams.edu/students/current-students/student-resources/general-public-health-scholarships/
22. University of California Berkeley School of Public Health	▪ School offers grants (Graduate Student Researcher and Graduate Student Instructor) and graduate fellowships and scholarships.<br>▪ DrPH candidates may also receive a one-time stipend from the Grossman Endowment.<br>▪ DrPH candidates receive a 100% reduction in the annual nonresident tuition for a maximum	▪ https://sph.berkeley.edu/admissions/fees-financial-aid<br>▪ http://sph.berkeley.edu/sites/default/files/2018_SPH_student-handbook_08-16-18.pdf

School Name	Internal Funding	Internal Funding resource website or handbook
	of 3 calendar years (from the semester that they advanced), whether registered or not.	
23. University of Georgia College of Public Health	School provides scholarship, awards, and graduate assistantships.	https://publichealth.uga.edu/about/financial-aid/
24. University of Illinois at Chicago School of Public Health	School offer scholarships, assistantships, and fellowships.	https://publichealth.uic.edu/admissions-aid/financial-aid
25. University of North Carolina Gillings School of Global Public Health	School provides fellowship and scholarship.	https://gradschool.unc.edu/funding/gradschool
26. University of Puerto Rico Graduate School of Public Health	School provides financial aid office information, but there is no clear information for DrPH students.	http://www.rcm.upr.edu/wp-content/uploads/sites/3/2018/10/UPR-MSC-CATALOG-2017-2020-REV-October-22-2018-SECURED.pdf
27. University of South Florida College of Public Health	School provides Graduate Assistantships, Graduate Research Assistantships, and various types of scholarships.	http://health.usf.edu/publichealth/academicaffairs/FinAid
28. University of Texas Health Science Center at Houston School of Public Health	School provides Graduate Research Assistantships, Teaching Assistantships, and various types of scholarships.	https://sph.uth.edu/prospective-students/prospective-student-faqs/
